# Design, synthesis and biological evaluation of novel histone deacetylase (HDAC) inhibitors derived from *β*-elemene scaffold

**DOI:** 10.1080/14756366.2023.2195991

**Published:** 2023-04-04

**Authors:** Yuan Gao, Jilong Duan, Xiawen Dang, Yinghui Yuan, Yu Wang, Xingrui He, Renren Bai, Xiang-Yang Ye, Tian Xie

**Affiliations:** a Institute of Traditional Chinese Medicine, Shanghai University of Traditional Chinese Medicine, Shanghai, China; b Key Laboratory of Elemene Class Anti-Cancer Chinese Medicines; Engineering Laboratory of Development and Application of Traditional Chinese Medicines, Collaborative Innovation Center of Traditional Chinese Medicines of Zhejiang Province, Hangzhou Normal University, Hangzhou, China; c Research and Development, Dalian HolleyKingkong Pharmaceutical Co. Ltd, Liaoning, China

**Keywords:** *β*-Elemene, Histone deacetylase inhibitor, Hybrid drugs, Lymphoma

## Abstract

*β*-Elemene is the major active ingredient of TCM anticancer drug elemene extracts. To further improve its antitumor activity and poor solubility, a polar HDACi pharmacophore was incorporated its scaffold. Systematic SAR studies led to the discovery of compounds **27f** and **39f**, which exhibited potent inhibitory activity against HDACs (HDAC1: IC_50_ = 22 and 9 nM; HDAC6: 8 and 14 nM, respectively). In cellular levels, **27f** and **39f** significantly inhibited cell proliferation of five tumour cell lines (IC_50_: 0.79 - 4.42 µM). Preliminary mechanistic studies indicated that **27f** and **39f** efficiently induced cell apoptosis. Unexpectedly, compound **39f** could also stimulate cell cycle arrest in G1 phase. Further *in vivo* study in WSU-DLCL-2 xenografted mouse model validated the antitumor activities of **27f**, without significant toxicity. The results suggest the therapeutic potential of these HDACs inhibitors in lymphoma and provide valuable insight and understanding for further structural optimisation around *β*-elemene scaffold.

## GRAPHICAL ABSTRACT

**Figure:**
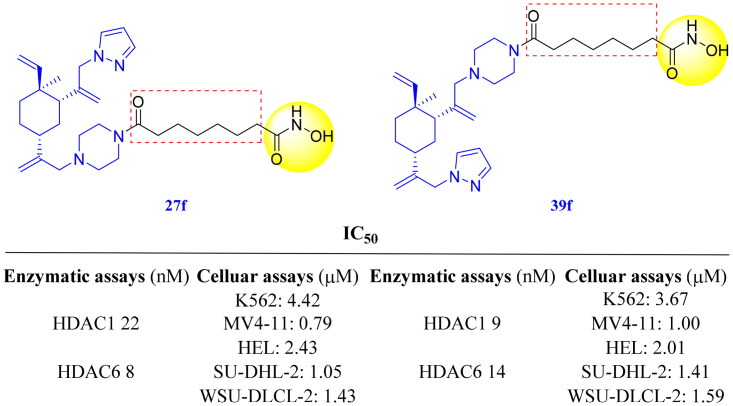

## Introduction

**Figure 1.**
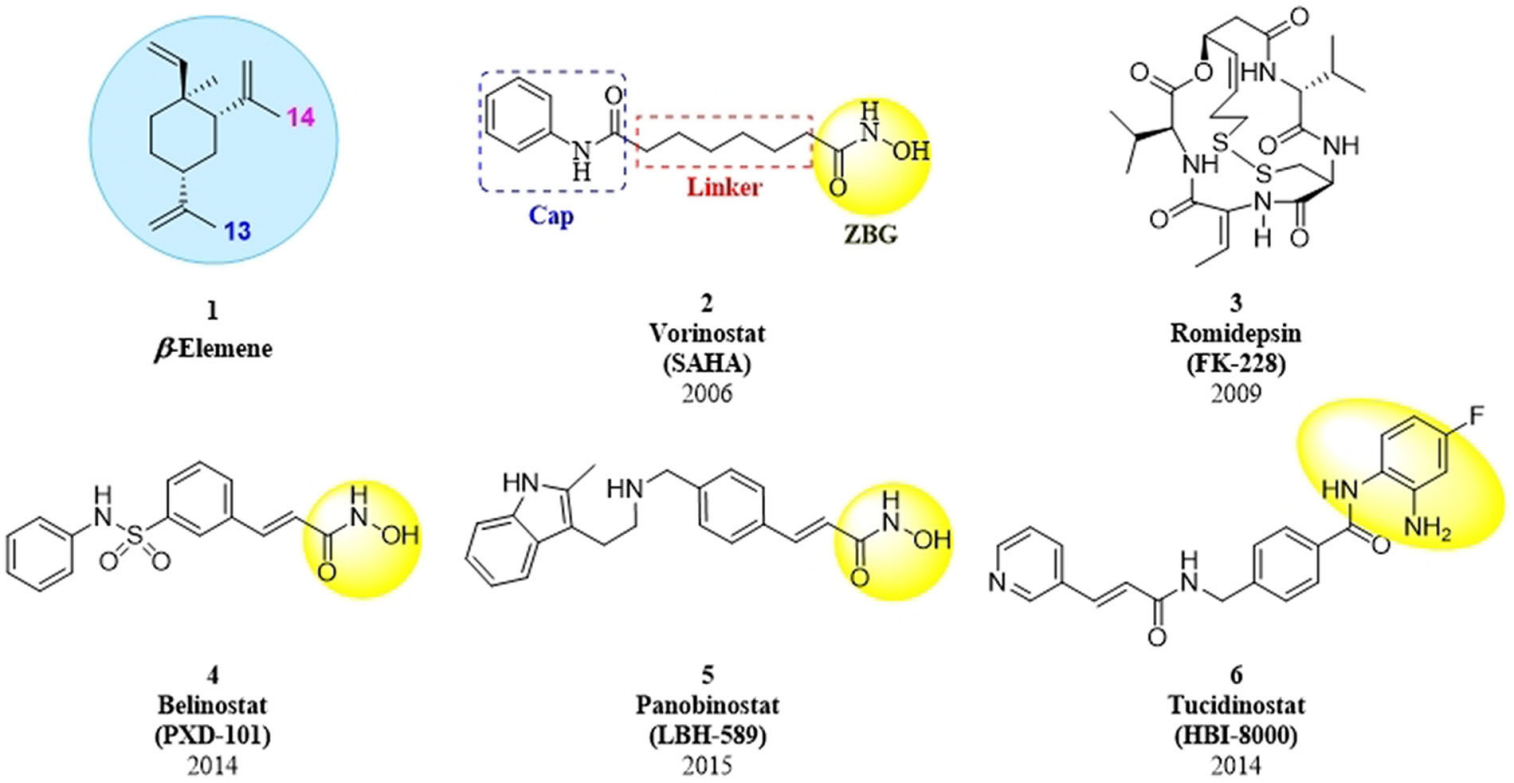
The structures of *β*-elemene (**1**) and approved HDAC inhibitors (**2–6**).

*β*-Elemene (Figure 1, 1) is a sesquiterpene extracted from the rhizome of *Curcuma wenyujin*^1^^,^^2^. It has been used in the treatment of lung cancer, pancreatic cancer, gastric cancer, breast cancer, bladder cancer, and malignant brain glioma^3–8^. Studies have shown that *β*-elemene inhibits tumour cell growth *via* diverse mechanisms^9^^,^^10^, including induction of apoptosis, autophagy and cell cycle arrest, and inhibition of cell proliferation and migration, etc. However, its clinical application has been limited by poor solubility, low bioavailability, and moderate antitumor activity^11–14^. To overcome these shortcomings, the combination therapy of *β*-elemene with other anticancer drugs, such as cisplatin^15^, paclitaxel^16^, and gefitinib^17^ have been extensively studied. On the other hand, the structural modifications based on *β*-elemene scaffold such as the hydroxyl carboximates derivatives^18^, vinylated derivatives^19^, *NO*-donating derivatives^20^ and macrocycles derivatives ^21^ have been a major trend for identifying new chemical entities with better anticancer effects and potential better druggability than *β*-elemene. The above mentioned cancer treatment paradigms have achieved great success and broad prospects in the treatment of solid tumours, but has poorly reported for the treatment of lymphoma.

Histone deacetylases (HDACs) are a family of enzymes that catalyse the deacetylation of lysine residues of both histone and non-histone protein substrates^22^^,^^23^. The expression levels of HDACs are usually higher in tumour cells than in normal cells^24^. Inhibition of HDACs can lead to pleiotropic effects on cellular signalling and transcription, including cell death, differentiation, autophagy, and enhancement of immunogenicity^25–30^. To date, five drugs are approved for the treatment of various types of haematologic malignancies: vorinostat (SAHA, **2**), romidepsin (FK-228, **3**), belinostat (PXD-101, **4**), panobinostat (LBH-589, **5**), and tucidinostat (HBI-8000, **6**), as shown in Figure 1. These HDAC inhibitors demonstrated excellent antitumor effect, but generally limited by a narrow antitumor spectrum, drug resistance, and undesirable side effects^31–35^. To address these issues, HDAC inhibitors are clinically used in combination with other antitumor agents to achieve synergistic or additive effects and reduce the potential for developing drug resistance^36–38^. Although drug combinations have the advantages of higher antitumor efficacy, they also suffer from enhanced adverse effects, unpredictable pharmacokinetic profiles, drug-drug interactions, and poor patient compliance^36^. In recent years, the designed and synthesised hybrid drugs, comprising the HDAC inhibitor (HDACi) pharmacophores, are emerging as an alternative strategy to drug combinations^24^^,^^39^. Scientific studies have shown that many hybrid agents had more potent anti-proliferation effects than the corresponding single agent^40–45^, which encouraged and promoted us to develop the hybrid molecules of *β*-elemene and HDAC inhibitor.

Based on the above information and our experience in the structural modification of *β*-elemene, we have designed and synthesised novel HDAC inhibitors derived from the *β*-elemene scaffold. Herein, we reported the design, synthesis and biological evaluation of the novel HDACi derived from the *β*-elemene scaffold.

## Results and discussion

### Design

**Figure 2.**
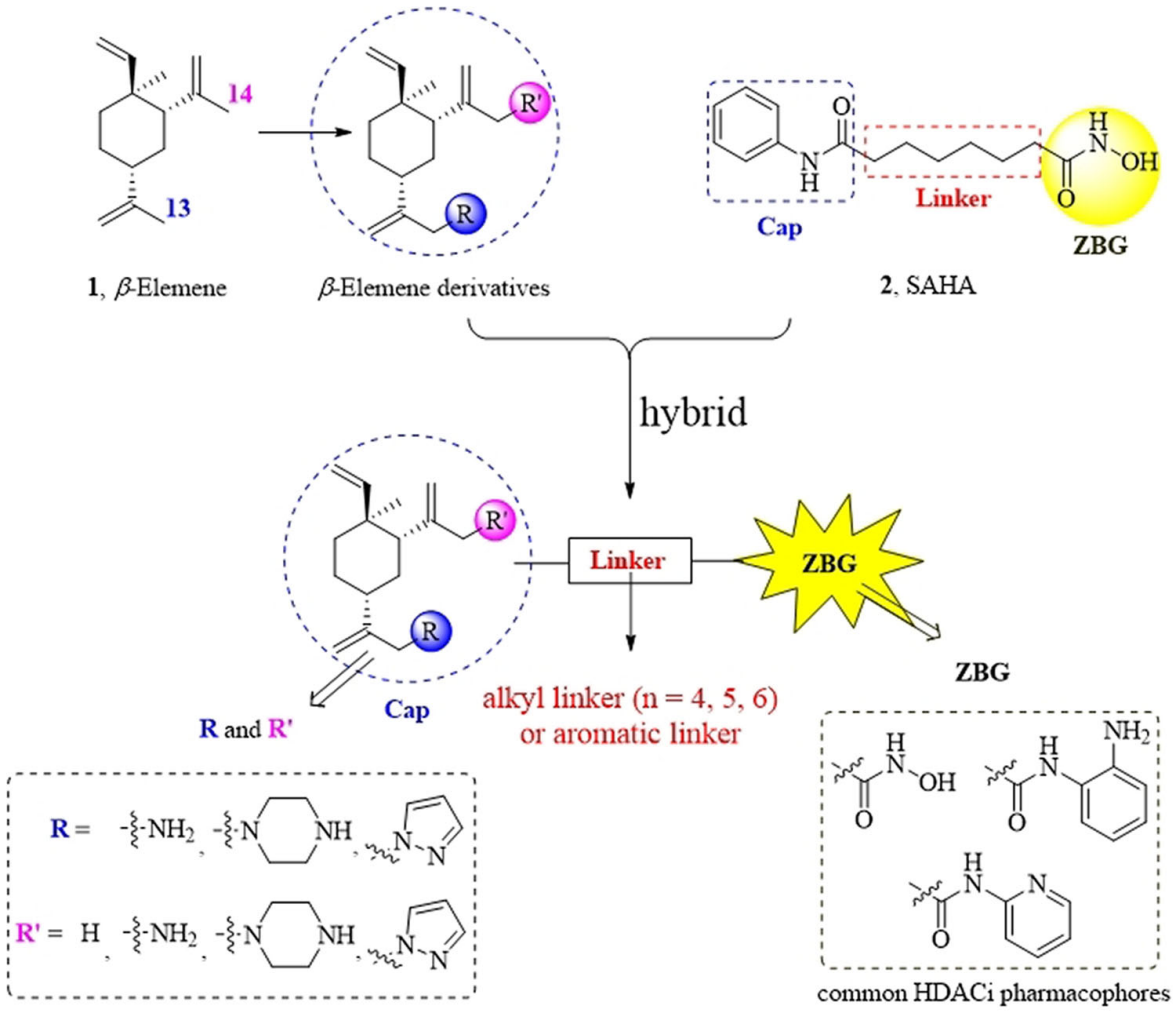
The designing strategy of HDACi derived from *β*-elemene scaffold.

Generally, the pharmacophores of HDACi are divided into three key components: the cap group (acts as a surface binding, Cap), the linker, and the pharmacophore (zinc-binding group, ZBG) (**2**, Figure 2). The cap portion, which accounts for the affinity gain through hydrophobic interaction with protein, can be large or small in size and can tolerate diverse structures. We envisioned that the lipophilic antitumor *β*-elemene scaffold should be well tolerated as a cap portion and might potentially provide some beneficial effects to achieve better anticancer activity through “hybrid” drug concept (Figure 2). The linker is typically linear and hydrophobic as well (e.g. straight carbon chain, *trans-* di-substituted olefin or di-substituted phenyl or heteroaryl are often used as the linker). In this study, the different carbon atoms “alkyl linker” and “aromatic linker” was used as the “Linker” to connect the *β*-elemene derivatives and the “ZBG” portion. The “ZBG” group (also known as pharmacophore or warhead) of many HDACi is hydroxamic acid, and those of few HDACi can also be acyl aniline, cyclic tetrapeptides, thiol, and aliphatic carboxylic acids, or their isosteric replacements. In this work, we examined three common pharmacophores as “ZBG”: hydroxamic acid, acyl aniline and acyl pyridine. As a result, six series of HDACi derived from *β*-elemene scaffold were designed and synthesised, and their biological activities were evaluated.

### Chemistry

**Scheme 1.**
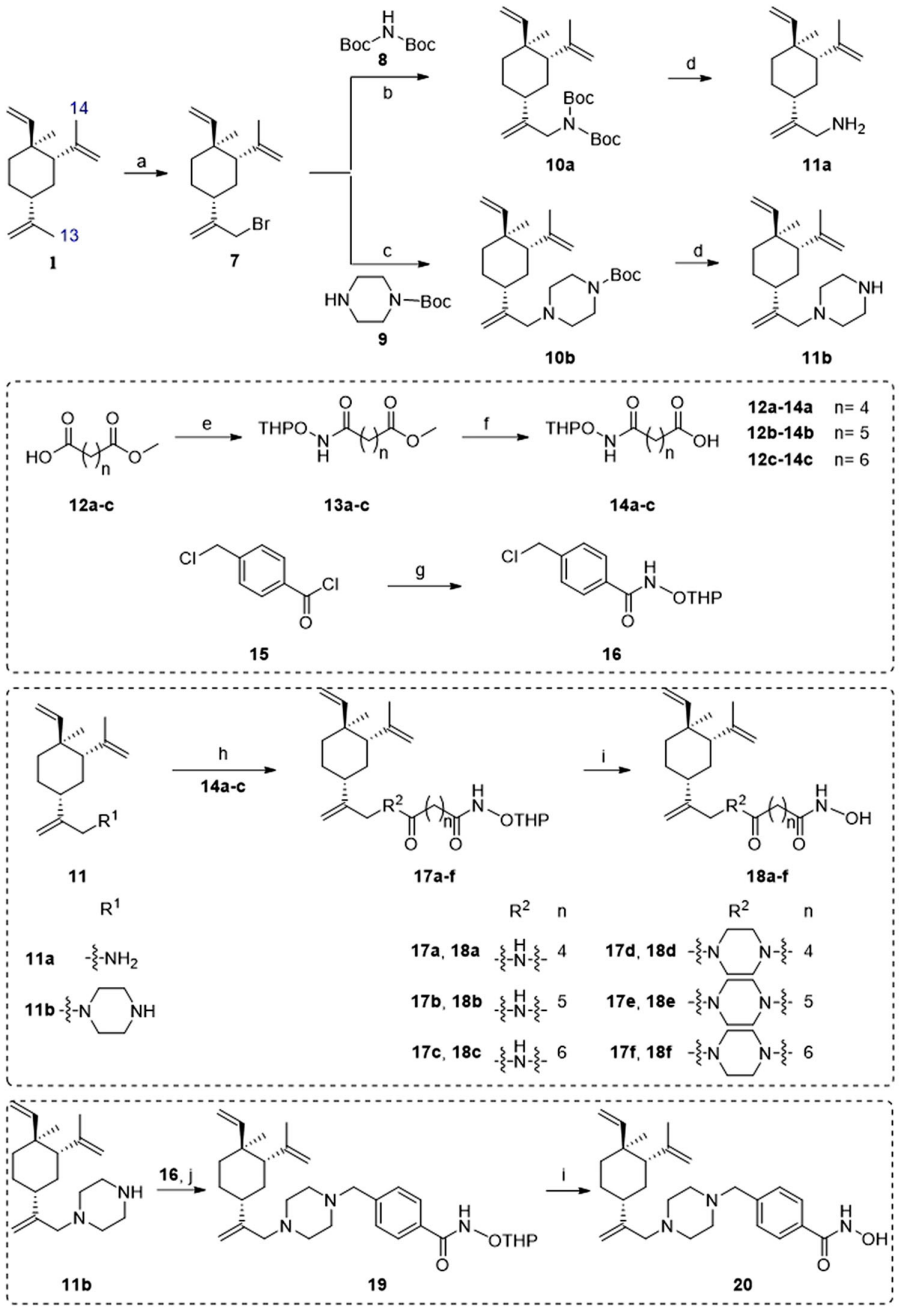
Synthetic routes of the title compounds **18a–f** and **20**. Reaction conditions and reagents: (a) NBS, AcOH, CH_2_Cl_2_, 0 °C to rt, 9 h; (b) Cs_2_CO_3_, DMF, Bis(tert-butoxycarbonyl)amine, 60 °C, 10 h; (c) DIPEA, DMF, 1-Boc-piperazine, 60 °C, 10 h; (d) CF_3_CO_2_H, 0 °C ∼ rt, 5 h; (e) EDCI, HOBT, DIPEA, DMF, NH_2_OTHP, rt, 5 h; (f) NaOH (aq), CH_3_OH, rt, 2 h; (g) CH_2_Cl_2_, NH_2_OTHP, 0 °C, 3 h; (h) EDCI, HOBT, DIPEA, DMF, rt, 5 h; (i) TsOH·H_2_O, CH_3_OH, rt, 8 h; (j) DIPEA, DMF, **16**, 60 °C, 6 h.

The synthesis of target compounds **18a–f** and **20** is illustrated in Scheme 1. Bromination between the starting material *β*-elemene (**1**) and *N*-bromosuccinimide (NBS) yielded 13-Br-*β*-elemene (**7**). The reaction of **7** with different amines gave *N*-Boc protected intermediates **10a–b**. The Boc protecting group was subsequently removed to yield amines **11a–b**. Separately, the condensation between various carboxylic acids **12a–c** and *O*-(Tetrahydro-2*H*-pyran-2-yl)hydroxylamine (NH_2_OTHP) afforded THP-protected intermediates **13a–c**, which underwent ester hydrolysis to give corresponding acids **14a–c**. Then, the amide coupling reaction between amines **11a–b** and carboxylic acids **14a–c** gave intermediates **17a–f**, which underwent subsequent THP deprotection to afford the desired compounds **18a–f**. On the other hand, intermediate **16** was easily obtained from commercially available acyl chloride **15** and NH_2_OTHP. The SN_2_ displacement reaction between **11b** and **16** was facilitated in the presence of DIPEA under heating to afford intermediate **19**, which underwent THP deprotection to give the target compound **20**.

**Scheme 2.**
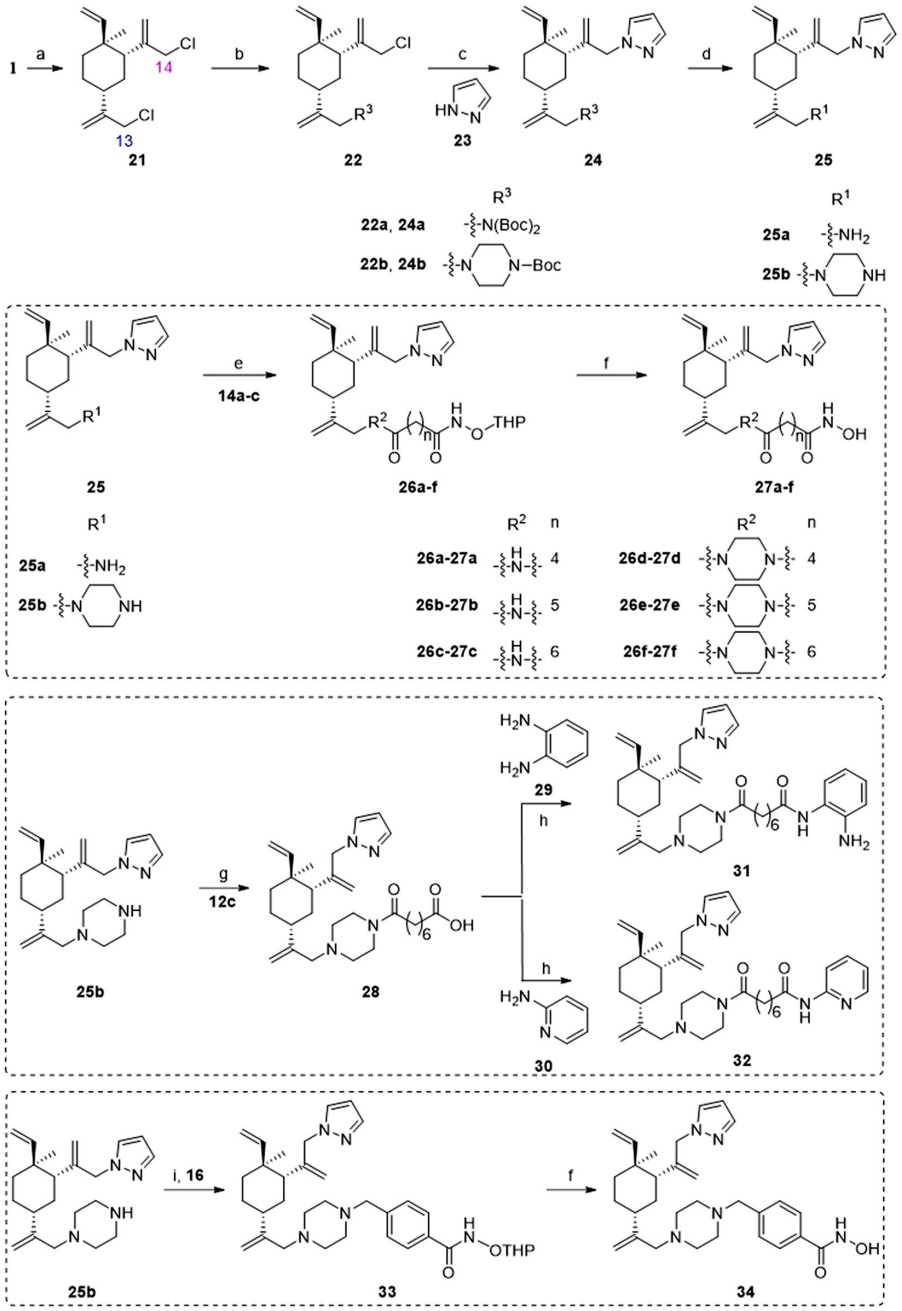
Synthetic routes of the title compounds **27a–f**, **31–32** and **34**. Reaction conditions and reagents: (a) NaClO, AcOH, CH_2_Cl_2_, TBAF, 0 °C, 6 h; (b) Cs_2_CO_3_, DMF, **8** or **9**, 50 °C, 8 h; (c) Cs_2_CO_3_, DMF, **23**, 60 °C, 10 h; (d) HCl-Dioxane (4 M), rt, 8 h; (e) EDCI, HOBT, DIPEA, DMF, **14a–c**, rt, 5 h; (f) TsOH·H_2_O, CH_3_OH, rt, 8 h. (g) (i) EDCI, HOBT, DIPEA, DMF, **12c**, rt, 5 h; (ii) NaOH (aq), CH_3_OH, rt, 2 h; (h) EDCI, HOBT, DIPEA, DMF, rt, 5 h; (i) DIPEA, DMF, **16**, 60 °C, 6 h.

The preparation of analogues **27a–f**, **31–32** and **34** is outlined in Scheme 2. First, *β*-elemene (**1**) was treated with NaClO under the mixed solvent of AcOH and CH_2_Cl_2_ to furnish 13,14-dichloro-*β*-elemene (**21**). It was discovered by us that the two allylic chloro groups had differentiated reactivity in the presence of a nucleophile. This is presumably due to the stereo effects in the chair-like conformation of cyclohexane ring, in which slightly more bulky around 14-position in **21** leads to less reactivity than 13-position^46^. By carefully controlling the reaction conditions, two different R^3^ were installed at position 13- of **21** to yield intermediate **22**. Subsequently, a pyrazole group was installed at position 14- to afford intermediate **24**. The removal of Boc-deprotecting group afforded key intermediates **25a–b**. The transformation of **25a–b** to the target molecules **27a–f** could then be achieved using the similar reaction sequences described in Scheme 1. For the target molecules bearing HDACi pharmacophores other than hydroximate (i.e. **31** and **32**), slightly modified reaction sequence was adopted. Finally, compound **34** was prepared using the similar procedure for compound **20** described in Scheme 1.

**Scheme 3.**
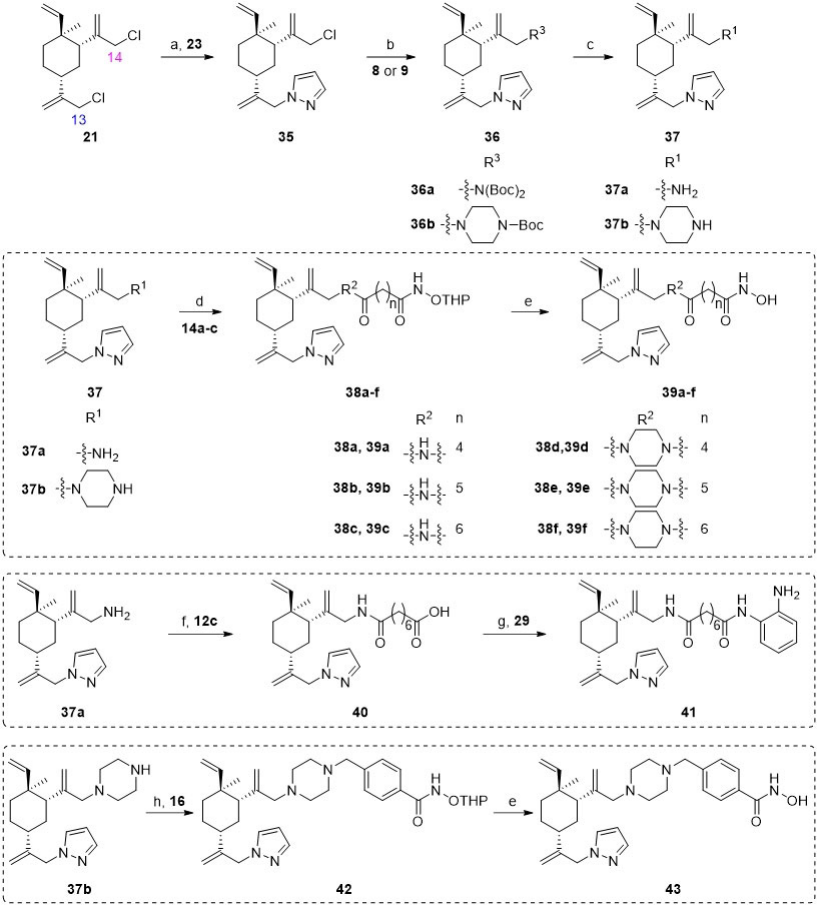
Synthetic routes of the title compounds **39a–f**, **41** and **43**. Reaction conditions and reagents: (a) Cs_2_CO_3_, DMF, **23**, 45 °C, 10 h; (b) Cs_2_CO_3_, DMF, **8** or **9**, 80 °C, 8 h; (c) HCl-Dioxane (4 M), rt, 8 h; (d) EDCI, HOBT, DIPEA, DMF, **14a–c**, rt, 5 h; (e) TsOH·H_2_O, CH_3_OH, rt, 8 h; (f) (i) EDCI, HOBT, DIPEA, DMF, **12c**, rt, 5 h; (ii) NaOH (aq), CH_3_OH, rt, 2 h; (g) EDCI, HOBT, DIPEA, DMF, **29**, rt, 5 h; (h) DIPEA, DMF, **16**, 60 °C, 6 h.

Analogues **39a–f**, **41** and **43** were designed to examine the anticancer activity of different attaching positions in comparison with compounds **27a–f**, **31**–**32**, and **34**. The synthetic route for preparing these compounds is depicted in Scheme 3. The reactions used are similar to those discussed in Scheme 2, except some of the sequences were reversed.

### Biological evaluation

#### *In vitro* HDAC inhibition assay

With several different series of target molecules in hand, assays to evaluate enzymatic inhibitions against HDAC were established. Among all HDAC subtypes, HDAC1 and HDAC6 represent Class I and II, respectively. Small molecules inhibiting both HDAC1 and HDAC6 are typically considered as pan-HDAC inhibitors. On the other hand, when the inhibitors have higher activity against HDAC6 than HDAC1, they are considered as HDAC6-selective inhibitors. Therefore, HDAC1 and HDAC6 were selected for the evaluation of the target molecules (Table 1). Most of the compounds showed moderate to excellent inhibitory activities against HDAC1 and HDAC6. For the compounds bearing alkyl linkers and hydroxamic acid as “ZBG” (i.e. **18a–f**, **27a–f** and **39a–21f**), the length of alkyl linker had a great influence on the HDAC1 and HDAC6 inhibitory activities. For instance, four methylene linker analogs (**18a**, **18d**, **27a**, **27d**, **39a** and **39d**) had poor HDAC1 and HDAC6 inhibitory activities. As the linker length increased, the activities of target molecules improved gradually (**18c** ≈ **18b** > **18a**, **18f** > **18e** > **18d**, **27c** > **27b** > **27a**, **27f** > **27e** > **27d**, **39c** > **39b** > **39a** and **39f** > **39e** > **39d**). Interestedly, compounds with aromatic linkers (i.e. **20**, **34** and **43**) are potent HDAC6-selective inhibitors with 20–64-fold selectivity over HDAC1 (351 nM ≤ IC_50_ ≤ 1120 nM for HDAC1; 11 nM ≤ IC_50_ ≤ 55 nM for HDAC6). It’s known that HDAC6-selective inhibitors could have potential therapeutic benefits for lower toxicity and side effects^23^, which is worth further investigation in the future.

**Table 1.** The structures and corresponding IC_50_ values of the target compounds against HDAC1 and HDAC6. 
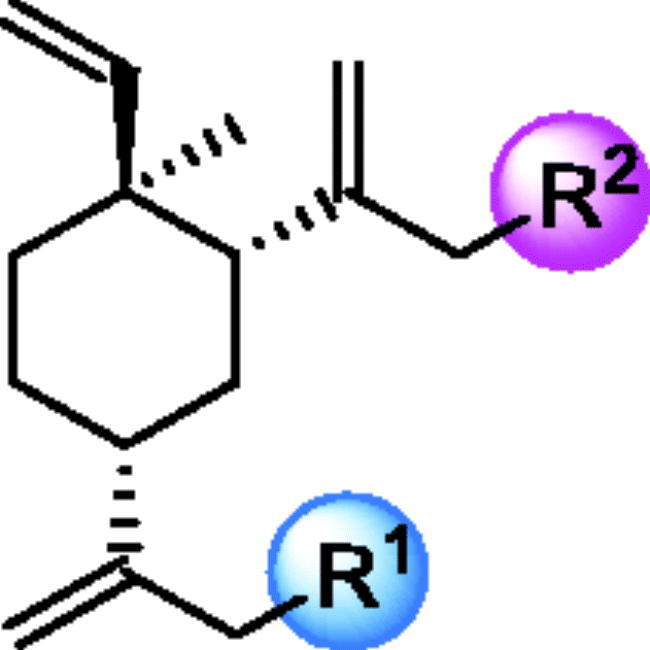

Compd.	R^1^	R^2^	IC_50_ (μM)	
			HDAC1^a^^,b^	HDAC6^a^^,b^
**18a**	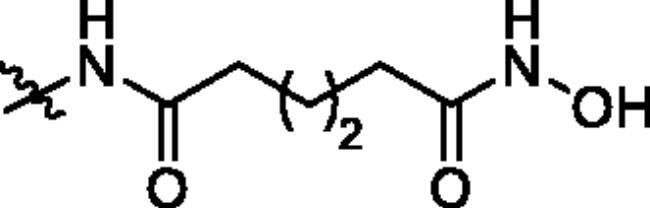	H	0.567 ± 0.043	0.193 ± 0.022
**18b**	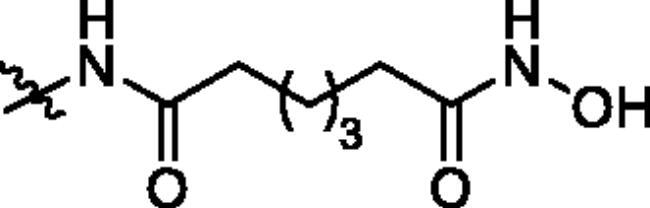	H	0.041 ± 0.006	0.010 ± 0.003
**18c**	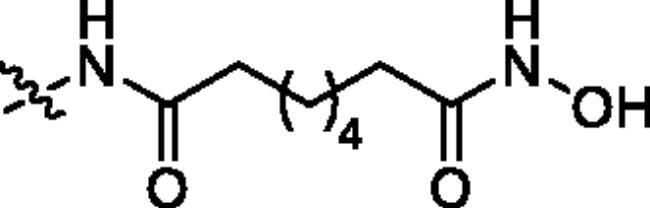	H	0.056 ± 0.012	0.009 ± 0.001
**18d**	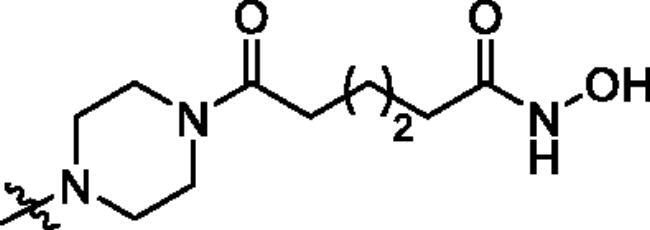	H	0.900 ± 0.078	0.532 ± 0.027
**18e**	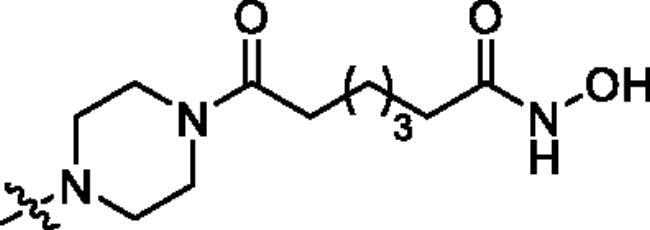	H	0.461 ± 0.066	0.039 ± 0.013
**18f**	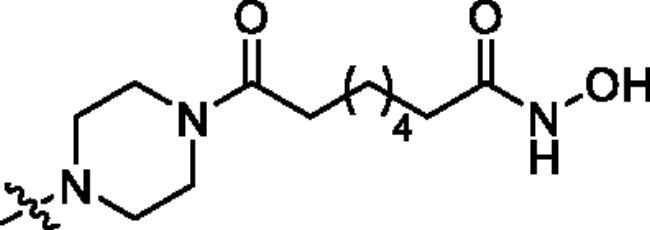	H	0.081 ± 0.008	0.032 ± 0.009
**20**	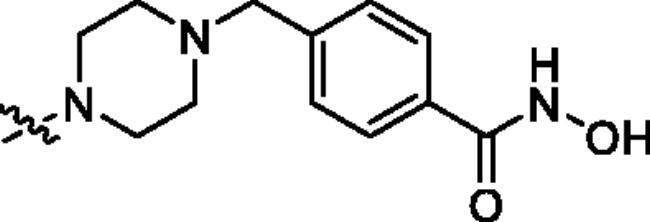	H	1120 ± 0.072	0.055 ± 0.002
**27a**	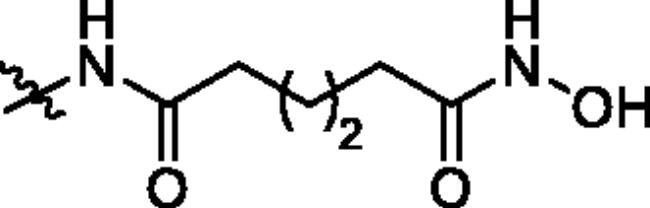	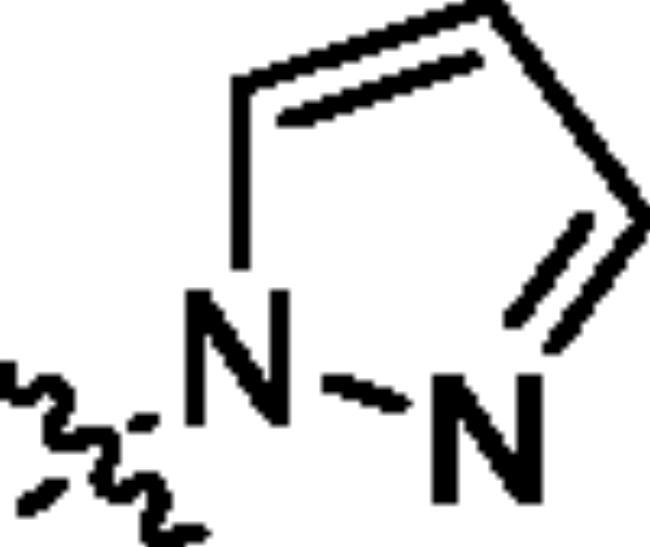	0.879 ± 0.056	0.261 ± 0.004
**27b**	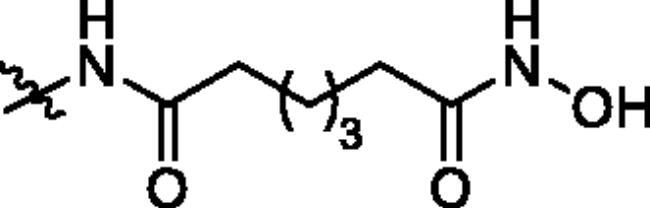	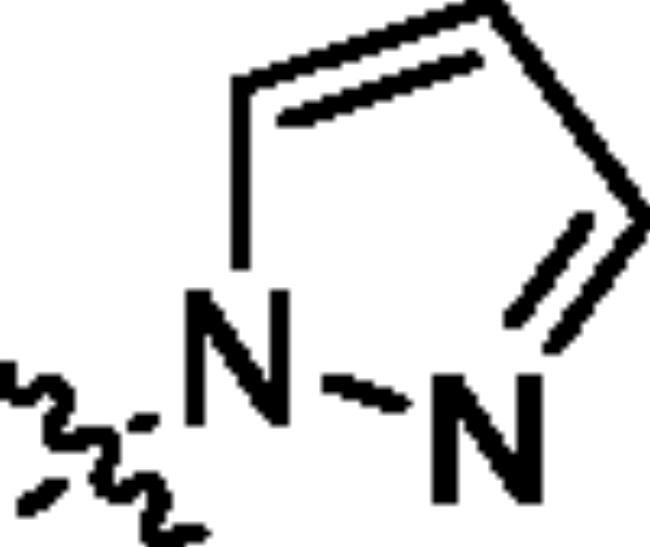	0.061 ± 0.013	0.021 ± 0.007
**27c**	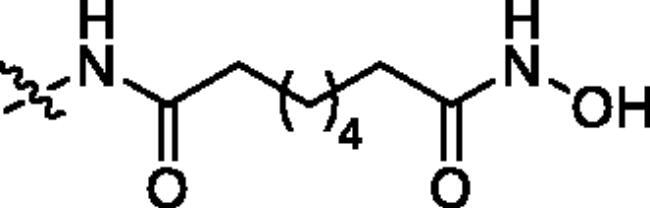	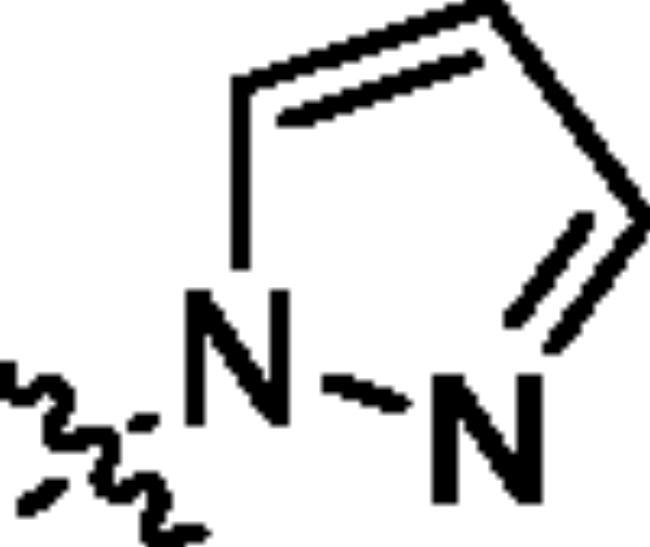	0.053 ± 0.011	0.015 ± 0.005
**27d**	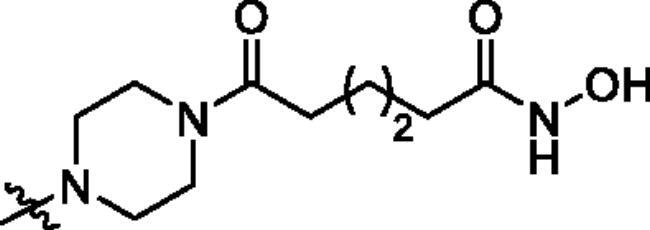	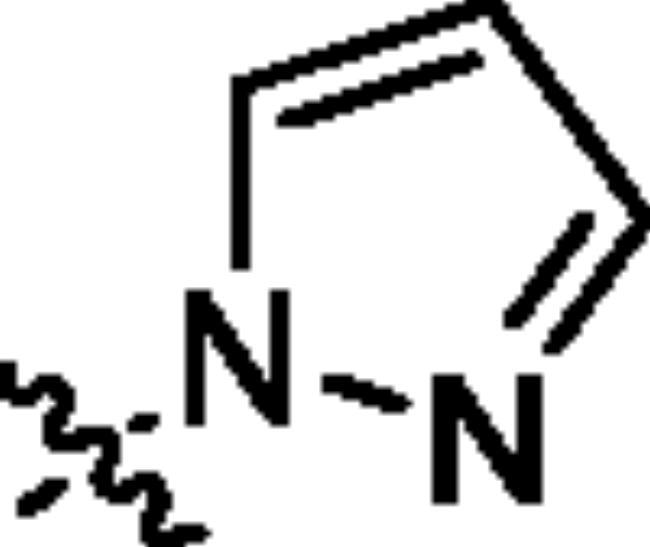	0.858 ± 0.045	0.522 ± 0.081
**27e**	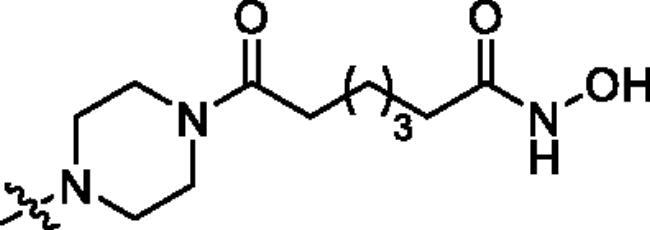	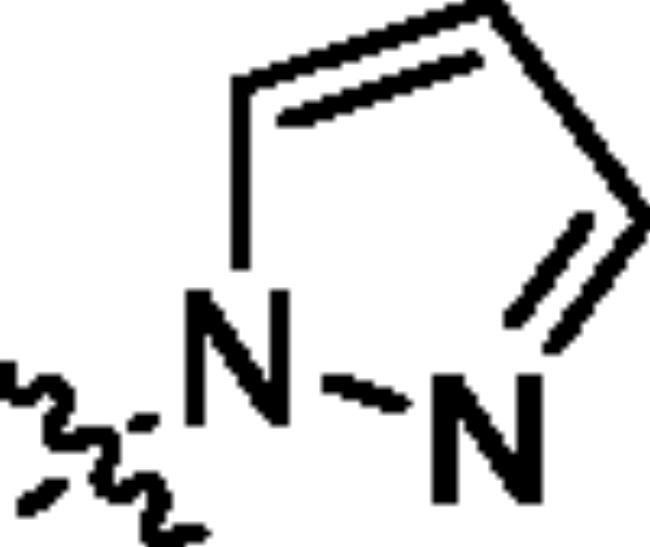	0.189 ± 0.026	0.032 ± 0.006
**27f**	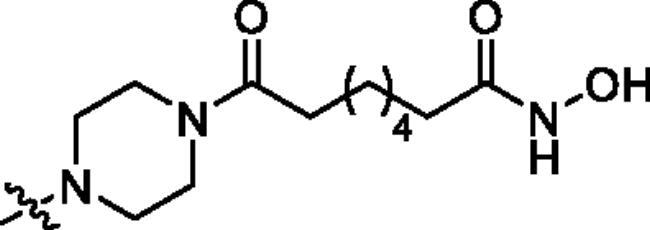	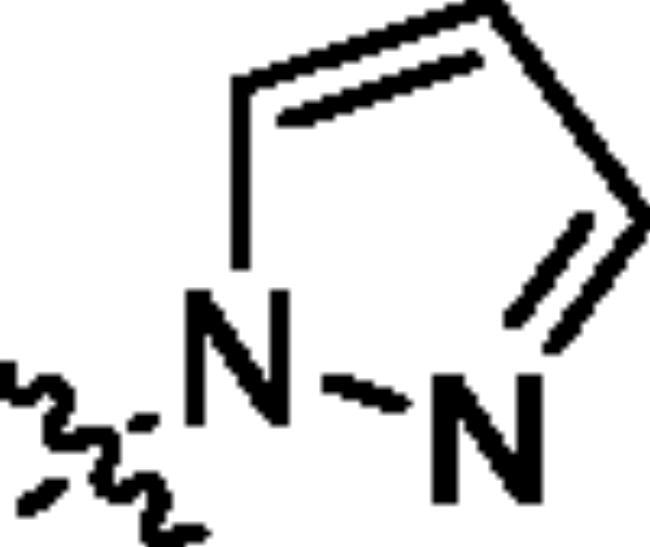	0.022 ± 0.004	0.008 ± 0.001
**31**	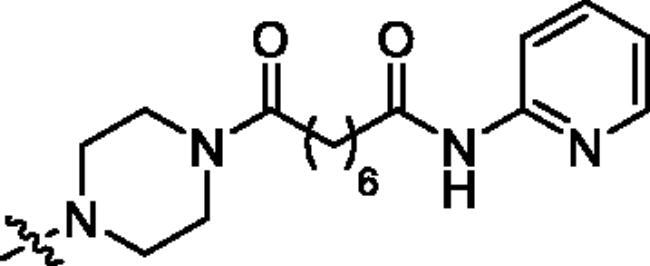	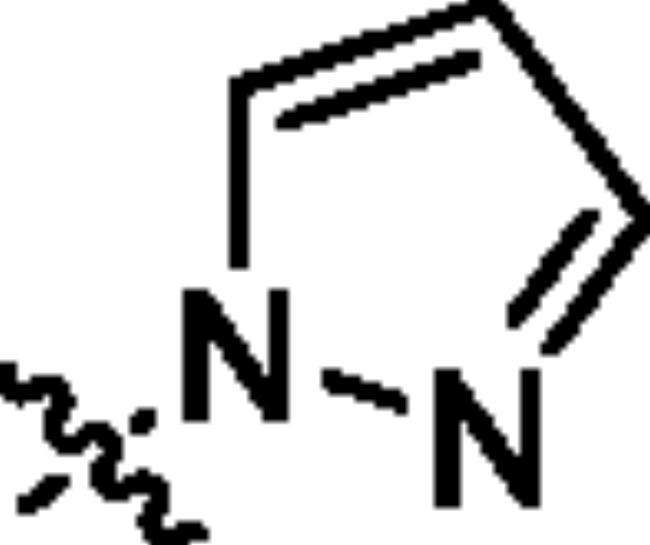	> 5.0	> 5.0
**32**	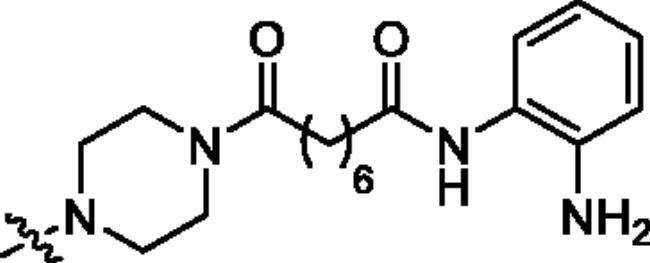	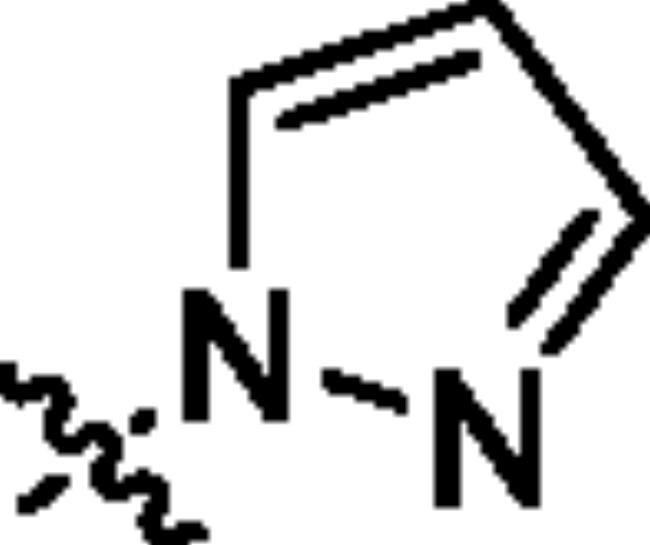	3.544 ± 0.195	> 5.0
**34**	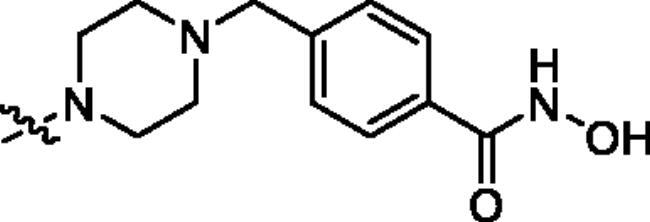	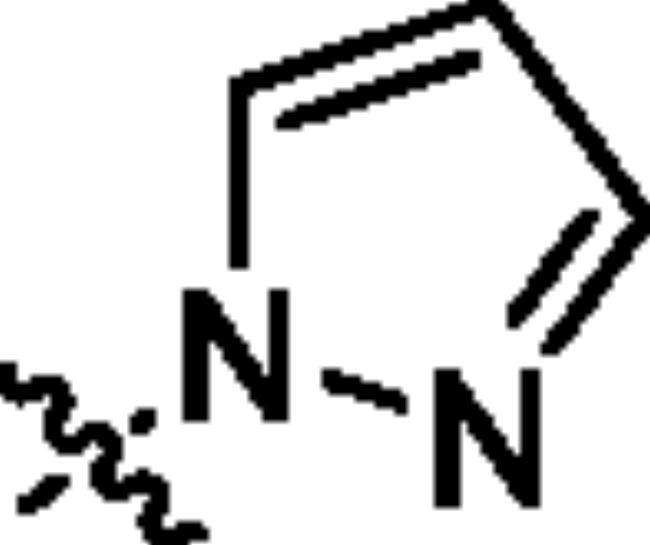	0.351 ± 0.037	0.012 ± 0.002
**39a**	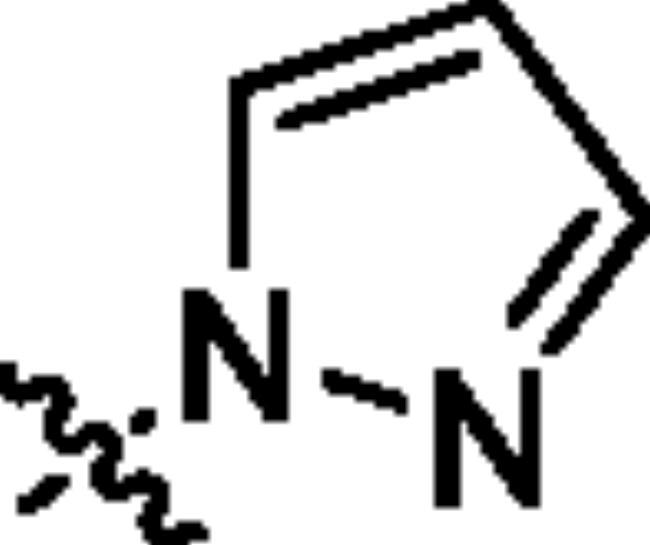	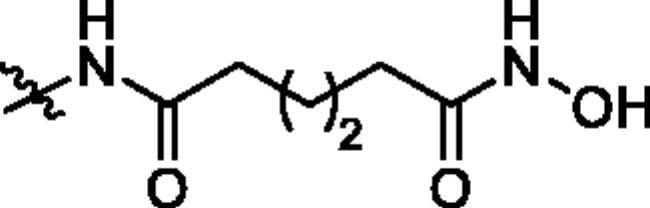	0.986 ± 0.013	0.398 ± 0.041
**39b**	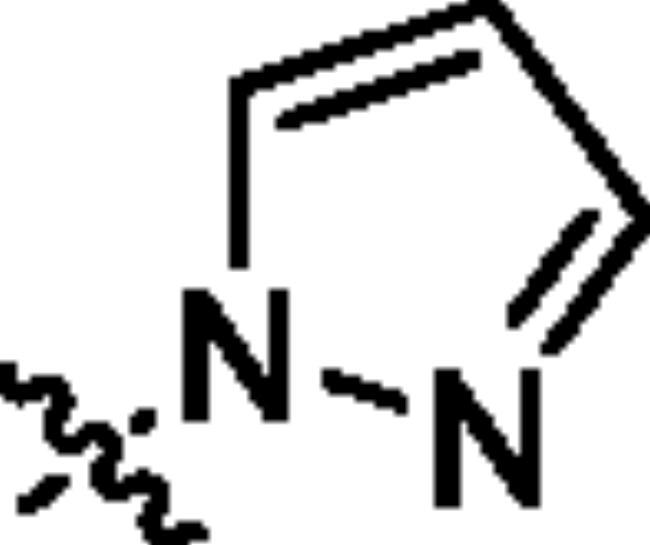	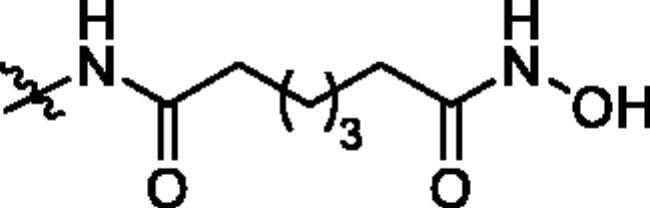	0.136 ± 0.033	0.020 ± 0.007
**39c**	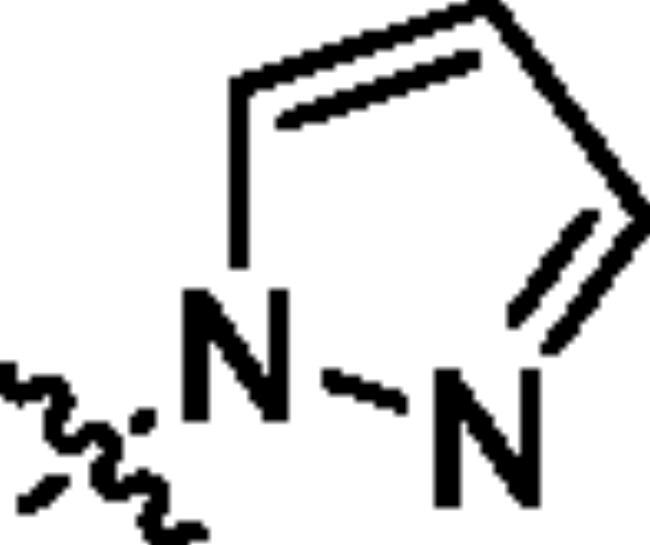	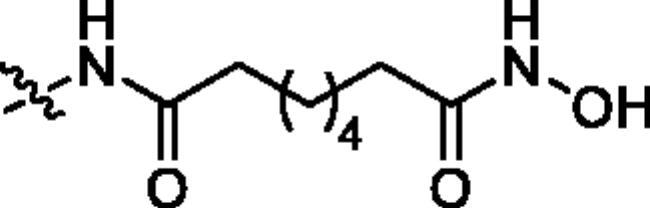	0.050 ± 0.003	0.021 ± 0.005
**39d**	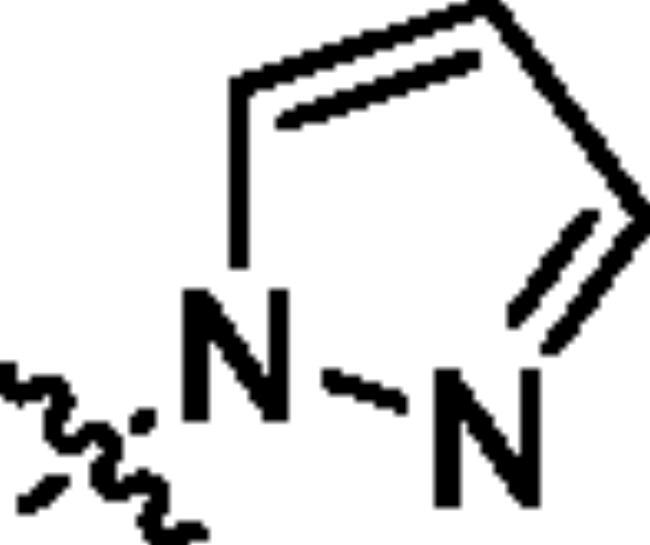	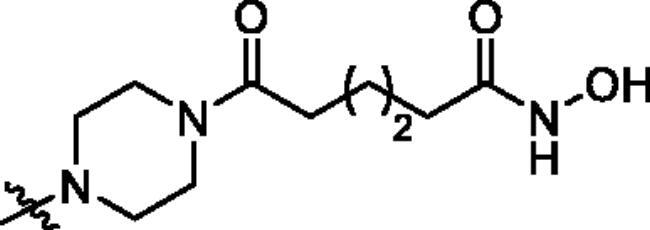	> 5.0	> 5.0
**39e**	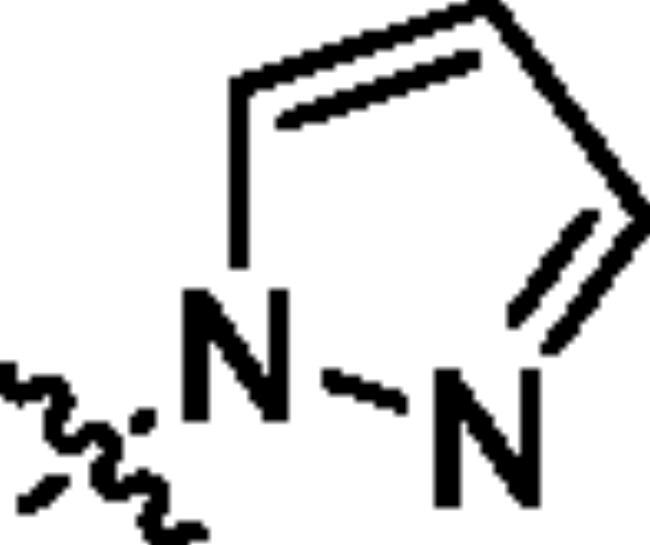	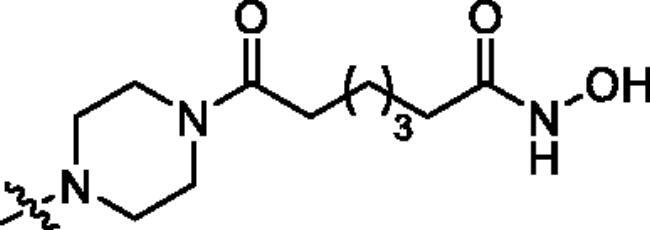	0.070 ± 0.008	0.024 ± 0.004
**39f**	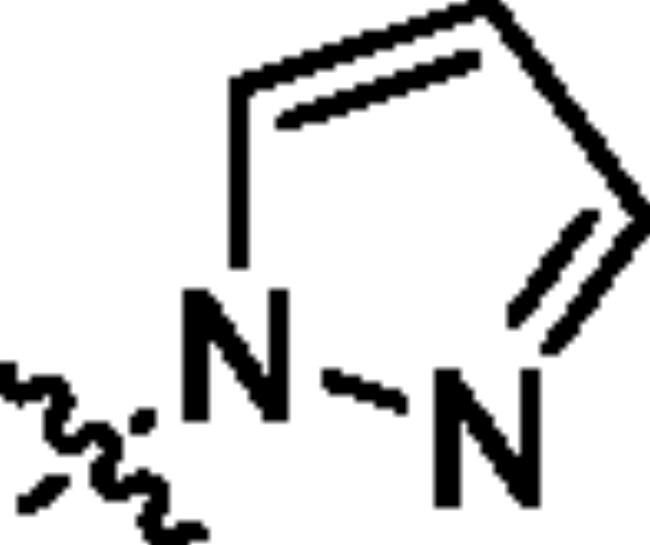	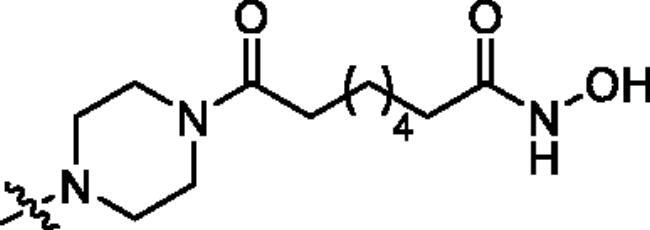	0.009 ± 0.004	0.014 ± 0.001
**41**	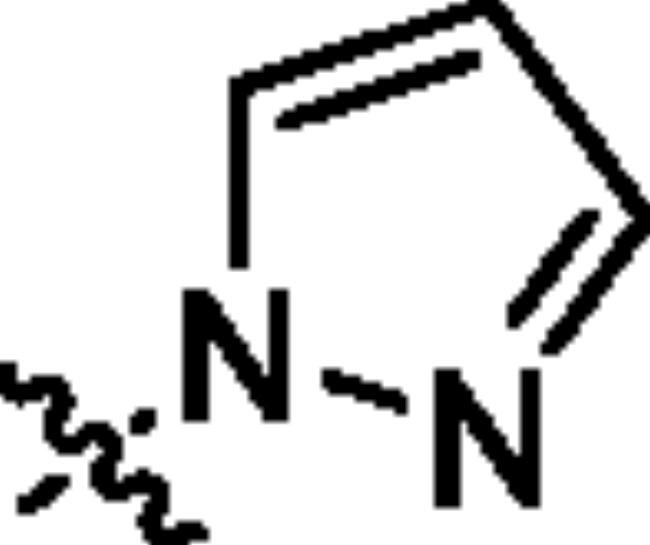	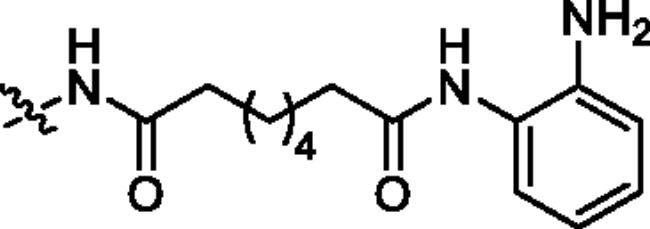	2.134 ± 0.294	> 5.0
**43**	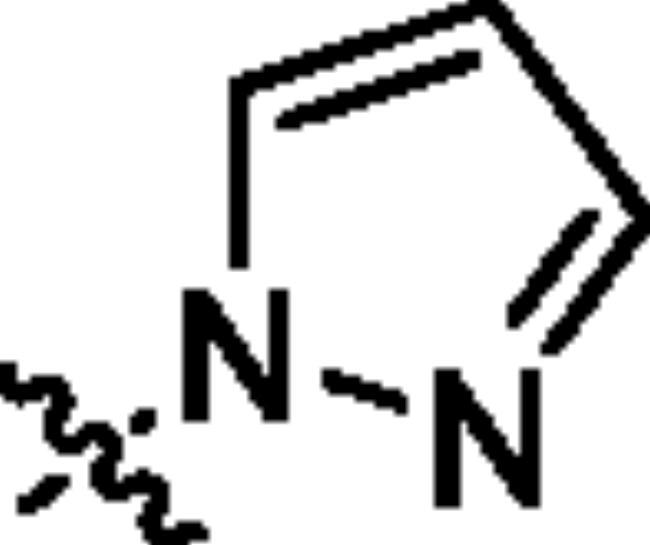	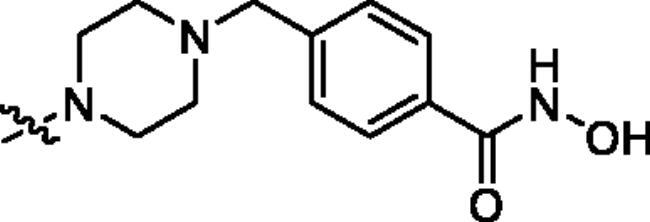	0.710 ± 0.053	0.011 ± 0.006
**1**	H	H	*N.D.*	*N.D.*
**2**	—	—	0.018 ± 0.004	0.012 ± 0.001

^a^Assays were performed in replicate (*n* > 2); IC_50_ values are shown as mean ± SD. ^b^*N.D.* means not determined.

Subsequently, the influence of the different “Cap” groups on the inhibitory activities of HDAC was evaluated. When compounds had the identical “Linker” and “ZBG” group, the following phenomena were observed: (1) Compounds using **11a** as the “Cap” group exhibited better inhibitory activities against HDAC1 and HDAC6 than those compounds using **11b** as the “Cap” group (**18a–c** *vs* **18d–f**). (2) Compounds using **25a** as “Cap” group exhibited comparable inhibitory activities against HDAC1 and HDAC6 compared to those compounds using **25b** as the “Cap” group. The above two series have one common point the pharmacophore and the linker were attached to the 13-position of *β*-elemene. On the other hand, when the pharmacophore and the linker attached to the 14-position of *β*-elemene, the structure-activity relationships (SARs) were varied from case to case. For instance, compound **39a** using **37a** as the “Cap” group showed 5-fold more potent against HDAC1 and HDAC6 than **39d**. Both **39a** and **39d** had identical aliphatic linkers, i.e. four methylenes length. For other aliphatic linkers such as five or six methylenes length linkers, the trend was reversed, i.e. the activities for **39b–39c** were slightly weaker than that for **39e–39f** (i.e. **39b–39c**, IC_50_ ≤ 136 nM for HDAC1/6; **39e–39f**, IC_50_ ≤ 70 nM for HDAC1/6). Overall, the “Cap” group variation had minimal impact on the inhibitory activities of HDAC.

We further investigated the effects of the “ZBG” group on the inhibitory activities of HDAC. The result suggested that target compounds with hydroxamic acid as the “ZBG” group had better HDAC inhibitory potency than those with *o*-phenylenediamine or *o*-aminopyridine as the “ZBG” group. In fact, compounds **31**, **32**, and **41** all have micromole range IC_50_ values indicating weak inhibitory activity against HDAC1 and HDAC6.

**Figure 3.**
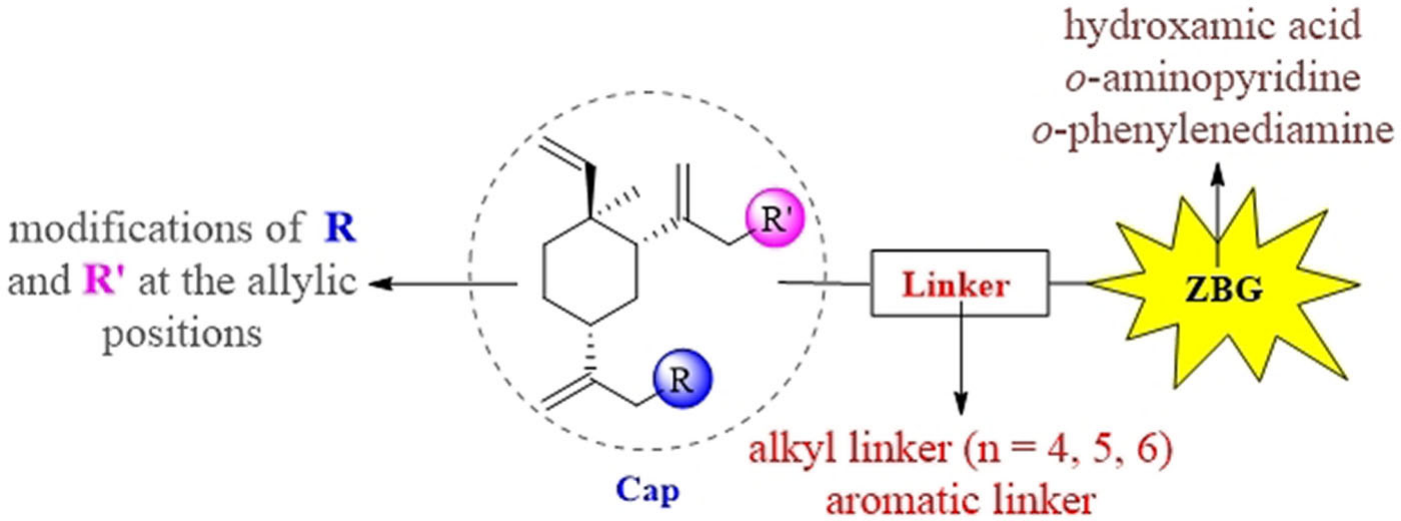
SARs summary of histone deacetylase inhibitors derived from *β*-elemene scaffold.

After the completion of the evaluation for HDAC enzymatic activity of all compounds, SARs were developed and summarised in Figure 3. To sum up, (1) The “Cap” group, bearing different R and R’ group, had no dramatic influence on HDAC inhibitory activities; (2) Proper linker length was significant for retaining HDAC inhibitory activities and the target compounds with linker length five or six methylene groups appeared to be optimal for obtaining potent inhibitory activities against HDAC; (3) The compounds bearing aromatic structures as a part of the linker showed up to 64-fold HDAC6-selectivity over HDAC1; (4) “Hydroxamic acid” appears to be the best “ZBG” group than others such as “*o*-aminopyridine” and the “*o*-phenylenediamine”.

#### *In vitro* anti-proliferative assay

Compounds with good activities against HDAC1 (IC_50_ ≤ 53) and HDAC6 (IC_50_ ≤ 15 nM) were selected to perform anti-proliferative activities experiment against three different leukaemia cells (K562, MV4-11 and HEL cells) using the standard CCK8 test method (Table 2). The results suggested that compounds **18b**, **27c**, **27f** and **39f** were potent in inhibiting the proliferation of three types of leukaemia cells. Among all compounds tested against MV4-11 cell proliferation, **27f** showed the best cytotoxicity with an IC_50_ value of 0.79 µM, which was over 250-fold more potent than *β*-elemene. The IC_50_ values for compounds **18b**, **27c** and **39f** were all less than 1.65 µM. To further identify whether these four molecules had broad-spectrum antitumor activities, we determined their inhibitory activities against two other lymphoma cells: SU-DHL-2 and WSU-DLCL-2 (Table 2). Strikingly, the anti-proliferative activities of compounds **18b**, **27c**, **27f** and **39f** against SU-DHL-2 cell were < 1.55 µM, which was significantly superior to compounds **1** (IC_50_ > 200 µM) and **2** (IC_50_ = 1.84 µM). For WSU-DLCL-2 cells, these four compounds displayed over 88-fold more potent than their parent *β*-elemene. The above results suggested that (1) The anti-proliferative activities of the combination drug were stronger than that of *β*-elemene and SAHA alone, which was consistent with our expectation; (2) Compared with their parent *β*-elemene, our tested compounds showed more potent anti-proliferative activities against five cell lines; (3) The anti-proliferation activities of **27f** were comparable with those of **39f** and slightly stronger those of **18b** and **27c** in all tested tumour cells. Considering the anti-enzyme activities and tumour cell proliferation inhibitory activities, we finally chose compounds **27f** and **39f** as the representative compounds for follow-up research.

**Table 2.** *In vitro* anti-proliferative evaluation of target compounds against selected cell lines.

	IC_50_ (μM)^a^				
	Leukaemia cell lines			Lymphoma cell lines	
Compound	K562	MV4-11	HEL	SU-DHL-2	WSU-DLCL-2
**18b**	5.16 ± 0.13	1.28 ± 0.11	2.16 ± 0.16	1.52 ± 0.13	2.08 ± 0.22
**27c**	5.60 ± 0.04	1.63 ± 0.09	6.05 ± 0.24	1.31 ± 0.18	2.27 ± 0.30
**27f**	4.42 ± 0.56	0.79 ± 0.15	2.43 ± 0.09	1.05 ± 0.09	1.43 ± 0.15
**39f**	3.67 ± 0.50	1.00 ± 0.05	2.01 ± 0.27	1.41 ± 0.04	1.59 ± 0.42
**1**	> 200	> 200	> 200	> 200	> 200
**2**	1.70 ± 0.01	< 0.37	0.60 ± 0.02	1.84 ± 0.21	1.37 ± 0.09
**1 + 2** **(1:1)**	1.61 ± 0.13	< 0.37	0.43 ± 0.15	0.96 ± 0.05	1.19 ± 0.12

^a^Assays were performed in replicate (*n* > 2); IC_50_ values are shown as mean ± SD.

#### The solubility and solubility levels predictions of the selected compounds

Accelrys Discovery Studio (DS) software was used to assess the solubility level of target compounds (Table 3). The results suggested that compounds **18b**, **27c**, **27f** and **39f** were expected to have better aqueous solubility compared to the *β*-elemene group. Compounds **27f** and **39f** are slightly better than **18b** and **27c**.

**Table 3.** The solubility and solubility levels predictions of the selected compounds.

Compound	ADMET solubility	ADMET solubility level
**18b**	−3.342 ↑	3 ↑
**27c**	−3.373 ↑	3 ↑
**27f**	−3.311 ↑	3 ↑
**39f**	−3.273 ↑	3 ↑
**1**	−5.393	2

#### *In vitro* induced cell apoptosis

**Figure 4.**
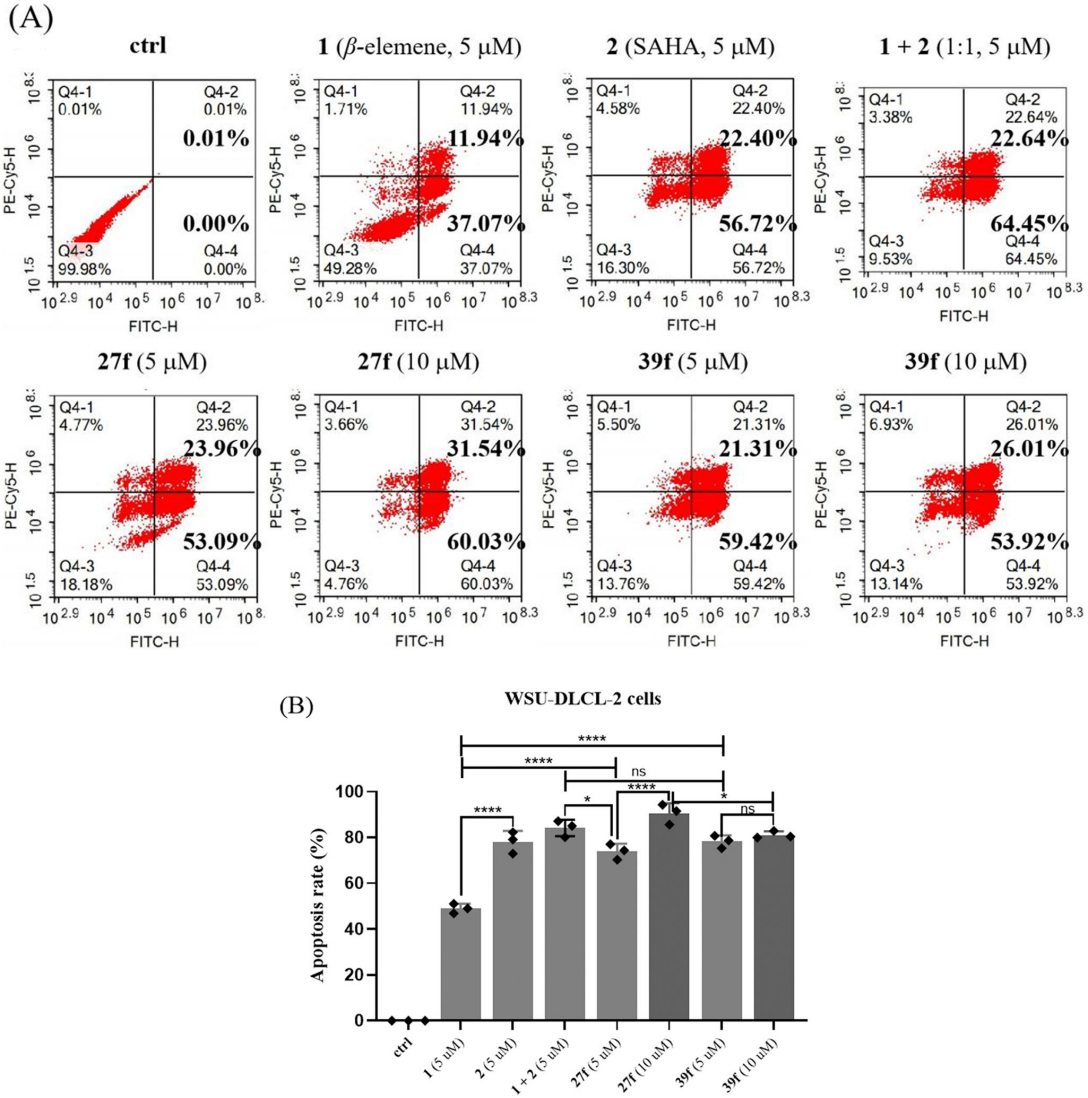
Effects of compounds **27f** and **39f** on cell apoptosis. (A) WSU-DLCL-2 cells were treated with 5 μM and 10 μM of compounds **27f** and **39f** for 72 h. Cells treated with DMSO were used for comparison and cells treated with **1**, **2** and **1 + 2** (1:1) were used for positive control. Data were represented as mean standard deviation from three independent experiments; (B) The histogram of compounds **1**, **2, 1 + 2** (1:1), **27f** and **39f** on WSU-DLCL-2 cells apoptosis. Data are expressed as the mean ± SD (*n* = 3). *p < 0.05, **p < 0.01, ***p < 0.001 by one-way ANOVA test and Turkey’s comparison test, ns: not statistically.

Based on inhibitory activities against HDAC1 and HDAC6, anti-proliferative activities against tumour cells, and calculated aqueous solubility level, compounds **27f** and **39f** were selected for further studies on their pro-apoptotic activity using flow cytometry (Figure 4). Compounds **27f** and **39f** exhibited same magnitude of activity in the five cancer cell lines tested above. Since WSU-DLCL-2 is one of aggressive forms of diffuse large B-cell lympoma (DLBCL) with fewer effective treatment option, it was selected for flow cytometry. The results indicated that the ability of compounds **27f** and **39f** to induce WSU-DLCL-2 cells apoptosis at the concentration of 5 µΜ was comparable to SAHA, but was significantly stronger than *β*-elemene. Interestingly, SAHA possessed a certain level of synergistic pro-apoptotic effects when combined with *β*-elemene at the concentration of 5 µΜ (the percentage of cell apoptosis: 49.01%, 79.12%, and 87.09%, *β*-elemene, SAHA, and *β*-elemene + SAHA (1:1) respectively). Intriguingly, compounds **27f** and **39f** could significantly induced WSU-DLCL-2 cell apoptosis. Compound **27f**, in particular, showed a much higher apoptosis rate at 10 uM than at 5 uM (91.57% *vs* 77.05%) in a dose-dependent manner. The above results indicated that compounds **27f** and **39f** could effectively induce WSU-DLCL-2 cell apoptosis, which are more potent than *β*-elemene.

#### *In vitro* arrested cell cycle

**Figure 5.**
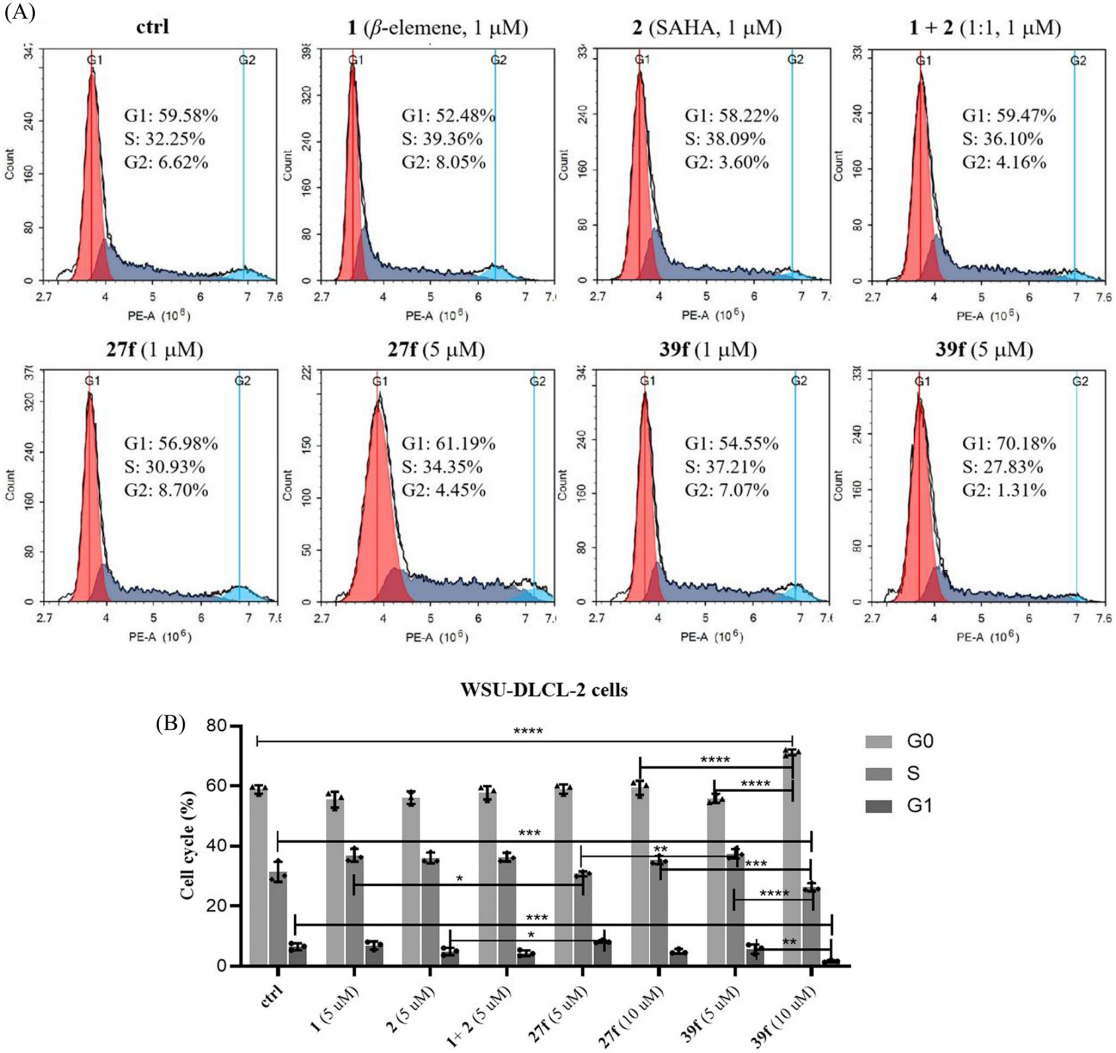
Effects of compounds **27f** and **39f** on cell cycle. (A) WSU-DLCL-2 cells were treated with 1 μM and 5 μM of compounds **27f** and **39f** for 72 h. Cells treated with DMSO were used for comparison and cells treated with **1**, **2** and **1 + 2** (1:1) were used for positive control. Data were represented as mean standard deviation from three independent experiments; (B) The histogram of compounds **1**, **2**, **1 + 2** (1:1), **27f** and **39f** on WSU-DLCL-2 cells cycle. Data are expressed as the mean ± SD (*n* = 3). *p < 0.05, **p < 0.01, ***p < 0.001, ****p < 0.0001 by one-way ANOVA test and Turkey’s comparison test.

In addition to studying the effects of compounds **27f** and **39f** on cell apoptosis, we also explored their effects on cell cycle progression by flow cytometry. As shown in Figure 5, DMSO, *β*-elemene, SAHA and *β*-elemene + SAHA were used as the blank and positive control, respectively. WSU-DLCL-2 cells were treated with compounds **27f** and **39f** at 1 µM and 5 µM for 72 h, respectively. The results revealed that *β*-elemene, SAHA, *β*-elemene + SAHA, **27f** and **39f** groups had hardly or slight effects on cell cycle at 1 µM. When the concentration of compound **27f** was increased to 5 µM, the cell proportion of G1, G2 and S phase did not significantly change, compared with the control group and **27f** group at 1 µM. However, the cell proportion of G1 phase increased dramatically (G1: 54.55% *vs* 70.18%) and the cell proportion of S phase and G2 phase decreased accordingly (S: 37.21% *vs* 27.83%; G2: 7.07% *vs* 1.31%), when the concentration of compound **39f** was increased from 1 µM to 5 µM. The above results indicated that compound 39f could arrest the WSU-DLCL-2 cell cycle at the G1 phase in a concentration-dependent manner.

#### Molecular docking study

**Figure 6.**
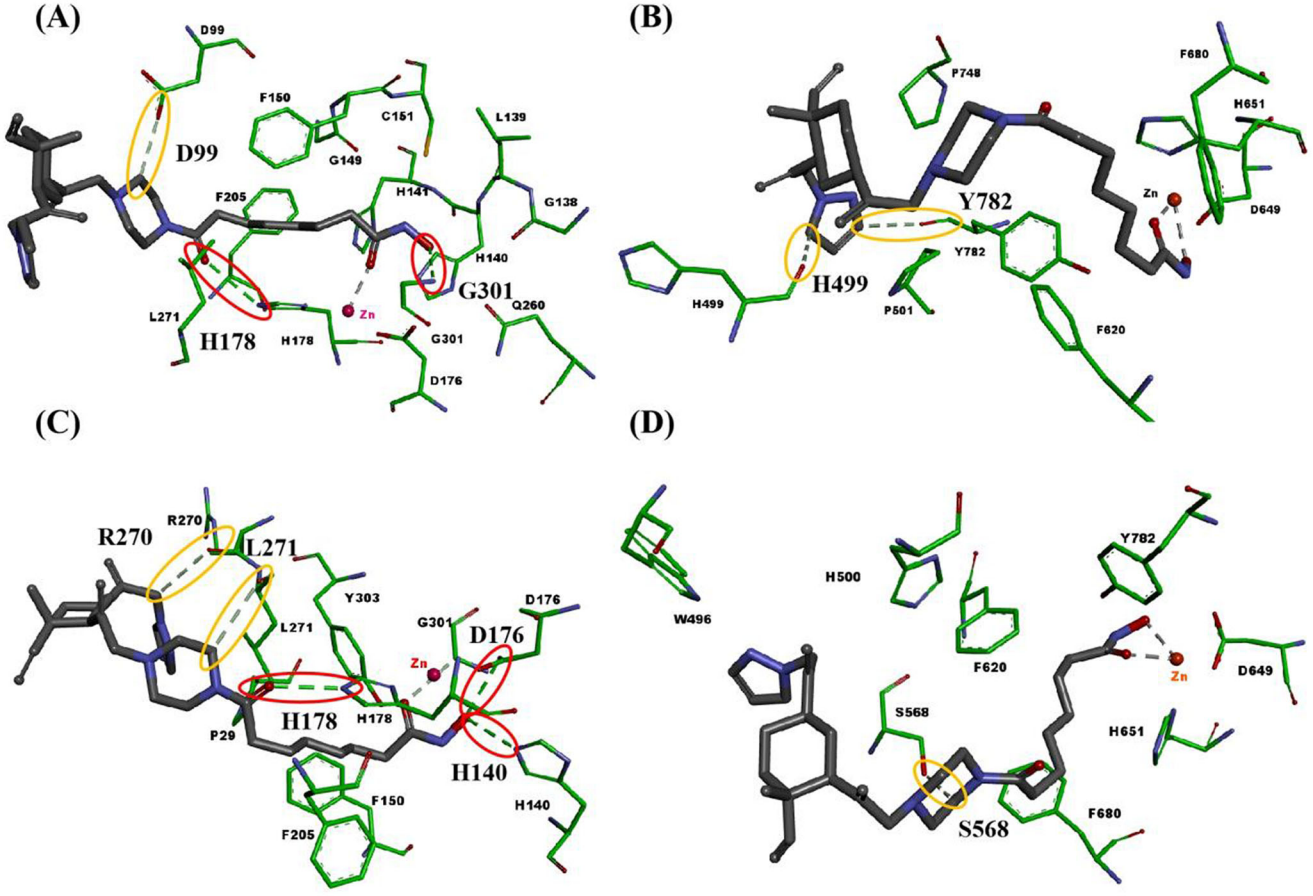
Binding modes of compounds **27f** or **39f** with hHDAC1 (PDB ID 5ICN) and hHDAC6 (PDB ID 5EDU). (A) Binding model of compound **27f** with hHDAC1; (B) Binding model of compound **27f** with hHDAC6; (C) Binding model of compound **39f** with hHDAC1; (D) Binding model of compound **39f** with hHDAC6. Only surrounding amino acid residues are shown for clarity. The zinc ion is representative as red sphere, and hydrogen bonds are indicated with dashed lines. The conventional hydrogen bond is representative as red ellipse. The carbon hydrogen bond is representative as yellow ellipse. The carbons of compounds **27f** and **39f** are coloured in black. The oxygen atoms are coloured in red, and nitrogen atoms in blue.

To better understand the binding modes of compounds **29f** and **39f** with HDAC1, and HDAC6, we performed molecular docking studies using the solved crystal structures of hHDAC1^47^ (PDB ID 5ICN) and hHDAC6^48^ (PDB ID 5EDU), as shown in Figure 6(A–D). Compounds **27f** and **39f** bound with HDAC, mainly through formed metal-acceptor bonding interactions with zinc ions and hydrogen bonds interactions with amino acid residues on the surface of HDAC. In studying the molecular docking of compounds **27f** and **37f** with the active pocket of HDAC1, it was found that both **27f** and **37f** could form metal-acceptor bonding interactions with Zn^2+^. The difference is that **27f** formed three hydrogen bonds with D99, H178 and G301, respectively (Figure 6(A)). In comparison, **39f** formed five hydrogen bonds with R270, L271, H178, D176 and H140, respectively (Figure 6(C)). This may be the reason that compound **27f** had a slightly weaker inhibitory activity against HDAC1 than **39f**. A similar phenomenon was observed in the molecular docking analysis of **27f** and **39f** in the active pocket of HDAC6. The hydroxamic acid moiety of **27f** and **39f** were stretched into the catalytic pocket, forming a coordination contact with Zn^2+^. The difference is that **27f** formed interaction with H499 and Y782 respectively (Figure 6(B)), while **39f** formed interactions with S568 (Figure 6(D)). This may be the reason that compound **27f** displayed more potent inhibitory activity against HDAC6 than **39f**. The docking results proposed the favourable binding mode of our synthesised HDAC inhibitors **27f** and **39f** and explained their good inhibitory activities against HDAC1 and HDAC6.

#### Oral pharmacokinetics study

Due to its excellent HDAC inhibitory activities, potent anti-proliferative and pro-apoptosis capacities and good solubility level, compound **27f** was progressed into an *in vivo* pharmacokinetic (PK) study. Compound **27f** was administered intravenously (*i.v.*) at 5 mg/kg or orally (*p.o.*) at 10 mg/kg to female ICR mice. As shown in Table 4, **27f** was rapidly cleared with the clearance (CL) of 65.1 ml/(h·kg) and the half-life (T_1/2_) of 0.361 h, after intravenous administration. The oral bioavailability of **27f** was low (3.15%), presumably due to the rodent-specific PK profiles of HDACs pharmacophore (similar to SAHA). These data suggested that oral administration was not a suitable dosing route for compound **27f**.

**Table 4.** *In vivo* pharmacokinetic properties of **27f**^a^.

PK parameters	i.v. (5 mg/kg)	p.o. (10 mg/kg)
*T*_1/2_ (h)	0.36 ± 0.10	0.88 ± 0.14
*T*_max_ (h)	－	0.25 ± 0.11
*C*_max_ (ng/ml)	－	54.70 ± 7.20
AUC_0-t_ (h*ng/ml)	1278.00 ± 253.00	77.80 ± 9.30
AUC_0-inf_ (h*ng/ml)	1279.00 ± 142.00	80.70 ± 13.10
Vz (L/kg)	1.08 ± 0.36	158.00 ± 14.00
CL (ml/min/kg)	65.10 ± 11.22	2066.00 ± 394.00
MRT_0-inf_ (h)	0.28 ± 0.08	1.08 ± 0.17
F(%)		3.15 ± 0.05

^a^All experiments are expressed as the mean ± standard deviation of three separate determinations (mean ± SD).

#### *In vivo* antitumor activity against WSU-DLCL2 xenografts

**Figure 7.**
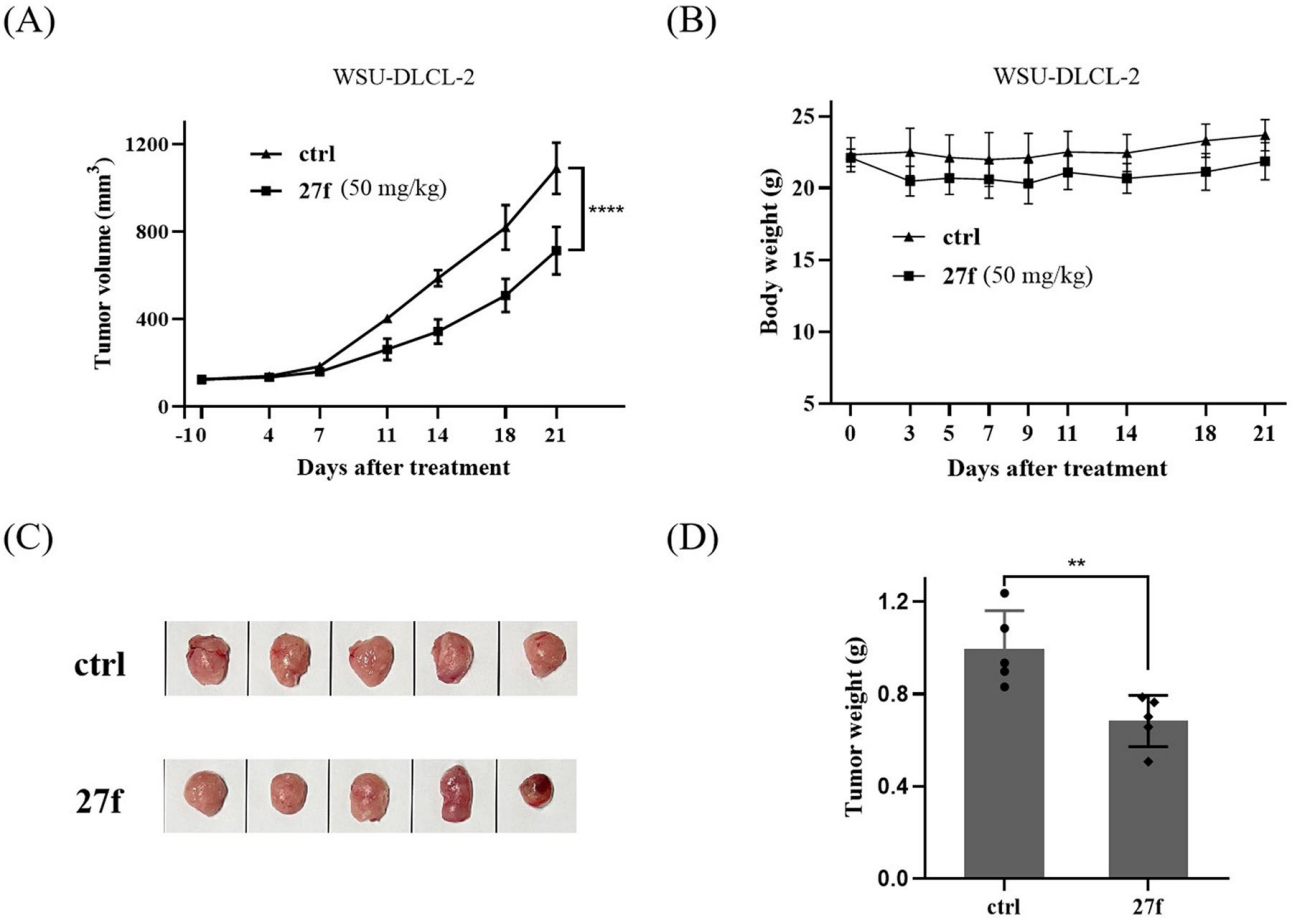
Tumour growth inhibition of compound **27f** in WSU-DLCL-2 xenografted mice model. (A) The efficacy of compound **27f** in the WSU-DLCL-2 xenograft model. (B) Average body weights for **27f** and vehicle-treated mice groups. (C) Photo of dissected WSU-DLCL-2 tumour tissues. (D) Tumour weight of dissected WSU-DLCL-2 tumour tissues. Single asterisks indicate *p* < 0.05, double asterisks indicate *p* < 0.01, and triple asterisks indicate *p* < 0.001 versus the control group. *p < 0.05, **p < 0.01, ***p < 0.001, ****p < 0.0001 by two-way ANOVA test.

In a proof of concept study, **27f** was intraperitoneally administrated to WSU-DLCL-2 xenografted mice model to preliminarily evaluate its *in vivo* antitumor potency. First, WSU-DLCL-2 cells (4 × 10^6^) were implanted in the right flanks subcutaneously in female nude mice. When the implanted tumour reached a volume of 100 ∼ 150 mm^3^, the animals were randomly divided into groups of 2. In one of the groups, animals were treated with compound **27f** intraperitoneally at 50 mg/kg once a day (QD) for 21 consecutive days. In another group, animals were treated with vehicles. As shown in Figure 7, **27f** showed a moderate *in vivo* antitumor potency with TGI of 31.5%. The tumour growth curve and the final tumour tissue size are presented in Figure 7(A,C), respectively, which explicitly demonstrates the superior *in vivo* potency of compound **27f** group.

## Conclusion and discussion

To improve antitumor activities of *β*-elemene and expand its scope of clinical application, we designed and synthesised a novel series of novel HDACi based on *β*-elemene structure in this study. Most of the prepared compounds exhibited potent inhibitory activities against HDACs and significant inhibitory effects on the proliferation of K562 and MV4-11. In particular, the representative compounds **27f** and **39f** demonstrated excellent *in vitro* anti-enzyme (IC_50_ values of 22 nM and 9 nM for HDAC1 and 8 nM and 14 nM for HDAC6, respectively) and broad spectrum *in vitro* anti-proliferative activities (IC_50_ values ranging from 0.79 to 4.42 µM against K562, MV4-11, HEL, SU-DHL-2 and WSU-DLCL-2 cell lines). Preliminary mechanistic studies revealed that compounds **27f** and **39f** could efficiently induce cell apoptosis. Unexpectedly, compound **39f** could also stimulate cell cycle arrest in G1 phase. Further *in vivo* study in WSU-DLCL-2 xenograft mouse model validated the antitumor activities of **27f**, without significant toxicity.

In summary, we have reported a series of novel compounds with potent HDAC inhibitory activity by installing HDACi pharmacophore onto *β*-elemene scaffold. Despite good progress has been obtained, more work is needed to better understand the SAR and to guide the future structural optimisation. For example, it could be interested to evaluate the compounds against other cancer cell lines including both solid tumour cells and haematologic cells. Further optimisation of lead **27f** could also help to identify compounds with improved oral bioavailability. In addition, in-depth mechanism studies should be done with some of the most potent derivatives to elucidate the pathway and target proteins involved in antitumor effects. Nevertheless, our results reported herein should provide useful information for the future studies in this field.

## Experimental section

### Chemistry

#### General information

All of the chemical materials were purchased from commercial suppliers. The melting points of the compounds were determined using Büchi B-540 capillary melting point instrument. NMR spectra were recorded on a Bruker instrument at 400 MHz for ^1^H NMR and 100 MHz for ^13^C NMR, using CDCl_3_, CD_3_OD or DMSO-d_6_ as the deuterated solvent. Chemical shifts (*δ*) were reported in parts per million (ppm) relative to residual solvent as an internal reference. Low-resolution mass spectra were registered with Agilent 1260 InfinityII/6125. High-resolution mass spectra (HRMS) were measured on a Bruker MICR OTOF-Q II instrument.

##### Synthesis of the crucial intermediate 7

To a solution of *β*-elemene (**1**, 6.01 g, 29.46 mmol) in AcOH (20 ml) was added *N*-bromosuccinimide (NBS, 6.29 g, 35.35 mmol) at 0 °C. After addition, the mixture was stirred at room temperature for 9 h. The reaction was monitored by TLC. Upon completion, the mixture was then neutralised with saturated aqueous NaHCO_3_ solution and extracted with petroleum ether (PE, 3 × 100 ml). The combined organic layers were washed with water and brine, and dried over Na_2_SO_4_. The drying agent was filtered off. The filtrate was concentrated under reduced pressure, and the residue was purified *via* flash column chromatography (PE as eluent) to give 13-Br-*β*-elemene **7** (2.51 g, yield 30.1%) as a light yellow liquid. ^1^H NMR (400 MHz, CDCl_3_) *δ* 5.89–5.76 (m, 1H), 5.21 (s, 1H), 5.04 (t, *J* = 1.1 Hz, 1H), 4.97–4.81 (m, 3H), 4.59 (*d*t, *J* = 1.9, 0.9 Hz, 1H), 4.04 (*d*, *J* = 0.7 Hz, 2H), 2.33–2.17 (m, 1H), 2.06 (*dd*, *J* = 12.6, 3.5 Hz, 1H), 1.74–1.70 (m, 3H), 1.69–1.39 (m, 6H), 1.01 (s, 3H).

##### Synthesis of intermediate 10a

A solution of intermediate **7** (1.72 mmol), bis(tert-butoxycarbonyl)amine (**8**, 4.47 mmol) and Cs_2_CO_3_ (2.58 mmol) in *N*,*N*-dimethylformamide (DMF, 5 ml) was stirred at 60 °C for 10 h. The reaction was monitored by TLC. Upon completion, the mixture was diluted in H_2_O (50 ml) and was extracted with ethyl acetate (EA, 3 × 30 ml). The combined organic layers were washed with water and brine, and dried over Na_2_SO_4_. The drying agent was filtered off. The filtrate was concentrated under reduced pressure, and the residue was purified *via* column chromatography (PE/EA 4:1, v/v) to give **10a** (575.93 mg, yield 79.8%) as a light yellow liquid.

##### Synthesis of intermediate 10b

A solution of intermediate **7** (0.40 mmol), 1-Boc-piperazine (0.48 mmol) and DIPEA (0.48 mmol) in DMF (5 ml) was stirred at 60 °C for 10 h. The reaction was monitored by TLC. Upon completion, the mixture was diluted in H_2_O (50 ml) and was extracted with EA (3 × 50 ml). The combined organic layers were washed with water and brine, and dried over Na_2_SO_4_. The drying agent was filtered off. The filtrate was concentrated under reduced pressure, and the residue was purified *via* column chromatography (PE/EA 3:1, v/v) to give **10b** (122.40 mg, yield 78.8%) as a light yellow liquid. ^1^H NMR (400 MHz, CDCl_3_) *δ* 5.76 (dd, *J* = 17.9, 10.4 Hz, 1H), 4.94–4.77 (m, 4H), 4.77–4.72 (m, 1H), 4.51 (s, 1H), 3.35 (s, 4H), 2.86 (q, *J* = 11.6, 9.8 Hz, 2H), 2.26 (s, 4H), 2.08–1.89 (m, 2H), 1.64 (s, 3H), 1.62–1.40 (m, 6H), 1.39 (s, 9H), 0.94 (s, 3H).

##### General procedure for the synthesis of intermediate 11

To a solution of intermediate **10** (1.37 mmol) in dichloromethane (DCM, 3 ml) in an ice-cooled bath was slowly added CF_3_CO_2_H (1.5 ml) solution. The mixture was gradually warmed to room temperature and stirred for 5 h. The reaction was monitored by TLC. Upon completion, the reaction solution was slowly added to the saturated NaHCO_3_ solution (until no bubbles were formed) at 0 °C. Then the mixture was extracted with EA (3 × 50 ml). The combined organic layers were washed with water and brine, and dried over Na_2_SO_4_. The drying agent was filtered off. The filtrate was concentrated under reduced pressure, and the residue was directly used in the next reaction without purification.

##### General procedure for the synthesis of intermediates 13a–c

A solution of H_2_N − OTHP (1.63 mmol), DIPEA (4.08 mmol), EDCI (3.54 mmol), HOBt (1.77 mmol) and the corresponding acids (**12**, 1.36 mmol) in DMF (5 ml) was stirred at room temperature for 5 h. The reaction was monitored by TLC. Upon completion, the mixture was diluted in H_2_O (50 ml) and was extracted with EA (3 × 50 ml). The combined organic layers were washed with water and brine, and dried over Na_2_SO_4_. The drying agent was filtered off. The filtrate was concentrated under reduced pressure, and the residue was purified *via* column chromatography (DCM/MeOH 9:1, v/v) to give the products as a colourless liquid.

###### Methyl 6-oxo-6-(((tetrahydro-2H-pyran-2-yl)oxy)amino)hexanoate (13a)

Colourless liquid, yield 81.0%. ^1^H NMR (500 MHz, CDCl_3_) *δ* 8.60 (s, 1H), 4.95 (s, 1H), 4.04–3.87 (m, 1H), 3.67 (s, 3H), 3.66–3.61 (m, 1H), 2.35 (td, *J* = 6.7, 6.2, 3.4 Hz, 2H), 2.18 (d, *J* = 30.1 Hz, 2H), 1.93–1.47 (m, 10H).

###### Methyl 7-oxo-7-(((tetrahydro-2H-pyran-2-yl)oxy)amino)heptanoate (13b)

Colourless liquid, yield 92.0%. ^1^H NMR (500 MHz, CDCl_3_) *δ* 8.48 (s, 1H), 4.94 (s, 1H), 3.95 (s, 1H), 3.67 (s, 3H), 3.66–3.61 (m, 1H), 2.32 (t, *J* = 7.5 Hz, 2H), 2.13 (s, 2H), 1.90–1.78 (m, 3H), 1.73–1.53 (m, 7H), 1.42–1.32 (m, 2H).

###### Methyl 8-oxo-8-(((tetrahydro-2H-pyran-2-yl)oxy)amino)octanoate (13c)

Colourless liquid, yield 86.4%. ^1^H NMR (400 MHz, CDCl_3_) *δ* 8.35 (s, 1H), 4.95 (s, 1H), 3.93 (d, *J* = 10.6 Hz, 1H), 3.67 (s, 3H), 3.66–3.60 (m, 1H), 2.31 (t, *J* = 7.4 Hz, 2H), 2.11 (m, 2H), 1.90–1.73 (m, 3H), 1.67–1.54 (m, 7H), 1.41–1.29 (m, 4H).

##### General procedure for the synthesis of intermediates 14a–c

A solution of intermediates **13** (1.03 mmol) and cooled NaOH aqueous solution (1 N, 3 ml) in CH_3_OH (3 ml) was stirred at room temperature for 2 h. The reaction was monitored by TLC. Upon completion, the mixture was concentrated *in vacuo*. Then the residue was neutralised by HCl solution (1 M) to pH 6 ∼ 7 and was extracted with EA (3 × 20 ml). The organic layer was separated, washed with water and brine, dried over Na_2_SO_4_. The drying agent was filtered off. The filtrate was concentrated under reduced pressure, and the residue was directly used in the next reaction without purification.

##### Synthesis of intermediate 16

To a solution of H_2_N − OTHP (17.46 mmol) and Et_3_N (31.75 mmol) in DCM (15 ml) was added dropwise DCM solution (15 ml) containing compound **15** (15.87 mmol) at 0 °C. After addition, the mixture was stirred at room temperature for 2 ∼ 3 h. The reaction was monitored by TLC. Upon completion, the mixture was diluted in H_2_O (50 ml) and was extracted with DCM (3 × 50 ml). The organic layer was separated, washed with water and brine, dried over Na_2_SO_4_. The drying agent was filtered off. The filtrate was concentrated under reduced pressure, and the residue was purified *via* column chromatography (PE/EA 3:2, v/v) to give **16** (1.51 g, yield 35.4%) as a white solid. ^1^H NMR (500 MHz, DMSO-d_6_) *δ* 11.66 (s, 1H), 7.77 (d, *J* = 8.2 Hz, 2H), 7.53 (d, *J* = 8.2 Hz, 2H), 5.00 (d, *J* = 3.1 Hz, 1H), 4.81 (s, 2H), 4.05 (t, *J* = 9.7 Hz, 1H), 3.61–3.44 (m, 1H), 1.64 (dtd, *J* = 86.9, 11.2, 9.3, 4.9 Hz, 6H).

##### General procedure for the synthesis of intermediates 17a–f

A solution of **11** (0.70 mmol), DIPEA (2.1 mmol), EDCI (1.82 mmol), HOBt (0.91 mmol) and the corresponding acids **14** (0.84 mmol) in DMF (5 ml) was stirred at room temperature for 5 h. The reaction was monitored by TLC. Upon completion, the mixture was diluted in H_2_O (50 ml) and was extracted with EA (3 × 50 ml). The organic layer was separated, washed with water and brine, dried over Na_2_SO_4_. The drying agent was filtered off. The filtrate was concentrated under reduced pressure, and the residue was purified *via* column chromatography (DCM/MeOH 99:1 ∼ 9:1, v/v) to give the products as a light yellow liquid.

##### General procedure for the synthesis of the target compounds 18a–f

To a solution of **17** (0.45 mmol) in MeOH (3 ml) was added TsOH·H_2_O (0.90 mmol) and stirred at room temperature for 8 h. The reaction was monitored by TLC. Upon completion, the mixture was concentrated *in vacuo*. Then, the residue was diluted in H_2_O (10 ml) and was extracted with DCM (3 × 15 ml). The organic layer was separated, washed with saturated NaHCO_3_ solution and brine, dried over Na_2_SO_4_. The drying agent was filtered off. The filtrate was concentrated under reduced pressure, and the residue was purified *via* C18 column chromatography (MeCN/H_2_O 1:4 ∼ 4:1, v/v) to afford the products.

###### N^1^-hydroxy-N^6^-(2-((1R,3S,4S)-4-methyl-3-(prop-1-en-2-yl)-4-vinylcyclohexyl)allyl)adipamide (18a)

White solid, yield 63.1%, m.p. 95.6–96.7 °C. ^1^H NMR (500 MHz, CDCl_3_) *δ* 6.78 (t, *J* = 5.9 Hz, 1H), 5.80 (dd, *J* = 17.7, 10.5 Hz, 1H), 4.97–4.84 (m, 4H), 4.81 (s, 1H), 4.57 (s, 1H), 3.82 (d, *J* = 5.6 Hz, 2H), 2.22 (d, *J* = 40.7 Hz, 4H), 2.04–1.89 (m, 2H), 1.69 (s, 3H), 1.68–1.52 (m, 7H), 1.52–1.39 (m, 3H), 0.99 (s, 3H). ^13^C NMR (126 MHz, CDCl_3_) *δ* 173.76, 171.43, 150.40, 150.00, 147.41, 112.29, 110.07, 108.18, 52.65, 42.99, 42.69, 39.87, 39.77, 35.87, 33.08, 32.33, 27.21, 24.97, 24.92, 24.87, 16.61. HRMS (ESI) *m/z*: calcd for C_21_H_34_N_2_O_3_ [M + H]^+^, 363.2642; found, 363.2641.

###### N^1^-hydroxy-N^7^-(2-((1R,3S,4S)-4-methyl-3-(prop-1-en-2-yl)-4-vinylcyclohexyl)allyl)heptanediamide (18b)

Colourless viscous colloid, yield 58.9%, m.p. 55.7–58.1 °C. ^1^H NMR (500 MHz, CDCl_3_) *δ* 6.35 (s, 1H), 5.80 (dd, *J* = 17.7, 10.5 Hz, 1H), 4.95–4.87 (m, 3H), 4.86 (s, 1H), 4.82 (t, *J* = 1.7 Hz, 1H), 4.57 (d, *J* = 1.9 Hz, 1H), 3.85 (d, *J* = 5.6 Hz, 2H), 2.29–2.11 (m, 4H), 2.04–1.90 (m, 2H), 1.70 (s, 3H), 1.68–1.53 (m, 5H), 1.52–1.40 (m, 3H), 1.40–1.24 (m, 4H), 0.99 (s, 3H). ^13^C NMR (126 MHz, CDCl_3_) *δ* 173.67, 171.44, 150.52, 149.99, 147.40, 112.27, 110.06, 108.32, 52.67, 43.00, 42.72, 39.85, 39.77, 36.16, 33.11, 32.36, 28.32, 27.20, 25.08, 24.89, 24.84, 16.61. HRMS (ESI) *m/z*: calcd for C_22_H_36_N_2_O_3_ [M + H]^+^, 377.2799; found, 377.2798.

###### N^1^-hydroxy-N^8^-(2-((1R,3S,4S)-4-methyl-3-(prop-1-en-2-yl)-4-vinylcyclohexyl)allyl)octanediamide (18c)

Colourless viscous colloid, yield 72.3%, m.p. 58.6–59.3 °C. ^1^H NMR (500 MHz, CDCl_3_) *δ* 6.04 (s, 1H), 5.80 (dd, *J* = 17.8, 10.5 Hz, 1H), 4.96–4.76 (m, 5H), 4.58 (s, 1H), 3.86 (s, 2H), 2.27–2.09 (m, 4H), 2.02–1.91 (m, 2H), 1.70 (s, 3H), 1.64–1.30 (m, 14H), 1.00 (s, 3H). ^13^C NMR (126 MHz, CDCl_3_) *δ* 173.77, 171.50, 150.55, 150.00, 147.37, 112.28, 110.05, 108.30, 52.68, 42.98, 42.70, 39.86, 39.76, 36.37, 33.10, 29.69, 28.61, 28.40, 27.20, 25.53, 25.17, 24.84. HRMS (ESI) *m/z*: calcd for C_23_H_38_N_2_O_3_ [M + H]^+^, 391.2955; found, 391.2955.

###### N-hydroxy-6–(4-(2-((1R,3S,4S)-4-methyl-3-(prop-1-en-2-yl)-4-vinylcyclohexyl)allyl)piperazin-1-yl)-6-oxohexanamide (18d)

Colourless liquid, yield 70.3%. ^1^H NMR (500 MHz, CDCl_3_) *δ* 8.29 (s, 1H), 5.80 (dd, *J* = 17.8, 10.5 Hz, 1H), 5.12 (d, *J* = 10.0 Hz, 2H), 4.96–4.86 (m, 2H), 4.82 (s, 1H), 4.57 (s, 1H), 3.68 (d, *J* = 46.1 Hz, 4H), 3.28 (d, *J* = 15.4 Hz, 2H), 2.76 (d, *J* = 40.0 Hz, 4H), 2.36 (t, *J* = 6.6 Hz, 2H), 2.20 (t, *J* = 5.9 Hz, 2H), 2.04 (ddd, *J* = 37.4, 12.2, 6.2 Hz, 2H), 1.70 (s, 3H), 1.69–1.35 (m, 10H), 0.99 (s, 3H). ^13^C NMR (126 MHz, CDCl_3_) *δ* 171.96, 166.12, 149.99, 147.48, 147.05, 115.06, 112.21, 110.07, 62.15, 52.58 (d, *J* = 18.0 Hz), 52.05, 44.13, 41.63, 40.26, 39.76 (d, *J* = 2.0 Hz), 33.08, 32.38, 29.69, 27.14, 24.82 (d, *J* = 11.0 Hz), 24.19, 16.55. HRMS (ESI) *m/z*: calcd for C_25_H_41_N_3_O_3_ [M + H]^+^, 432.3221; found, 432.3222.

###### N-hydroxy-7–(4-(2-((1R,3S,4S)-4-methyl-3-(prop-1-en-2-yl)-4-vinylcyclohexyl)allyl)piperazin-1-yl)-7-oxoheptanamide (18e)

White solid, yield 60.7%, m.p. 126.7–127.5 °C. ^1^H NMR (500 MHz, DMSO-d_6_) *δ* 10.32 (s, 1H), 8.65 (s, 1H), 5.82 (dd, *J* = 17.9, 10.5 Hz, 1H), 4.98–4.83 (m, 4H), 4.78 (p, *J* = 1.5 Hz, 1H), 4.58 (d, *J* = 2.2 Hz, 1H), 3.41 (q, *J* = 5.8, 5.3 Hz, 4H), 2.90 (q, *J* = 13.4 Hz, 2H), 2.33–2.22 (m, 6H), 2.11–1.99 (m, 2H), 1.93 (t, *J* = 7.4 Hz, 2H), 1.67 (s, 3H), 1.65–1.55 (m, 2H), 1.53–1.32 (m, 8H), 1.29–1.20 (m, 2H), 0.97 (s, 3H). ^13^C NMR (126 MHz, DMSO-d_6_) *δ* 170.93, 169.50, 150.83, 150.52, 147.63, 112.69, 111.53, 110.44, 63.18, 53.56, 53.07, 52.38, 45.46, 42.06, 41.52, 33.17, 32.63, 28.84, 27.21, 25.45, 25.01 (d, *J* = 5.3 Hz), 16.75. HRMS (ESI) *m/z*: calcd for C_26_H_43_N_3_O_3_ [M + H]^+^, 446.3377; found, 446.3378.

###### N-hydroxy-8–(4-(2-((1R,3S,4S)-4-methyl-3-(prop-1-en-2-yl)-4-vinylcyclohexyl)allyl)piperazin-1-yl)-8-oxooctanamide (18f)

White solid, yield 73.2%, m.p. 144.3–144.9 °C. ^1^H NMR (500 MHz, DMSO-d_6_) *δ* 10.32 (s, 1H), 8.65 (s, 1H), 5.82 (dd, *J* = 17.9, 10.5 Hz, 1H), 4.96–4.82 (m, 4H), 4.78 (p, *J* = 1.5 Hz, 1H), 4.58 (d, *J* = 2.3 Hz, 1H), 3.41 (q, *J* = 5.8 Hz, 4H), 2.90 (q, *J* = 13.4 Hz, 2H), 2.34–2.19 (m, 6H), 2.11–1.98 (m, 2H), 1.93 (t, *J* = 7.4 Hz, 2H), 1.67 (s, 3H), 1.65–1.54 (m, 2H), 1.52–1.31 (m, 8H), 1.30–1.18 (m, *J* = 4.8, 4.0 Hz, 4H), 0.97 (s, 3H). ^13^C NMR (126 MHz, DMSO-d_6_) *δ* 170.98, 169.56, 150.82, 150.53, 147.63, 112.69, 111.54, 110.45, 63.18, 53.58, 53.08, 52.38, 45.48, 42.06, 41.52, 33.17, 32.70, 28.93 (d, *J* = 6.7 Hz), 27.21, 25.50, 25.18, 25.03, 16.75. HRMS (ESI) *m/z*: calcd for C_27_H_45_N_3_O_3_ [M + H]^+^, 460.3534; found, 460.3538.

##### Synthesis of the intermediate 19

A solution of **11b** (0.52 mmol), DIPEA (0.78 mmol) and the benzyl chloride **16** (0.62 mmol) in DMF (4 ml) was stirred at 60 °C for 6 h. The reaction was monitored by TLC. Upon completion, the mixture was diluted in H_2_O (40 ml) and was extracted with EA (3 × 30 ml). The organic layer was separated, washed with water and brine, dried over Na_2_SO_4_. The drying agent was filtered off. The filtrate was concentrated under reduced pressure and the residue was purified *via* flash column chromatography (DCM/MeOH 49:1, v/v) to give **19** (203.48 mg, yield 75.0%) as a colourless liquid. ^1^H NMR (400 MHz, CDCl_3_) *δ* 8.75 (s, 1H), 7.63 (d, *J* = 7.9 Hz, 2H), 7.33 (d, *J* = 7.9 Hz, 2H), 5.75 (dd, *J* = 17.6, 10.7 Hz, 1H), 5.01 (s, 1H), 4.89–4.79 (m, 4H), 4.75 (t, *J* = 1.8 Hz, 1H), 4.54–4.48 (m, 1H), 3.99–3.89 (m, 1H), 3.64–3.55 (m, 1H), 3.47 (s, 2H), 2.91–2.79 (m, 2H), 2.37 (s, 8H), 1.98 (ddd, *J* = 20.6, 11.6, 4.3 Hz, 2H), 1.90–1.75 (m, 3H), 1.71–1.32 (m, 12H), 0.93 (s, 3H).

##### Synthesis of the target compound 20

To a solution of **19** (0.38 mmol) in MeOH (3 ml) was added TsOH·H_2_O (0.76 mmol) and stirred at room temperature for 8 h. The reaction was monitored by TLC. Upon completion, the mixture was concentrated *in vacuo*. Then, the residue was diluted in H_2_O (15 ml) and was extracted with DCM (3 × 15 ml). The organic layer was separated, washed with saturated NaHCO_3_ solution and brine, dried over Na_2_SO_4_, and concentrated *in vacuo*. The residue was purified *via* C18 column chromatography (MeCN/H_2_O 3:2, v/v) to afford **20** (141.35 mg, yield 85.1%) as a white solid, m.p. 99.4–100.2 °C. ^1^H NMR (500 MHz, DMSO-d_6_) *δ* 7.70 (d, *J* = 8.3 Hz, 2H), 7.35 (d, *J* = 8.3 Hz, 2H), 5.82 (dd, *J* = 17.9, 10.5 Hz, 1H), 4.91–4.84 (m, 4H), 4.77 (t, *J* = 1.8 Hz, 1H), 4.57 (d, *J* = 2.2 Hz, 1H), 3.32 (d, *J* = 10.9 Hz, 2H), 2.88 (q, *J* = 13.3 Hz, 2H), 2.36 (s, 8H), 2.08–1.95 (m, 2H), 1.66 (s, 3H), 1.64–1.51 (m, 2H), 1.50–1.39 (m, 3H), 1.36–1.31 (m, 1H), 0.96 (s, 3H). ^13^C NMR (126 MHz, DMSO-d_6_) *δ* 151.13, 150.53, 147.65, 142.07, 131.86, 129.10, 127.23, 112.65, 111.33, 110.42, 63.41, 62.10, 53.24 (d, *J* = 7.1 Hz), 52.40, 42.20, 33.18, 27.17, 25.02, 16.75. HRMS (ESI) *m/z*: calcd for C_27_H_39_N_3_O_2_ [M + H]^+^, 438.3115; found, 438.3117.

##### Synthesis of the crucial intermediate 21

To a solution of *β*-elemene (**1**, 29.36 mmol) and tetrabutylammonium fluoride (TBAF, 1.0 mol/l THF) in DCM and AcOH was stirred at 0 °C. Then, NaClO solution (available chlorine ≥ 5.2%) was slowly added dropwise with a micro-injection pump within 5 h at 0 °C. The reaction was monitored by TLC. Upon completion, the reaction solution was slowly added to the saturated NaHCO_3_ solution (until no bubbles were formed) at 0 °C. Then the mixture was extracted three times with PE (3 × 150 ml). The organic layer was separated, washed with brine, dried over Na_2_SO_4_, and concentrated *in vacuo*. The residue was purified via column chromatography (PE) to afford **21** (2.50 g, yield 31.17%) as a colourless liquid. ^1^H NMR (400 MHz, CDCl_3_) *δ* 5.79 (dd, *J* = 17.2, 11.1 Hz, 1H), 5.28 (s, 1H), 5.18 (s, 1H), 5.04 (s, 1H), 4.99–4.89 (m, 3H), 4.13–4.08 (m, 4H), 3.98 (d, *J* = 11.7 Hz, 1H), 3.78–3.66 (m, 2H), 2.36–2.21 (m, 2H), 1.78–1.40 (m, 10H), 0.99 (s, 3H).

##### Synthesis of intermediate 22a

To a solution of 13,14-Cl-*β*-elemene (**21**, 3.66 mmol) in DMF (10 ml) was added Cs_2_CO_3_ (4.39 mmol) and bis(tert-butoxycarbonyl)amine (4.39 mmol) successively at room temperature. Then mixture was stirred at 50 °C for 8 h. The reaction was monitored by TLC. Upon completion, the mixture was diluted in H_2_O (100 ml) and was extracted with EA (3 × 100 ml). The combined organic layers were washed with water and brine, and dried over Na_2_SO_4_. The drying agent was filtered off. The filtrate was concentrated under reduced pressure and the residue was purified *via* flash column chromatography (PE/EA 1:4, v/v) to give **22a** (1244.71 mg, yield 74.9%) as a light yellow liquid. ^1^H NMR (400 MHz, CDCl_3_) *δ* 5.83–5.73 (m, 1H), 5.26 (d, *J* = 1.0 Hz, 1H), 4.98–4.86 (m, 4H), 4.77 (s, 1H), 4.20 (d, *J* = 1.7 Hz, 2H), 3.96 (dd, *J* = 11.6, 0.8 Hz, 1H), 2.98–2.87 (m, 1H), 2.31–2.20 (m, 1H), 2.00 (dd, *J* = 11.1, 4.0 Hz, 1H), 1.74–1.61 (m, 3H), 1.59–1.50 (m, 2H), 1.48 (s, 18H), 1.46–1.41 (m, 1H), 0.98 (s, 3H).

##### Synthesis of intermediate 22b

To a solution of 13,14-Cl-*β*-elemene (**21**, 1.77 mmol) in DMF (10 ml) was added Cs_2_CO_3_ (2.12 mmol) and 1-Boc-piperazine (2.12 mmol) successively at room temperature. Then mixture was stirred at 50 °C for 8 h. The reaction was monitored by TLC. Upon completion, the mixture was diluted in H_2_O (100 ml) and was extracted with EA (3 × 100 ml). The combined organic layers were washed with water and brine, and dried over Na_2_SO_4_. The drying agent was filtered off. The filtrate was concentrated under reduced pressure and the residue was purified *via* flash column chromatography (PE/EA 1:3, v/v) to give **22b** (558.51 mg,yield 81.2%) as a light yellow liquid. ^1^H NMR (400 MHz, CDCl_3_) *δ* 5.79 (dd, *J* = 17.4, 10.8 Hz, 1H), 5.27 (s, 1H), 4.97–4.86 (m, 5H), 4.10 (dd, *J* = 11.6, 1.1 Hz, 1H), 3.97 (d, *J* = 11.7 Hz, 1H), 3.41 (t, *J* = 4.9 Hz, 4H), 2.98–2.86 (m, 2H), 2.32 (q, *J* = 4.4, 3.8 Hz, 4H), 2.30–2.25 (m, 1H), 2.14 (ddd, *J* = 11.6, 8.9, 6.1 Hz, 1H), 1.69–1.57 (m, 3H), 1.57–1.47 (m, 3H), 1.46 (s, 9H), 0.98 (s, 3H). LCMS *m/z* [M + H]^+^: 423.5.

##### Synthesis of intermediate 24a

To a solution of **22a** (2.70 mmol) in DMF (10 ml) was added Cs_2_CO_3_ (4.05 mmol) and 1*H*-pyrazole (**23**, 3.24 mmol) successively at room temperature. Then mixture was stirred at 60 °C for 10 h. The reaction was monitored by TLC. Upon completion, the mixture was diluted in H_2_O (100 ml) and was extracted with EA (3 × 100 ml). The combined organic layers were washed with water and brine, and dried over Na_2_SO_4_. The drying agent was filtered off. The filtrate was concentrated under reduced pressure and the residue was purified *via* flash column chromatography (PE/EA 3:1, v/v) to give **24a** (993.97 mg, yield 75.8%) as a light yellow liquid. ^1^H NMR (400 MHz, CDCl_3_) *δ* 7.50 (d, *J* = 1.8 Hz, 1H), 7.31 (d, *J* = 2.3 Hz, 1H), 6.25 (t, *J* = 2.1 Hz, 1H), 5.83 (dd, *J* = 17.4, 10.8 Hz, 1H), 5.05–4.96 (m, 2H), 4.85 (d, *J* = 14.9 Hz, 2H), 4.78 (s, 1H), 4.73 (dd, *J* = 8.8, 7.0 Hz, 2H), 4.65 (d, *J* = 15.6 Hz, 1H), 4.13 (d, *J* = 1.9 Hz, 2H), 1.94–1.80 (m, 2H), 1.72–1.49 (m, 6H), 1.47 (s, 18H), 1.02 (s, 3H).

##### Synthesis of intermediate 24b

To a solution of **22b** (2.22 mmol) in DMF (10 ml) was added Cs_2_CO_3_ (3.33 mmol) and 1*H*-pyrazole (**23**, 2.66 mmol) successively at room temperature. Then mixture was stirred at 60 °C for 10 h. The reaction was monitored by TLC. Upon completion, the mixture was diluted in H_2_O (100 ml) and was extracted with EA (3 × 100 ml). The combined organic layers were washed with water and brine, and dried over Na_2_SO_4_. The drying agent was filtered off. The filtrate was concentrated under reduced pressure and the residue was purified *via* flash column chromatography (PE/EA 3:1, v/v) to give **24b** (789.31 mg, yield 78.2%) as a light yellow liquid. ^1^H NMR (500 MHz, CDCl_3_) *δ* 7.50 (d, *J* = 1.9 Hz, 1H), 7.33 (d, *J* = 2.2 Hz, 1H), 6.25 (t, *J* = 2.1 Hz, 1H), 5.84 (dd, *J* = 17.4, 10.8 Hz, 1H), 5.04–4.96 (m, 2H), 4.95–4.88 (m, 2H), 4.87 (s, 1H), 4.78 (s, 1H), 4.71 (d, *J* = 1.4 Hz, 1H), 4.66 (d, *J* = 15.7 Hz, 1H), 3.39 (s, 4H), 2.88 (q, *J* = 13.5 Hz, 2H), 2.36–2.25 (m, 4H), 2.00 (m, 1H), 1.93 (dd, *J* = 12.7, 3.4 Hz, 1H), 1.60–1.48 (m, 3H), 1.47 − 1.36 (m, 13H), 1.03 (s, 3H). LCMS *m/z* [M + H]^+^: 455.5.

##### General procedure for the synthesis of intermediates 25a–b

To a solution of **24** (1.74 mmol) in MeOH (1.5 ml) in an ice-cooled bath was added a solution of hydrochloride in dioxane (4 M, 6 ml). The mixture was gradually warmed to room temperature and stirred for 8 h. The reaction was monitored by TLC. Upon completion, the solvent was removed under reduced pressure to give the intermediates **25** (1.74 mmol, yield 100%) as a white solid. The residue was used for the next reaction without further purification.

##### General procedure for the synthesis of intermediates 26a–f

A solution of **25** (0.52 mmol), DIPEA (1.56 mmol), EDCI (1.35 mmol), HOBt (0.68 mmol) and the corresponding acids (**14**, 0.62 mmol) in DMF (5 ml) was stirred at room temperature for 5 h. The reaction was monitored by TLC. Upon completion, the mixture was diluted in H_2_O (50 ml) and was extracted with EA (3 × 50 ml). The organic layer was separated, washed with water and brine, dried over Na_2_SO_4_. The drying agent was filtered off. The filtrate was concentrated under reduced pressure, and the residue was purified *via* column chromatography (DCM/MeOH 99:1 ∼ 9:1, v/v) to give the products as a light yellow liquid.

##### General procedure for the synthesis of the target compounds 27a–f

To a solution of **26** (0.41 mmol) in MeOH (3 ml) was added TsOH·H_2_O (0.82 mmol) and stirred at room temperature for 8 h. The reaction was monitored by TLC. Upon completion, the mixture was concentrated *in vacuo*. Then, the residue was diluted in H_2_O (10 ml) and was extracted with DCM (3 × 10 ml). The organic layer was separated, washed with saturated NaHCO_3_ solution and brine, dried over Na_2_SO_4_. The drying agent was filtered off. The filtrate was concentrated under reduced pressure, and the residue was purified *via* C18 column chromatography (MeCN/H_2_O 1:4 ∼ 4:1, v/v) to afford the products.

###### N^1^-(2-((1R,3R,4S)-3–(3-(1H-pyrazol-1-yl)prop-1-en-2-yl)-4-methyl-4-vinylcyclohexyl)allyl)-N^6^-hydroxyadipamide (27a)

White solid, yield 50.6%, m.p. 65.6–66.8 °C. ^1^H NMR (500 MHz, CDCl_3_) *δ* 7.48 (d, *J* = 1.8 Hz, 1H), 7.33 (d, *J* = 2.3 Hz, 1H), 6.25 (t, *J* = 2.1 Hz, 1H), 5.80 (dd, *J* = 17.4, 10.8 Hz, 1H), 5.04–4.95 (m, 2H), 4.84 (t, *J* = 5.9 Hz, 3H), 4.67 (d, *J* = 5.0 Hz, 3H), 3.79 (d, *J* = 5.3 Hz, 2H), 2.19 (d, *J* = 40.0 Hz, 4H), 1.96–1.80 (m, 2H), 1.73–1.36 (m, 10H), 1.00 (s, 3H). ^13^C NMR (126 MHz, CDCl_3_) *δ* 173.54, 150.18, 149.34, 147.43, 139.13, 129.85, 113.93, 111.41, 108.73, 105.73, 58.73, 48.08, 42.99, 42.30, 39.81, 39.65, 35.87, 33.33, 26.88, 25.02 (d, *J* = 7.6 Hz), 15.81. HRMS (ESI) *m/z*: calcd for C_24_H_36_N_4_O_3_ [M + H]^+^, 429.2860; found, 429.2858.

###### N^1^-(2-((1R,3R,4S)-3–(3-(1H-pyrazol-1-yl)prop-1-en-2-yl)-4-methyl-4-vinylcyclohexyl)allyl)-N^7^-hydroxyheptanediamide (27b)

Colourless viscous colloid, yield 42.8%, m.p. 47.3–49.0 °C. ^1^H NMR (500 MHz, CDCl_3_) *δ* 7.48 (s, 1H), 7.33 (d, *J* = 2.3 Hz, 1H), 6.25 (t, *J* = 2.0 Hz, 1H), 5.80 (dd, *J* = 17.4, 10.8 Hz, 1H), 5.10–4.94 (m, 2H), 4.85 (t, *J* = 4.1 Hz, 3H), 4.78–4.61 (m, 3H), 3.80 (s, 2H), 2.32–2.06 (m, 4H), 1.97–1.81 (m, 2H), 1.76–1.18 (m, 12H), 1.01 (s, 3H). ^13^C NMR (126 MHz, CDCl_3_) *δ* 173.52, 150.27, 149.31, 147.39, 139.14, 129.76, 114.13, 111.43, 108.78, 105.74, 58.81, 48.07, 42.94, 42.38, 39.82, 39.65, 36.12, 33.32, 29.70, 26.90, 25.07 (d, *J* = 23.7 Hz), 15.82. HRMS (ESI) *m/z*: calcd for C_25_H_38_N_4_O_3_ [M + H]^+^, 443.3017; found, 443.3021.

###### N^1^-(2-((1R,3R,4S)-3–(3-(1H-pyrazol-1-yl)prop-1-en-2-yl)-4-methyl-4-vinylcyclohexyl)allyl)-N^8^-hydroxyoctanediamide (27c)

Light yellow solid, yield 53.5%, m.p. 54.7–55.9 °C. ^1^H NMR (500 MHz, CDCl_3_) *δ* 7.48 (d, *J* = 2.0 Hz, 1H), 7.33 (d, *J* = 1.8 Hz, 1H), 6.25 (t, *J* = 2.1 Hz, 1H), 5.80 (dd, *J* = 17.4, 10.8 Hz, 1H), 5.04–4.95 (m, 2H), 4.85 (d, *J* = 5.9 Hz, 3H), 4.67 (dd, *J* = 28.0, 15.6 Hz, 3H), 3.81 (s, 2H), 2.28–2.03 (m, 4H), 1.95–1.81 (m, 2H), 1.72–1.16 (m, 14H), 1.01 (s, 3H). ^13^C NMR (126 MHz, CDCl_3_) *δ* 173.64, 150.26, 149.30, 147.36, 139.10, 129.71, 114.20, 111.44, 108.63, 105.74, 58.82, 48.04, 42.91, 42.35, 39.81, 39.65, 36.29, 33.31, 29.69, 28.55 (d, *J* = 17.1 Hz), 26.89, 25.52, 25.27, 15.81. HRMS (ESI) *m/z*: calcd for C_26_H_40_N_4_O_3_ [M + H]^+^, 457.3173; found, 457.3172.

###### 6–(4-(2-((1R,3R,4S)-3–(3-(1H-pyrazol-1-yl)prop-1-en-2-yl)-4-methyl-4-vinylcyclohexyl)allyl)piperazin-1-yl)-N-hydroxy-6-oxohexanamide (27d)

Light yellow viscous colloid, yield 58.9%, m.p. 71.7–72.2 °C. ^1^H NMR (500 MHz, CDCl_3_) *δ* 7.50 (d, *J* = 1.8 Hz, 1H), 7.34 (d, *J* = 2.2 Hz, 1H), 6.25 (t, *J* = 2.1 Hz, 1H), 5.83 (dd, *J* = 17.4, 10.8 Hz, 1H), 5.10–4.83 (m, 5H), 4.81–4.62 (m, 3H), 3.57 (s, 2H), 3.43 (s, 2H), 2.89 (q, *J* = 13.4 Hz, 2H), 2.47–2.13 (m, 8H), 2.07–1.89 (m, 2H), 1.80–1.40 (m, 10H), 1.03 (s, 3H). ^13^C NMR (126 MHz, CDCl_3_) *δ* 171.66, 149.99, 149.46, 147.60, 139.12, 129.49, 114.20, 111.37 (d, *J* = 22.0 Hz), 105.60, 63.26, 59.01, 53.17, 52.81, 48.09, 45.64, 41.83 (d, *J* = 7.3 Hz), 39.90, 39.70, 33.66, 32.61, 29.69, 26.92, 24.89, 24.34, 15.82. HRMS (ESI) *m/z*: calcd for C_28_H_43_N_5_O_3_ [M + H]^+^, 498.3439; found, 498.3436.

###### 7–(4-(2-((1R,3R,4S)-3–(3-(1H-pyrazol-1-yl)prop-1-en-2-yl)-4-methyl-4-vinylcyclohexyl)allyl)piperazin-1-yl)-N-hydroxy-7-oxoheptanamide (27e)

Colourless viscous colloid, yield 51.5%, m.p. 55.3–56.7 °C. ^1^H NMR (500 MHz, CDCl_3_) *δ* 7.50 (d, *J* = 1.9 Hz, 1H), 7.34 (d, *J* = 2.2 Hz, 1H), 6.25 (t, *J* = 2.1 Hz, 1H), 5.83 (dd, *J* = 17.4, 10.8 Hz, 1H), 5.07–4.84 (m, 5H), 4.80–4.63 (m, 3H), 3.65–3.36 (m, 4H), 2.90 (q, *J* = 13.4 Hz, 2H), 2.48–2.09 (m, 8H), 2.04–1.90 (m, 2H), 1.75–1.30 (m, 12H), 1.03 (s, 3H). ^13^C NMR (126 MHz, CDCl_3_) *δ* 171.78, 171.14, 149.97, 149.46, 147.59, 139.12, 129.51, 114.20, 111.49, 111.28, 105.60, 63.24, 59.02, 53.18, 52.81, 48.09, 45.67, 41.85, 39.90, 39.69, 33.67, 32.77, 32.33, 28.44, 26.90, 25.02, 24.47, 15.82. HRMS (ESI) *m/z*: calcd for C_29_H_45_N_5_O_3_ [M + H]^+^, 512.3595; found, 512.3597.

###### 7–(4-(2-((1R,3R,4S)-3–(3-(1H-pyrazol-1-yl)prop-1-en-2-yl)-4-methyl-4-vinylcyclohexyl)allyl)piperazin-1-yl)-N-hydroxy-7-oxoheptanamide (27f)

Colourless viscous colloid, yield 68.7%, m.p. 59.6–60.0 °C. ^1^H NMR (400 MHz, DMSO-d_6_) *δ* 10.34 (s, 1H), 8.69 (s, 1H), 7.60 (d, *J* = 2.2 Hz, 1H), 7.42 (d, *J* = 1.8 Hz, 1H), 6.24 (t, *J* = 2.1 Hz, 1H), 5.87 (dd, *J* = 17.5, 10.8 Hz, 1H), 5.02–4.88 (m, 4H), 4.81 (s, 1H), 4.64 (s, 3H), 3.40 (d, *J* = 15.5 Hz, 4H), 2.86 (s, 2H), 2.24 (dt, *J* = 18.1, 6.1 Hz, 6H), 1.94 (dt, *J* = 14.7, 5.4 Hz, 4H), 1.68–1.32 (m, 10H), 1.32–1.17 (m, *J* = 4.7, 3.5 Hz, 4H), 0.99 (s, 3H). ^13^C NMR (126 MHz, CDCl_3_) *δ* 172.00, 171.16, 149.38, 148.88, 147.58, 139.09, 129.68, 113.97, 112.75, 111.32, 105.61, 62.90, 58.88, 53.10, 52.58, 48.12, 45.22, 41.69, 41.16, 39.85, 39.63, 33.60, 32.90, 32.60, 31.91, 30.30, 29.66 (d, *J* = 4.5 Hz), 28.50 (d, *J* = 20.2 Hz), 28.42, 26.83, 25.01 (d, *J* = 23.7 Hz), 15.82. HRMS (ESI) *m/z*: calcd for C_30_H_47_N_5_O_3_ [M + H]^+^, 526.3752; found, 526.3755.

##### Synthesis of intermediate 28

(i) A solution of **25b** (1.00 mmol), DIPEA (3.00 mmol), EDCI (2.60 mmol), HOBt (1.30 mmol) and the acid **12c** (1.20 mmol) in DMF (6 ml) was stirred at room temperature for 5 h. The reaction was monitored by TLC. Upon completion, the mixture was diluted in H_2_O (60 ml) and was extracted with EA (3 × 50 ml). The organic layer was separated, washed with water and brine, dried over Na_2_SO_4_. The drying agent was filtered off. The filtrate was concentrated under reduced pressure, and the residue was purified *via* column chromatography (DCM/MeOH 19:1, v/v) to give the ester (384.13 mg, yield 73.2%) as a light yellow liquid. ^1^H NMR (500 MHz, CDCl_3_) *δ* 7.43 (d, *J* = 2.0 Hz, 1H), 7.27 (d, *J* = 2.2 Hz, 1H), 6.18 (t, *J* = 2.1 Hz, 1H), 5.77 (dd, *J* = 17.5, 10.8 Hz, 1H), 5.02–4.78 (m, 5H), 4.66 (dd, *J* = 32.9, 17.4 Hz, 3H), 3.59 (s, 3H), 3.51 (s, 2H), 3.35 (s, 2H), 2.91–2.73 (m, 2H), 2.33–2.16 (m, 8H), 2.00–1.78 (m, 2H), 1.66–1.33 (m, 14H), 0.96 (s, 3H).

(ii) A solution of the ester (0.73 mmol) and cooled NaOH aqueous solution (1 N, 3 ml) in MeOH (3 ml) was stirred at room temperature for 2 h. The reaction was monitored by TLC. Upon completion, the mixture was concentrated in vacuo. Then, the residue was neutralised by HCl solution (1 M) to pH 6 ∼ 7 and was extracted with EA (3 × 20ml). The organic layer was separated, washed with water and brine, dried over Na_2_SO_4_. The drying agent was filtered off. The filtrate was concentrated under reduced pressure, and the resulted crude product (**28**, yield 90.6%) as a light yellow liquid, was directly used in the next reaction without purification.

##### Synthesis of the target compound 31

A solution of **28** (0.33 mmol), DIPEA (0.99 mmol), EDCI (0.86 mmol), HOBt (0.43 mmol) and *o*-phenylenediamine **29** (1.65 mmol) in DMF (6 ml) was stirred at room temperature for 5 h. The reaction was monitored by TLC. Upon completion, the mixture was diluted in H_2_O (60 ml) and was extracted with EA (3 × 50 ml). The organic layer was separated, washed with water and brine, dried over Na_2_SO_4_. The drying agent was filtered off. The filtrate was concentrated under reduced pressure and the residue was purified *via* column chromatography (DCM/MeOH 19:1, v/v) to give the target compound **31** (90.61 mg, yield 45.7%) as a colourless viscous colloid, m.p. 59.7–60.5 °C. ^1^H NMR (500 MHz, CDCl_3_) *δ* 7.87 (s, 1H), 7.43 (d, *J* = 1.9 Hz, 1H), 7.26 (d, *J* = 2.3 Hz, 1H), 7.10 (dd, *J* = 7.8, 1.5 Hz, 1H), 6.95 (td, *J* = 7.6, 1.5 Hz, 1H), 6.73–6.64 (m, 2H), 6.18 (t, *J* = 2.1 Hz, 1H), 5.76 (dd, *J* = 17.5, 10.8 Hz, 1H), 5.00–4.77 (m, 5H), 4.73–4.54 (m, 3H), 3.50 (t, *J* = 5.0 Hz, 2H), 3.34 (t, *J* = 5.0 Hz, 2H), 2.89–2.73 (m, 2H), 2.37–2.13 (m, 8H), 2.00–1.80 (m, 2H), 1.68–1.27 (m, 14H), 0.96 (s, 3H). ^13^C NMR (126 MHz, CDCl_3_) *δ* 171.06, 170.57, 148.99, 148.45, 146.60, 139.84, 138.12, 128.40, 125.94, 124.26, 123.46, 118.25, 117.01, 113.23, 110.45, 110.26, 104.56, 62.23, 58.05, 52.22, 51.85, 47.06, 44.65, 40.87, 40.61, 38.88, 38.68, 35.63, 32.64, 32.04, 28.67, 27.77 (d, *J* = 13.2 Hz), 25.88, 24.55, 23.96, 14.79. HRMS (ESI) *m/z*: calcd for C_35_H_50_N_6_O_2_ [M + H]^+^, 587.4068; found, 587.4066.

##### Synthesis of the target compound 32

A solution of **28** (0.31 mmol), DIPEA (0.93 mmol), EDCI (0.81 mmol), HOBt (0.40 mmol) and *o*-aminopyridine **30** (3.10 mmol) in DMF (5 ml) was stirred at room temperature for 5 h. The reaction was monitored by TLC. Upon completion, the mixture was diluted in H_2_O (50 ml) and was extracted with EA (3 × 50 ml). The combined organic layers were washed with water and brine, and dried over Na_2_SO_4_. The drying agent was filtered off. The filtrate was concentrated under reduced pressure and the residue was purified *via* column chromatography (DCM/MeOH 97:3, v/v) to give the target compound **32** (91.14 mg, yield 50.1%) as a brown viscous colloid, m.p. 62.3–63.7 °C. ^1^H NMR (500 MHz, CDCl_3_) *δ* 8.62 (s, 1H), 8.27–8.16 (m, 2H), 7.67 (ddd, *J* = 8.8, 7.4, 1.9 Hz, 1H), 7.47 (d, *J* = 1.9 Hz, 1H), 7.31 (d, *J* = 2.1 Hz, 1H), 7.00 (ddd, *J* = 7.4, 4.9, 1.0 Hz, 1H), 6.22 (t, *J* = 2.1 Hz, 1H), 5.81 (dd, *J* = 17.4, 10.8 Hz, 1H), 5.04–4.93 (m, 2H), 4.92–4.82 (m, 3H), 4.75 (s, 1H), 4.73–4.61 (m, 2H), 3.56 (t, *J* = 5.0 Hz, 2H), 3.39 (t, *J* = 5.0 Hz, 2H), 2.86 (q, *J* = 13.4 Hz, 2H), 2.40–2.24 (m, 8H), 2.03–1.88 (m, 2H), 1.73–1.55 (m, 6H), 1.49–1.33 (m, 8H), 1.00 (s, 3H). ^13^C NMR (126 MHz, CDCl_3_) *δ* 171.92, 171.47, 151.62, 150.07, 149.46, 147.59 (d, *J* = 4.8 Hz), 139.12, 138.43, 129.36, 119.59, 114.20 (d, *J* = 13.5 Hz), 111.31 (d, *J* = 15.2 Hz), 105.56, 63.28, 59.07, 53.26, 52.89, 48.05, 45.66, 41.87, 41.60, 39.89, 39.69, 37.51, 33.66, 33.14, 29.68, 29.10, 28.90, 26.89, 25.12 (d, *J* = 7.7 Hz), 15.80. HRMS (ESI) *m/z*: calcd for C_36_H_52_N_6_O_2_ [M + H]^+^, 601.4225; found, 601.4227.

##### Synthesis of intermediate 33

A solution of intermediate **25b** (0.34 mmol), DIPEA (0.51 mmol) and the benzyl chloride **16** (0.41 mmol) in DMF (2.5 ml) was stirred at 60 °C for 6 h. The reaction was monitored by TLC. Upon completion, the mixture was diluted in H_2_O (25 ml) and was extracted with EA (3 × 25 ml). The combined organic layers were washed with water and brine, and dried over Na_2_SO_4_. The drying agent was filtered off. The filtrate was concentrated under reduced pressure and the residue was purified *via* flash column chromatography (DCM/MeOH 19:1, v/v) to give **33** (131.80 mg, yield 65.9%) as a light yellow liquid. ^1^H NMR (400 MHz, CDCl_3_) *δ* 9.00 (s, 1H), 7.71 (d, *J* = 7.9 Hz, 2H), 7.51 (d, *J* = 1.8 Hz, 1H), 7.39 (d, *J* = 7.9 Hz, 2H), 7.33 (d, *J* = 2.3 Hz, 1H), 6.25 (t, *J* = 2.1 Hz, 1H), 5.83 (dd, *J* = 17.4, 10.8 Hz, 1H), 5.08 (d, *J* = 3.7 Hz, 1H), 5.04–4.95 (m, 2H), 4.93–4.89 (m, 1H), 4.86 (d, *J* = 2.5 Hz, 2H), 4.77–4.60 (m, 3H), 4.06–3.96 (m, 1H), 3.69–3.61 (m, 1H), 3.55 (s, 2H), 2.94–2.82 (m, 2H), 2.42 (s, 8H), 2.10–1.83 (m, 2H), 1.81–1.37 (m, 12H), 1.02 (s, 3H).

##### Synthesis of the target compound 34

To a solution of **33** (0.22 mmol) in MeOH (2 ml) was added TsOH·H_2_O (0.44 mmol) and stirred at room temperature for 8 h. The reaction was monitored by TLC. Upon completion, the mixture was concentrated *in vacuo*. Then, the residue was diluted in H_2_O (10 ml) and was extracted with DCM (3 × 10 ml). The organic layer was separated, washed with saturated NaHCO_3_ solution and brine, dried over Na_2_SO_4_. The drying agent was filtered off. The filtrate was concentrated under reduced pressure and the residue was purified *via* C18 column chromatography (MeCN/H_2_O 3:2, v/v) to afford the target compound **34** (74.58 mg, yield 67.3%) as a white solid, m.p. 111.8–112.6 °C. ^1^H NMR (500 MHz, CDCl_3_) *δ* 7.66 (d, *J* = 7.7 Hz, 2H), 7.48 (d, *J* = 1.6 Hz, 1H), 7.34–7.28 (m, 3H), 6.24 (t, *J* = 2.1 Hz, 1H), 5.79 (dd, *J* = 17.4, 10.8 Hz, 1H), 5.02–4.81 (m, 5H), 4.76–4.60 (m, 3H), 3.52 (s, 2H), 2.88 (q, *J* = 13.4 Hz, 2H), 2.46 (s, 8H), 2.04–1.87 (m, 2H), 1.63–1.36 (m, 6H), 1.00 (s, 3H). ^13^C NMR (126 MHz, CDCl_3_) *δ* 165.94, 150.16, 149.53, 147.65, 141.44, 139.12, 130.56, 129.55 (d, *J* = 5.5 Hz), 127.02, 113.98, 111.29 (d, *J* = 21.2 Hz), 105.62, 63.25, 62.44, 58.88, 52.85 (d, *J* = 16.7 Hz), 48.18, 41.95, 39.79 (d, *J* = 23.2 Hz), 33.64, 26.90, 15.81. HRMS (ESI) *m/z*: calcd for C_30_H_41_N_5_O_2_ [M + H]^+^, 504.3333; found, 504.3333.

##### Synthesis of intermediate 35

To a solution of 13,14-Br-*β*-elemene (**21**, 4.93 mmol) in DMF (10 ml) was added Cs_2_CO_3_ (5.92 mmol) and 1*H*-pyrazole (**23**, 5.92 mmol) successively at room temperature. Then the mixture was stirred at 45 °C for 10 h. The reaction was monitored by TLC. Upon completion, the mixture was diluted in H_2_O (100 ml) and was extracted with EA (3 × 100 ml). The combined organic layers were washed with water and brine, and dried over Na_2_SO_4_. The drying agent was filtered off. The filtrate was concentrated under reduced pressure and the residue was purified *via* flash column chromatography (PE/EA 4:1, v/v) to give **35** (1037.18 mg, yield 69.3%) as a light yellow liquid. ^1^H NMR (500 MHz, CDCl_3_) *δ* 7.53 (d, *J* = 1.6 Hz, 1H), 7.40 (d, *J* = 2.2 Hz, 1H), 6.28 (t, *J* = 2.1 Hz, 1H), 5.75 (dd, *J* = 17.3, 10.9 Hz, 1H), 5.26 (s, 1H), 5.02 (d, *J* = 1.2 Hz, 1H), 4.96–4.85 (m, 3H), 4.78 (s, 3H), 4.06 (dd, *J* = 11.7, 1.1 Hz, 1H), 3.95 (dd, *J* = 11.6, 0.8 Hz, 1H), 2.22 (dd, *J* = 12.5, 3.7 Hz, 1H), 1.95 (tt, *J* = 11.6, 3.7 Hz, 1H), 1.68–1.38 (m, 6H), 0.96 (s, 3H).

##### Synthesis of intermediate 36a

To a solution of intermediate **35** (2.89 mmol) in DMF (10 ml) was added Cs_2_CO_3_ (3.47 mmol) and bis(tert-butoxycarbonyl)amine (3.47 mmol) successively at room temperature. Then the mixture was stirred at 80 °C for 8 h. The reaction was monitored by TLC. Upon completion, the mixture was diluted in H_2_O (100 ml) and was extracted with EA (3 × 100 ml). The combined organic layers were washed with water and brine, and dried over Na_2_SO_4_. The drying agent was filtered off. The filtrate was concentrated under reduced pressure and the residue was purified *via* flash column chromatography (PE/EA 7:3, v/v) to give **36a** (1056.24 mg, yield 75.5%) as a light yellow liquid. ^1^H NMR (400 MHz, CDCl_3_) *δ* 7.51 (d, *J* = 1.8 Hz, 1H), 7.39 (d, *J* = 2.2 Hz, 1H), 6.27 (t, *J* = 2.1 Hz, 1H), 5.85–5.76 (m, 1H), 5.01 (s, 1H), 4.94 (q, *J* = 1.3 Hz, 1H), 4.92–4.89 (m, 1H), 4.86 (d, *J* = 1.8 Hz, 1H), 4.74 (d, *J* = 7.0 Hz, 4H), 4.22 (dt, *J* = 17.0, 2.0 Hz, 1H), 3.89 (d, *J* = 17.0 Hz, 1H), 1.94–1.83 (m, 2H), 1.72–1.53 (m, 3H), 1.48 (s, 3H), 1.47 (s, 18H), 0.99 (s, 3H).

##### Synthesis of intermediate 36b

To a solution of intermediate **35** (1.98 mmol) in DMF (7 ml) was added Cs_2_CO_3_ (2.38 mmol) and 1-Boc-piperazine (2.38 mmol) successively at room temperature. Then the mixture was stirred at 80 °C for 8 h. The reaction was monitored by TLC. Upon completion, the mixture was diluted in H_2_O (70 ml) and was extracted with EA (3 × 70 ml). The combined organic layers were washed with water and brine, and dried over Na_2_SO_4_. The drying agent was filtered off. The filtrate was concentrated under reduced pressure and the residue was purified *via* flash column chromatography (PE/EA 3:1, v/v) to give **36b** (781.72 mg, yield 86.8%) as a light yellow liquid. ^1^H NMR (400 MHz, CDCl_3_) *δ* 7.55 (d, *J* = 1.8 Hz, 1H), 7.42 (d, *J* = 2.3 Hz, 1H), 6.31 (t, *J* = 2.1 Hz, 1H), 5.80 (dd, *J* = 17.5, 10.7 Hz, 1H), 5.06 (d, *J* = 8.0 Hz, 2H), 4.94–4.86 (m, 2H), 4.80 (d, *J* = 3.8 Hz, 4H), 3.43 (s, 4H), 3.06 (d, *J* = 13.8 Hz, 1H), 2.64 (d, *J* = 13.7 Hz, 1H), 2.42–2.31 (m, 2H), 2.22 (s, 2H), 1.91 (d, *J* = 11.1 Hz, 1H), 1.80 (s, 1H), 1.69–1.56 (m, 2H), 1.55–1.50 (m, 1H),1.50–1.425 (m, 12H), 1.00 (s, 3H). LCMS *m/z* [M + H]^+^: 454.9.

##### General procedure for the synthesis of intermediates 37a–b

To a solution of intermediate **36** (1.55 mmol) in MeOH (1.5 ml) in an ice-cooled bath was added a solution of hydrochloride in dioxane (4 M, 6 ml). The mixture was gradually warmed to room temperature and stirred for 8 h. The reaction was monitored by TLC. Upon completion, the solvent was removed under reduced pressure to give intermediates **37** (1.55 mmol, yield 100%) as a white solid. The residue was used for the next reaction without further purification.

##### General procedure for the synthesis of intermediates 38a–f

A solution of **37** (0.41 mmol), DIPEA (1.23 mmol), EDCI (1.07 mmol), HOBt (0.53 mmol) and the corresponding acids **14** (0.49 mmol) in DMF (5 ml) was stirred at room temperature for 5 h. The reaction was monitored by TLC. Upon completion, the mixture was diluted in H_2_O (50 ml) and was extracted with EA (3 × 50 ml). The combined organic layers were washed with water and brine, and dried over Na_2_SO_4_. The drying agent was filtered off. The filtrate was concentrated under reduced pressure and the residue was purified *via* column chromatography (DCM/MeOH 99:1 ∼ 9:1, v/v) to give the products as a light yellow liquid.

##### General procedure for the synthesis of the target compounds 39a–f

To a solution of intermediates **38** (0.37 mmol) in MeOH (3 ml) was added TsOH·H_2_O (0.74 mmol) in room temperature for 8 h. The reaction was monitored by TLC. Upon completion, the mixture was concentrated *in vacuo*. Then, the mixture was diluted in H_2_O (10 ml) and was extracted with DCM (3 × 10 ml). The combined organic layers were washed with water and brine, and dried over Na_2_SO_4_. The drying agent was filtered off. The filtrate was concentrated under reduced pressure and the residue was purified *via* C18 column chromatography (MeCN/H_2_O 1:4 ∼ 4:1, v/v) to afford the products.

###### N^1^-(2-((1R,2S,5R)-5–(3-(1H-pyrazol-1-yl)prop-1-en-2-yl)-2-methyl-2-vinylcyclohexyl)allyl)-N^6^-hydroxyadipamide (39a)

White solid, yield 70.4%, m.p. 61.7–63.3 °C. ^1^H NMR (500 MHz, CDCl_3_) *δ* 7.49 (s, 1H), 7.40 (d, *J* = 2.3 Hz, 1H), 6.28 (t, *J* = 2.1 Hz, 1H), 5.76 (dd, *J* = 17.4, 10.8 Hz, 1H), 5.08–4.62 (m, 8H), 3.83 (dd, *J* = 15.6, 6.4 Hz, 1H), 3.61 (dd, *J* = 15.8, 4.6 Hz, 1H), 2.19 (d, *J* = 28.1 Hz, 4H), 1.93 (d, *J* = 10.9 Hz, 1H), 1.83 (s, 1H), 1.72–1.38 (m, 10H), 0.96 (s, 3H). ^13^C NMR (126 MHz, CDCl_3_) *δ* 173.40, 149.46, 149.14, 147.90, 139.12, 129.66, 112.34, 111.63, 110.98, 106.00, 56.17, 48.97, 46.09, 41.57, 39.68, 39.41, 35.90, 33.45, 32.32, 26.76, 24.93 (d, *J* = 14.8 Hz), 16.04. HRMS (ESI) *m/z*: calcd for C_24_H_36_N_4_O_3_ [M + H]^+^, 429.2860; found, 429.2863.

###### N^1^-(2-((1R,2S,5R)-5–(3-(1H-pyrazol-1-yl)prop-1-en-2-yl)-2-methyl-2-vinylcyclohexyl)allyl)-N^7^-hydroxyheptanediamide (39b)

Colourless viscous colloid, yield 72.5%, m.p. 63.8–64.9 °C. ^1^H NMR (500 MHz, CDCl_3_) *δ* 7.49 (s, 1H), 7.40 (d, *J* = 2.2 Hz, 1H), 6.27 (s, 1H), 5.76 (dd, *J* = 17.4, 10.8 Hz, 1H), 4.97 (d, *J* = 11.2 Hz, 2H), 4.89 (dd, *J* = 14.2, 10.5 Hz, 2H), 4.73 (q, *J* = 9.9, 8.5 Hz, 4H), 3.80 (d, *J* = 17.2 Hz, 1H), 3.63 (d, *J* = 13.7 Hz, 1H), 2.16 (d, *J* = 36.6 Hz, 4H), 1.98–1.79 (m, 2H), 1.72–1.37 (m, 8H), 1.36–1.20 (m, 4H), 0.96 (s, 3H). ^13^C NMR (126 MHz, CDCl_3_) *δ* 173.46, 149.47, 149.18, 147.95, 139.16, 129.61, 111.61, 110.95, 105.94, 56.11, 49.13, 45.89, 41.70, 39.67, 39.40, 36.11, 33.33, 29.70, 26.78, 16.12. HRMS (ESI) *m/z*: calcd for C_25_H_38_N_4_O_3_ [M + H]^+^, 443.3017; found, 443.3014.

###### N^1^-(2-((1R,2S,5R)-5–(3-(1H-pyrazol-1-yl)prop-1-en-2-yl)-2-methyl-2-vinylcyclohexyl)allyl)-N^8^-hydroxyoctanediamide (39c)

White solid, yield 59.7%, m.p. 55.4–57.9 °C. ^1^H NMR (500 MHz, CDCl_3_) *δ* 10.16 (s, 1H), 7.43 (d, *J* = 1.9 Hz, 1H), 7.33 (d, *J* = 2.2 Hz, 1H), 6.21 (t, *J* = 2.1 Hz, 1H), 6.08 (s, 1H), 5.70 (dd, *J* = 17.4, 10.8 Hz, 1H), 4.97–4.79 (m, 4H), 4.75–4.62 (m, 4H), 3.78 (dd, *J* = 15.8, 6.5 Hz, 1H), 3.56 (dd, *J* = 15.8, 4.7 Hz, 1H), 2.09 (dt, *J* = 26.4, 7.4 Hz, 4H), 1.86 (dd, *J* = 12.5, 3.3 Hz, 1H), 1.82–1.72 (m, 1H), 1.59–1.34 (m, 10H), 1.24 (dd, *J* = 7.9, 4.0 Hz, 4H), 0.90 (s, 3H). ^13^C NMR (126 MHz, CDCl_3_) *δ* 173.42, 171.15, 149.42, 149.15, 148.02, 139.12, 129.57, 112.18, 111.68, 110.98, 105.97, 56.14, 49.09, 45.97, 41.63, 39.68, 39.46, 36.29, 33.41, 32.62, 28.42, 28.30, 26.74, 25.41, 25.12, 21.06, 16.03, 14.20. HRMS (ESI) *m/z*: calcd for C_26_H_40_N_4_O_3_ [M + H]^+^, 457.3173; found, 457.3177.

###### 6–(4-(2-((1R,2S,5R)-5–(3-(1H-pyrazol-1-yl)prop-1-en-2-yl)-2-methyl-2-vinylcyclohexyl)allyl)piperazin-1-yl)-N-hydroxy-6-oxohexanamide (39d)

Light yellow solid, yield 72.5%, m.p. 100.2–102.3 °C. ^1^H NMR (500 MHz, CDCl_3_) *δ* 7.50 (d, *J* = 1.9 Hz, 1H), 7.40 (d, *J* = 2.2 Hz, 1H), 6.27 (t, *J* = 2.1 Hz, 1H), 5.75 (dd, *J* = 17.5, 10.8 Hz, 1H), 5.02 (d, *J* = 11.3 Hz, 2H), 4.91–4.83 (m, 2H), 4.76 (d, *J* = 1.8 Hz, 4H), 3.58 (s, 2H), 3.50–3.34 (m, 2H), 3.02 (d, *J* = 13.8 Hz, 1H), 2.60 (d, *J* = 13.8 Hz, 1H), 2.43–2.12 (m, 9H), 1.90 (dt, *J* = 11.4, 6.9 Hz, 1H), 1.71–1.21 (m, 10H), 0.96 (s, 3H). ^13^C NMR (126 MHz, CDCl_3_) *δ* 171.75, 149.75, 149.70, 147.71, 139.16, 129.35, 114.09, 111.60, 110.42, 105.79, 66.01, 56.31, 53.09, 52.89, 47.77, 45.64, 41.90, 39.77 (d, *J* = 7.9 Hz), 36.50, 33.57, 32.68, 26.97, 25.28, 24.63, 15.86. HRMS (ESI) *m/z*: calcd for C_28_H_43_N_5_O_3_ [M + H]^+^, 498.3439; found, 498.3440.

###### 7–(4-(2-((1R,2S,5R)-5–(3-(1H-pyrazol-1-yl)prop-1-en-2-yl)-2-methyl-2-vinylcyclohexyl)allyl)piperazin-1-yl)-N-hydroxy-7-oxoheptanamide (39e)

Light yellow solid, yield 70.8%, m.p. 60.2–61.3 °C. ^1^H NMR (500 MHz, CDCl_3_) *δ* 7.43 (d, *J* = 1.9 Hz, 1H), 7.33 (d, *J* = 2.3 Hz, 1H), 6.21 (t, *J* = 2.1 Hz, 1H), 5.69 (dd, *J* = 17.5, 10.8 Hz, 1H), 4.96 (d, *J* = 10.5 Hz, 2H), 4.89–4.63 (m, 6H), 3.61–3.28 (m, 4H), 2.98 (d, *J* = 13.6 Hz, 1H), 2.55 (d, *J* = 13.7 Hz, 1H), 2.41–2.04 (m, 9H), 1.83 (ddt, *J* = 11.6, 8.2, 3.3 Hz, 1H), 1.66–1.23 (m, 12H), 0.90 (s, 3H). ^13^C NMR (126 MHz, CDCl_3_) *δ* 170.80, 170.08, 148.66 (d, *J* = 4.8 Hz), 146.59, 138.13, 128.37, 113.38, 110.70, 109.44, 104.83, 65.08, 55.36, 52.09, 51.79, 46.76, 44.67, 40.75 (d, *J* = 9.0 Hz), 38.74 (d, *J* = 2.3 Hz), 32.62, 31.76, 31.36, 27.41, 25.93, 23.98, 23.45, 14.81. HRMS (ESI) *m/z*: calcd for C_29_H_45_N_5_O_3_ [M + H]^+^, 512.3595; found, 512.3596.

###### 8–(4-(2-((1R,2S,5R)-5–(3-(1H-pyrazol-1-yl)prop-1-en-2-yl)-2-methyl-2-vinylcyclohexyl)allyl)piperazin-1-yl)-N-hydroxy-8-oxooctanamide (39f)

Colourless viscous colloid, yield 60.3%, m.p. 58.7–59.5 °C. ^1^H NMR (500 MHz, CDCl_3_) *δ* 7.50 (d, *J* = 1.8 Hz, 1H), 7.40 (d, *J* = 2.2 Hz, 1H), 6.28 (t, *J* = 2.1 Hz, 1H), 5.76 (dd, *J* = 17.5, 10.7 Hz, 1H), 5.03 (d, *J* = 10.4 Hz, 2H), 4.95–4.83 (m, 2H), 4.83–4.68 (m, 4H), 3.58 (s, 2H), 3.45 (tdd, *J* = 15.2, 7.5, 4.0 Hz, 2H), 3.04 (d, *J* = 13.6 Hz, 1H), 2.61 (d, *J* = 13.7 Hz, 1H), 2.49–2.10 (m, 9H), 1.96–1.84 (m, 1H), 1.74–1.18 (m, 14H), 0.97 (s, 3H). ^13^C NMR (126 MHz, CDCl_3_) *δ* 171.89, 149.72, 149.68, 147.66, 139.15, 129.37, 114.31, 111.71, 110.44, 105.84, 66.12, 56.38, 53.19, 52.84, 47.78, 45.75, 41.82, 41.70, 39.76 (d, *J* = 2.4 Hz), 33.64, 32.95, 32.44, 32.42, 28.47 (d, *J* = 18.9 Hz), 26.96, 25.01 (d, *J* = 17.6 Hz), 15.84. HRMS (ESI) *m/z*: calcd for C_30_H_47_N_5_O_3_ [M + H]^+^, 526.3752; found, 526.3752.

##### Synthesis of the compound 40

(i) A solution of **37a** (0.35 mmol), DIPEA (1.05 mmol), EDCI (0.91 mmol), HOBt (0.46 mmol) and the acid (**12c**, 0.42 mmol) in DMF (3 ml) was stirred at room temperature for 5 h. The reaction was monitored by TLC. Upon completion, the residue was diluted in H_2_O (30 ml) and was extracted with EA (3 × 30 ml). The combined organic layers were washed with water and brine, and dried over Na_2_SO_4_. The drying agent was filtered off. The filtrate was concentrated under reduced pressure and the residue was purified *via* column chromatography (DCM/MeOH 49:1, v/v) to give the corresponding ester (153.00 mg, yield 95.7%) as a light yellow liquid.

(ii) A solution of the ester (0.28 mmol) and cooled NaOH aqueous solution (1 N, 3 ml) in MeOH (3 ml) was stirred at room temperature for 2 h. The reaction was monitored by TLC. Upon completion, the mixture was concentrated in vacuo. Then, the residue was neutralised by HCl solution (1 M) to pH 6 ∼ 7 and was extracted with EA (3 × 20 ml). The combined organic layers were washed with water and brine, and dried over Na_2_SO_4_. The drying agent was filtered off. The filtrate was concentrated under reduced pressure to afford the crude product **40** (yield 92.3%) as a light yellow liquid. It was directly used in the next reaction without purification.

##### Synthesis of the target compound 41

A solution of **40** (0.12 mmol), DIPEA (0.36 mmol), EDCI (0.31 mmol), HOBt (0.16 mmol) and *o*-phenylenediamine **29** (0.60 mmol) in DMF (3 ml) was stirred at room temperature for 5 h. The reaction was monitored by TLC. Upon completion, the mixture was diluted in H_2_O (30 ml) and was extracted with EA (3 × 30 ml). The combined organic layers were washed with water and brine, and dried over Na_2_SO_4_. The drying agent was filtered off. The filtrate was concentrated under reduced pressure and the residue was purified *via* C18 column chromatography (MeCN/H_2_O 2:3 ∼ 1:1, v/v) to afford **41** (56.11 mg, yield 88.0%) as a light yellow solid, m.p. 98.2–100.3 °C. ^1^H NMR (500 MHz, CDCl_3_) *δ* 7.83 (s, 1H), 7.51 (d, *J* = 1.9 Hz, 1H), 7.39 (d, *J* = 2.2 Hz, 1H), 7.16 (dd, *J* = 7.7, 1.4 Hz, 1H), 7.03 (td, *J* = 7.7, 1.5 Hz, 1H), 6.76 (t, *J* = 7.6 Hz, 2H), 6.28 (t, *J* = 2.1 Hz, 1H), 5.75 (dd, *J* = 17.5, 10.8 Hz, 1H), 4.99 (s, 1H), 4.97–4.86 (m, 3H), 4.79–4.69 (m, 4H), 3.84 (dd, *J* = 15.8, 6.8 Hz, 1H), 3.62 (dd, *J* = 15.8, 5.0 Hz, 1H), 2.37 (t, *J* = 7.5 Hz, 2H), 2.19 (t, *J* = 7.4 Hz, 2H), 1.94–1.80 (m, 2H), 1.77–1.49 (m, 5H), 1.48–1.24 (m, 9H), 0.96 (s, 3H). ^13^C NMR (126 MHz, CDCl_3_) *δ* 172.90, 172.03, 149.41, 149.15, 148.09, 140.83, 139.19, 129.42, 126.98, 125.24, 124.47, 119.31, 118.10, 112.11, 111.70, 110.98, 105.88, 56.18, 49.12, 45.89, 41.73, 39.69, 39.48, 36.65, 36.39, 33.38, 28.62, 28.23, 26.80, 25.51, 25.29, 15.98. HRMS (ESI) *m/z*: calcd for C_32_H_45_N_5_O_2_ [M + H]^+^, 532.3646; found, 532.3647.

##### Synthesis of intermediate 42

A solution of **37b** (0.37 mmol), DIPEA (0.44 mmol) and the benzyl chloride **16** (0.44 mmol) in DMF (3 ml) was stirred at 60 °C for 6 h. The reaction was monitored by TLC. Upon completion, the mixture was diluted in H_2_O (30 ml) and was extracted with EA (3 × 30 ml). The combined organic layers were washed with water and brine, and dried over Na_2_SO_4_. The drying agent was filtered off. The filtrate was concentrated under reduced pressure and the residue was purified *via* flash column chromatography (DCM/MeOH 91:9, v/v) to give **42** (196.39 mg, yield 90.3%) as a colourless liquid. ^1^H NMR (500 MHz, CDCl_3_) *δ* 7.76–7.67 (m, 2H), 7.49 (d, *J* = 1.9 Hz, 1H), 7.42–7.34 (m, 3H), 6.25 (t, *J* = 2.1 Hz, 1H), 5.74 (dd, *J* = 17.5, 10.8 Hz, 1H), 5.10–5.03 (m, 2H), 4.99 (s, 1H), 4.88–4.71 (m, 6H), 4.00 (td, *J* = 10.4, 9.1, 2.9 Hz, 1H), 3.63 (dd, *J* = 10.4, 4.9 Hz, 1H), 3.60 (s, 2H), 3.09 (d, *J* = 13.5 Hz, 1H), 2.68 (d, *J* = 13.6 Hz, 1H), 2.62–2.22 (m, 8H), 2.15 (dd, *J* = 12.6, 3.6 Hz, 1H), 1.96–1.76 (tdd, *J* = 17.0, 12.5, 9.5 Hz, 4H), 1.75–1.35 (m, 9H), 0.94 (s, 3H).

##### Synthesis of the target compound 43

To a solution of **42** (0.26 mmol) in MeOH (2 ml) was added TsOH·H_2_O (0.52 mmol) and stirred at room temperature for 8 h. The reaction was monitored by TLC. Upon completion, the mixture was concentrated *in vacuo*. Then, the residue was diluted in H_2_O (10 ml) and was extracted with DCM (3 × 10 ml). The organic layer was separated, washed with saturated NaHCO_3_ solution and brine, and dried over Na_2_SO_4_. The drying agent was filtered off. The filtrate was concentrated under reduced pressure and the residue was purified *via* C18 column chromatography (MeCN/H_2_O 1:1, v/v) to afford the target compound **43** (95.73 mg, yield 73.1%) as a white solid, m.p. 103.2–105.3 °C. ^1^H NMR (500 MHz, CDCl_3_) *δ* 7.65 (d, *J* = 7.7 Hz, 2H), 7.48 (d, *J* = 1.9 Hz, 1H), 7.38 (d, *J* = 2.3 Hz, 1H), 7.32 (d, *J* = 7.7 Hz, 2H), 6.25 (t, *J* = 2.1 Hz, 1H), 5.74 (dd, *J* = 17.5, 10.8 Hz, 1H), 5.00 (d, *J* = 11.1 Hz, 2H), 4.90–4.69 (m, 6H), 3.53 (s, 2H), 3.02 (d, *J* = 13.6 Hz, 1H), 2.61 (d, *J* = 13.6 Hz, 1H), 2.45 (s, 8H), 2.14 (dd, *J* = 11.7, 4.4 Hz, 1H), 1.89 (dq, *J* = 11.8, 4.4, 4.0 Hz, 1H), 1.64–1.35 (m, 6H), 0.94 (s, 3H). ^13^C NMR (126 MHz, CDCl_3_) *δ* 149.72 (d, *J* = 4.0 Hz), 147.70, 139.17, 129.50 (d, *J* = 11.8 Hz), 126.95, 114.32, 111.50, 110.35, 105.83, 66.20, 62.49, 56.34, 52.87 (d, *J* = 14.0 Hz), 47.94, 41.81, 39.69, 33.64, 26.90, 15.85. HRMS (ESI) *m/z*: calcd for C_30_H_41_N_5_O_2_ [M + H]^+^, 504.3333; found, 504.3337.

### Biological evaluation

#### HDAC inhibition assay

All HDAC enzymes were purchased from BPS Bioscience. The various concentrations of the tested compounds and HDAC isoforms were mixed into a 384-well plate and incubated at 37 °C for 10 min. Then the diluted HDAC substrate was added into each well and incubated at 37 °C for 30 min. The reaction was stopped by adding developer, and the mixture was incubated at 37 °C for another 20 min. The fluorescence intensity was measured at the excitation and emission wavelengths of 355 nm and 460 nm with a microplate reader. The IC_50_ values were calculated according to the dose dependent curves. All the tests were repeated in three independent experiments.

#### In vitro anti-proliferative assay

The anti-proliferative activities of the target compounds were determined using CCK8 assay kit. 80 µL of cell suspensions (5.0 × 10^4^ cell/ml) (K562, MV4-11, HEL, SU-DHL-2, WSU-DLCL-2 and U87MG) were added to a 96-well cell culture plate and incubated for 24 h at 37 °C under an atmosphere of 5% CO_2_. The tested compounds were dissolved in the culture medium with 0.5% DMSO at the different concentrations. The cells were treated with the drug solution for another 72 h. Then 10 µL of cell counting kit-8 (CCK8) solution was added to each well and the plate was incubated for an additional 1 h. The IC_50_ values were calculated according to the dose dependent curves. All the tests were repeated in three independent experiments.

#### Apoptosis assay

WSU-DLCL-2 cells were seeded into 6-well plates and incubated at 37 °C for 24 h, and then treated with the tested compounds at the different concentrations or without the tested compound for another 72 h. The cells were then harvested by trypsinization and washed twice with cold PBS. After the centrifugation and removal of the supernatants, cells were re-suspended in 500 µL of a 1 × binding buffer, which was then added to 5 µL of annexin V-FITC and 10 µL of PI, and incubated at room temperature for 15 min in the dark. The stained cells were analysed by a flow cytometer.

#### Cell cycle assay

WSU-DLCL-2 cells were seeded into 6-well plates and incubated at 37 °C for 24 h, and then treated with the tested compounds at the different concentrations or without the tested compound for another 72 h. After treatment, cells were collected and fixed with 70% pre-cold ethanol in PBS and stored at −20 °C overnight. Then washed the cells with PBS twice, and incubated with 100 µg/ml RNase A at 37 °C for 1 h, stained with propidium iodide for 30 min avoid light at room temperature. The stained cells were analysed by a flow cytometer.

#### Molecular docking

The molecular docking was performed using energetics (CDOCER) software (Discovery Studio^®^ 2020). The two-dimensional structure of HDAC1 (PDB code: 5ICN) and HDAC6 (PDB code: 5EDU) was performed from the Protein Data Bank. The binding water and ligands were deleted and the polar hydrogen was added. The molecular structures of compounds **27f** and **39f** were optimised using Tripos force field. The molecular docking of **27f** and **39f** with HDAC1 and HDAC6 was carried out with Discovery Studio^®^ 2020.

#### Pharmacokinetics study

The experimental procedures and the animal use and care protocols were approved by the Committee on Ethical Use of Animals of Hangzhou Normal University. Compound **27f** was subjected to PK studies in ICR (SPF) female mice. Compound **27f** was dissolved in the solution of 0.5% methylcellulose (containing 0.4% Tween 80) and then administrated via the oral route at 10 mg/kg (twelve rats). Compound **27f** was dissolved in the solution of 5% dimethyl sulfoxide (DMSO), 5% Solutol, and 90% saline and then administrated via the intravenous route at 5 mg/kg (twelve rats). At time points 0 (prior to dosing), 5, 15, 30, 60, 120, 240, 480, and 1440 min after dosing, the blood sample was collected from each animal, and the plasma was separated from the blood by centrifugation and stored in a freezer at −80 °C. All samples for the tested compound were analysed by liquid chromatography-tandem mass spectrometry (LC-MS/MS).

## Supplementary Material

Supplemental Material
