# Supplementary material for: Design, synthesis and biological evaluation of novel histone deacetylase (HDAC) inhibitors derived from β-elemene scaffold
Source: J Enzyme Inhib Med Chem. 2023 Apr 4;38(1):2195991. doi: 10.1080/14756366.2023.2195991 (PMC10075517; doi:10.1080/14756366.2023.2195991)
Supplement: Supplemental Material [file IENZ_A_2195991_SM9030.pdf]

Supporting Information

Design, synthesis and biological evaluation of novel histone deacetylase (HDAC) inhibitors derived from *β*-elemene scaffold

Yuan Gao [a, b], Jilong Duan [b], Xiawen Dang [b], Yinghui Yuan [b], Yu Wang [c], Xingrui He [b, *], Renren Bai [b, *], Xiang-Yang Ye [b, *], Tian Xie [a, b, *]

[a]*Institute of Traditional Chinese Medicine, Shanghai University of Traditional Chinese Medicine, Shanghai 200000, China*

[b]*Key Laboratory of Elemene Class Anti-Cancer Chinese Medicines; Engineering Laboratory of Development and Application of Traditional Chinese Medicines; Collaborative Innovation Center of Traditional Chinese Medicines of Zhejiang Province, Hangzhou Normal University, Hangzhou, Zhejiang 311121, China.*

[c]*Research & Development, Dalian HolleyKingkong Pharmaceutical Co. Ltd., Liaoning 116199, China*

Contents

^{1}H and ^{13}C NMR spectra of compounds **18a-f**, **20**, **27a-f**, **31-32**, **34**, **39a-f**, **41** and **43**

All the gating process figures related to cell apoptosis

All the gating process figures related to cell cycle

^{1}H and ^{13}C NMR spectra of compounds 18a-f, 20, 27a-f, 31-32, 34, 39a-f, 41 and 43

N^{1}-hydroxy-N^{6}-(2-((1R,3S,4S)-4-methyl-3-(prop-1-en-2-yl)-4-vinylcyclohexyl)allyl)adipamide (**18a**)

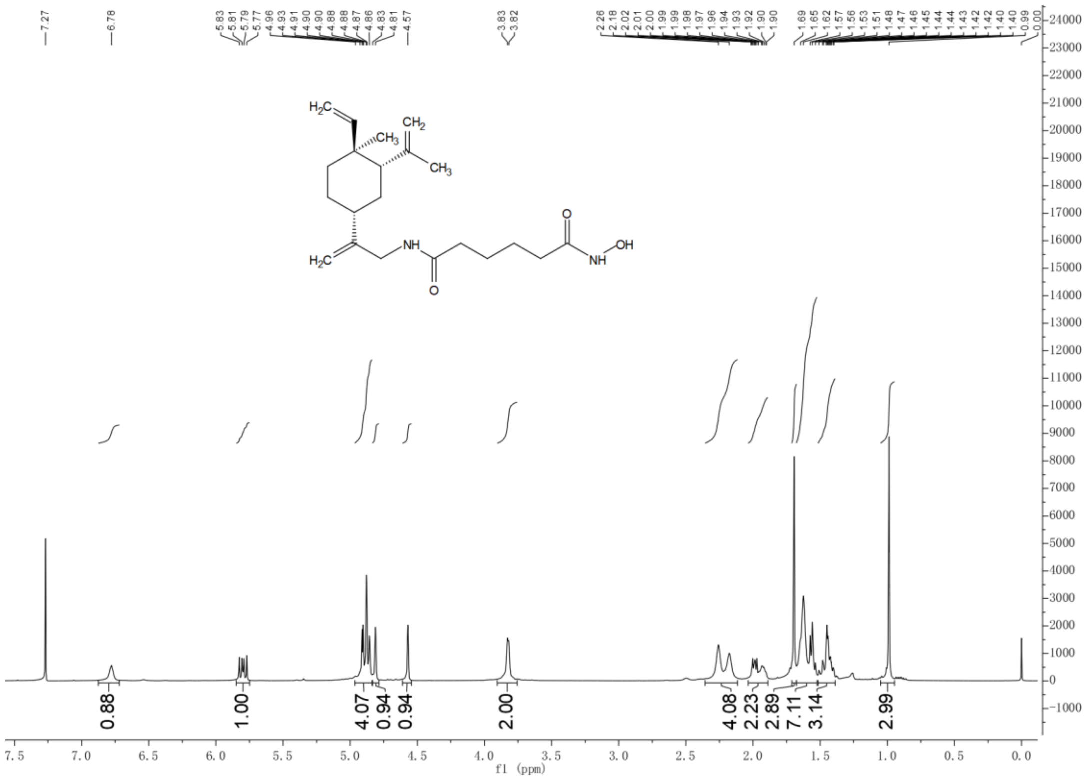

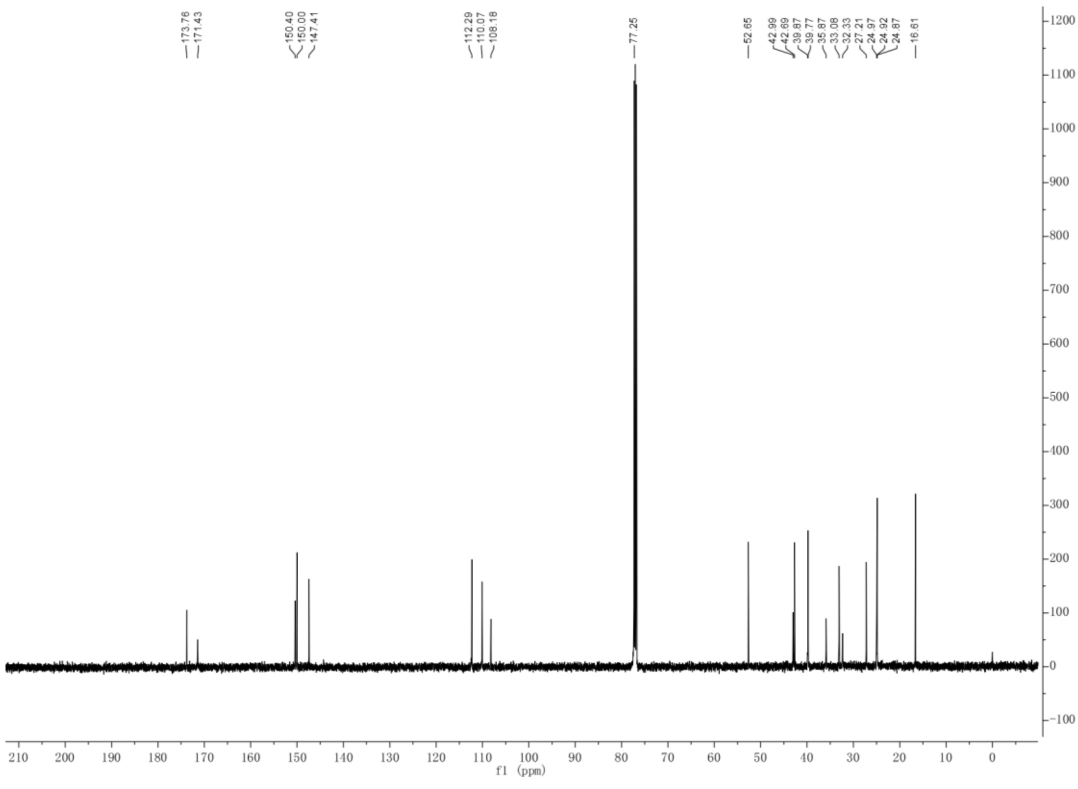

N^1-hydroxy-N^7-(2-((1R,3S,4S)-4-methyl-3-(prop-1-en-2-yl)-4-vinylcyclohexyl)allyl)heptanediamide (**18b**)

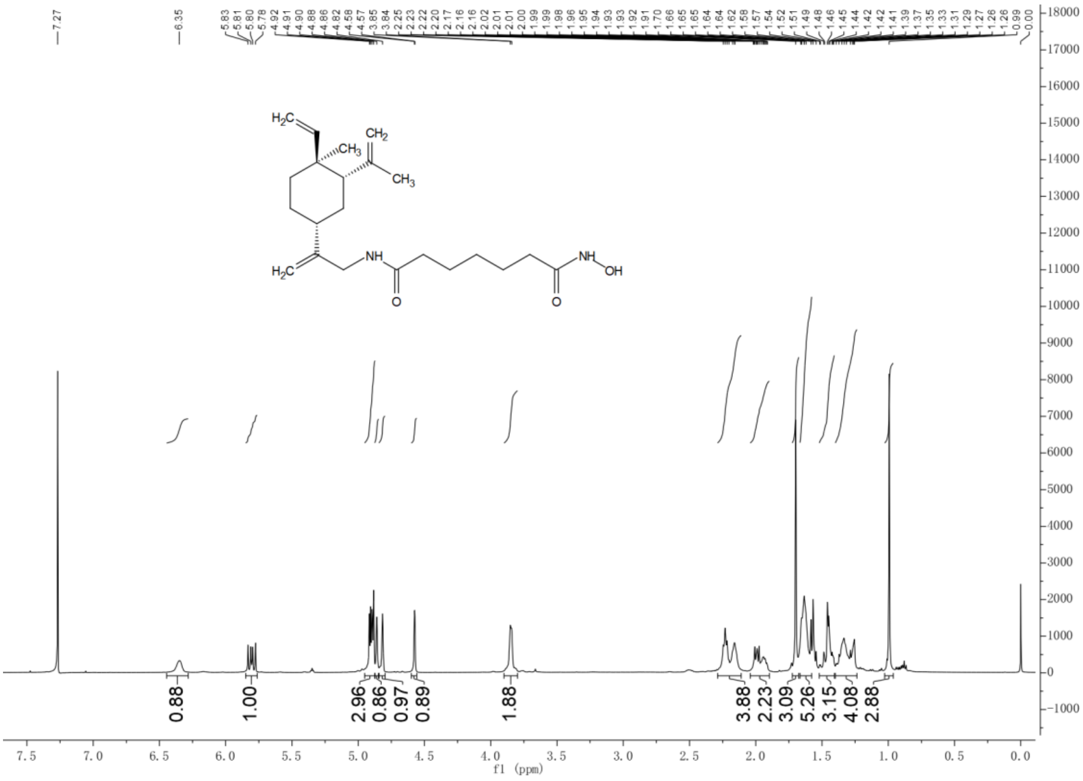

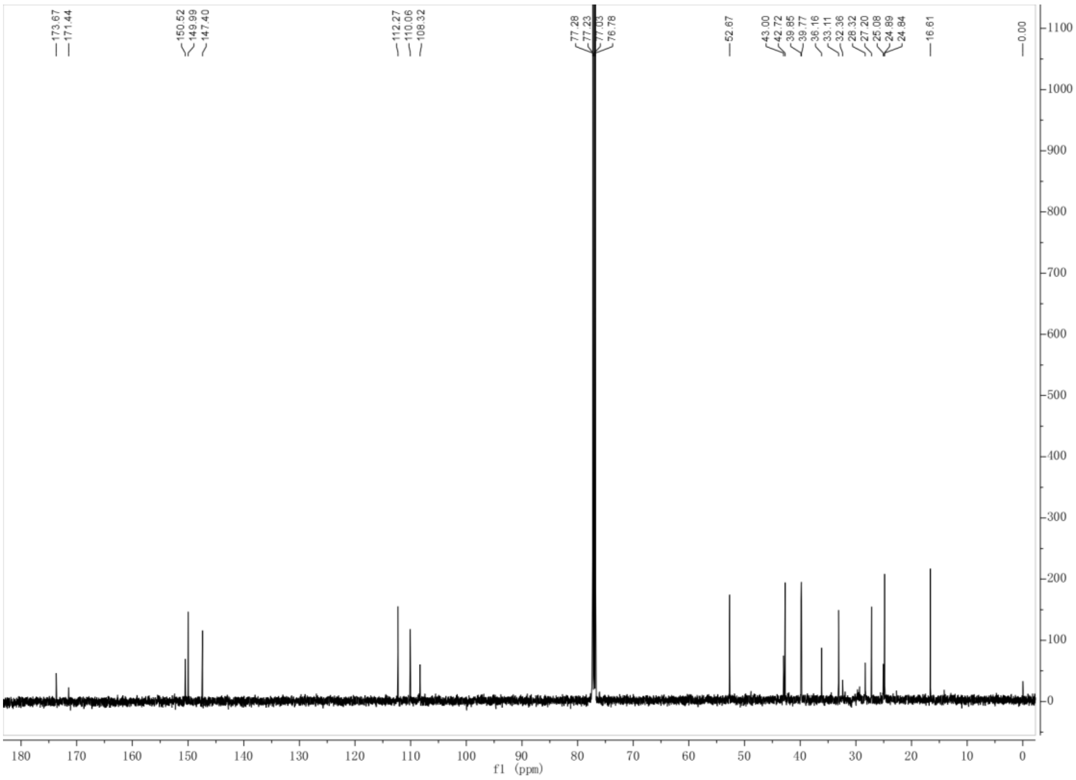

N[1]-hydroxy-N[8]-(2-((1R,3S,4S)-4-methyl-3-(prop-1-en-2-yl)-4-vinylcyclohexyl)allyl)octanediamide (**18c**)

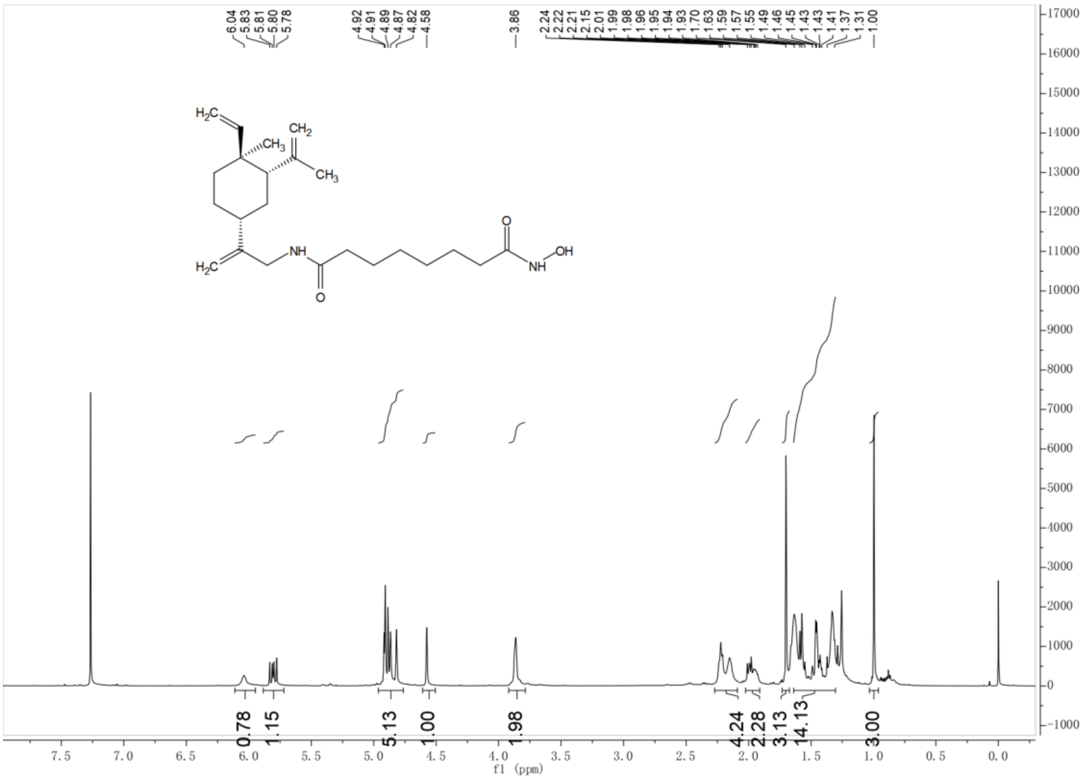

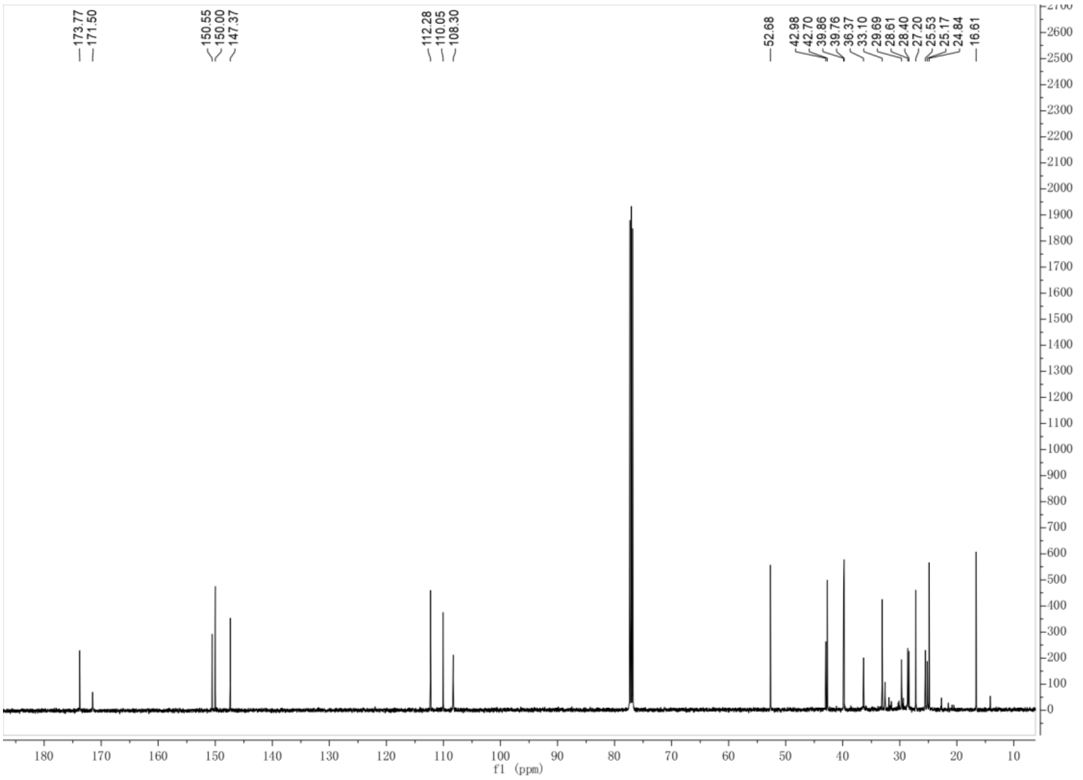

N-hydroxy-6-(4-(2-((1R,3S,4S)-4-methyl-3-(prop-1-en-2-yl)-4-vinylcyclohexyl)allyl)piperazin-1-yl)-6-oxohexanamide (**18d**)

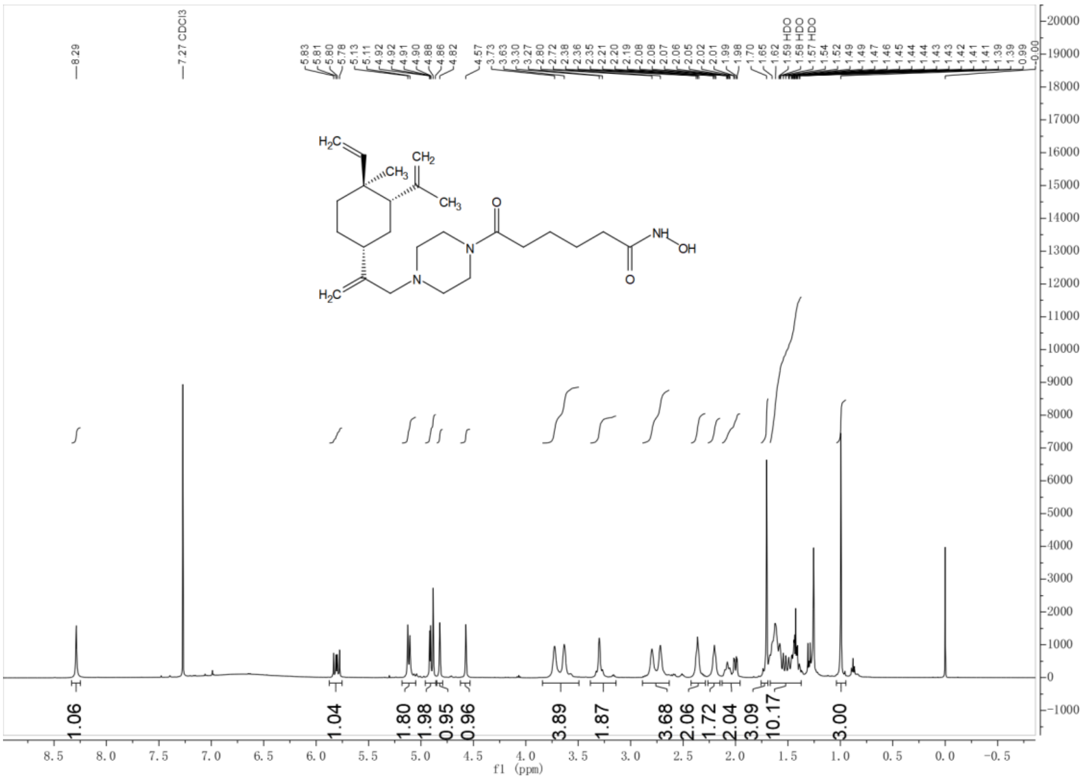

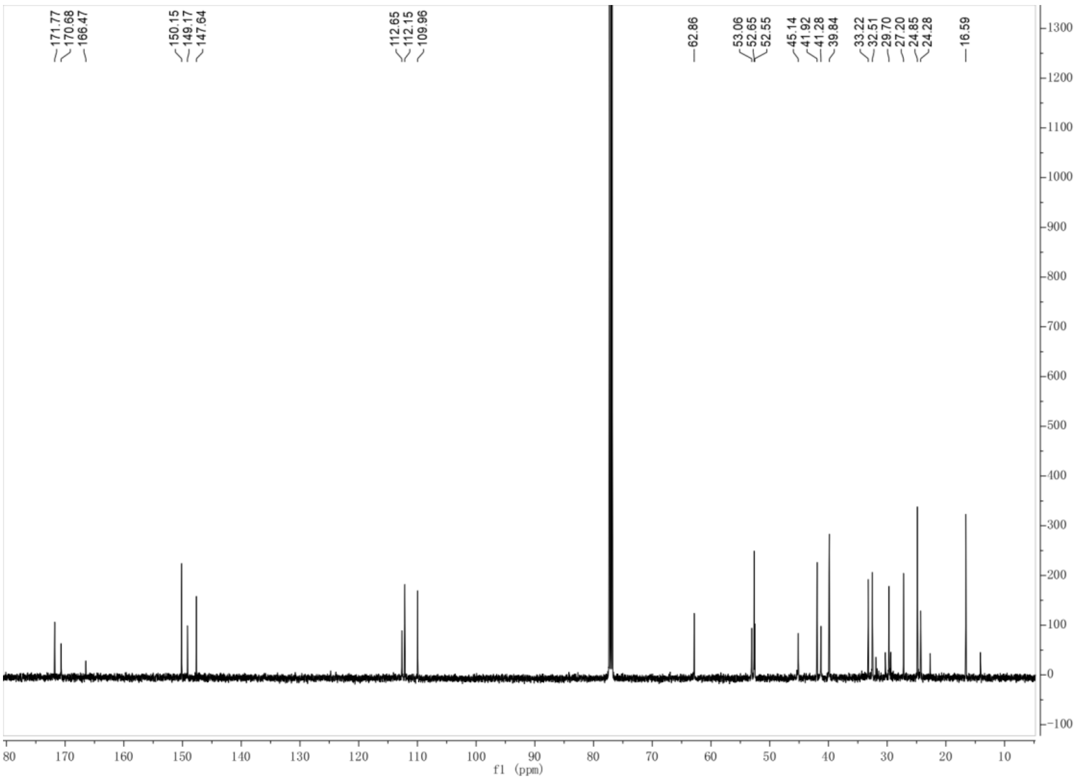

N-hydroxy-7-(4-(2-((1R,3S,4S)-4-methyl-3-(prop-1-en-2-yl)-4-vinylcyclohexyl)allyl)piperazin-1-yl)-7-oxoheptanamide **(18e)**

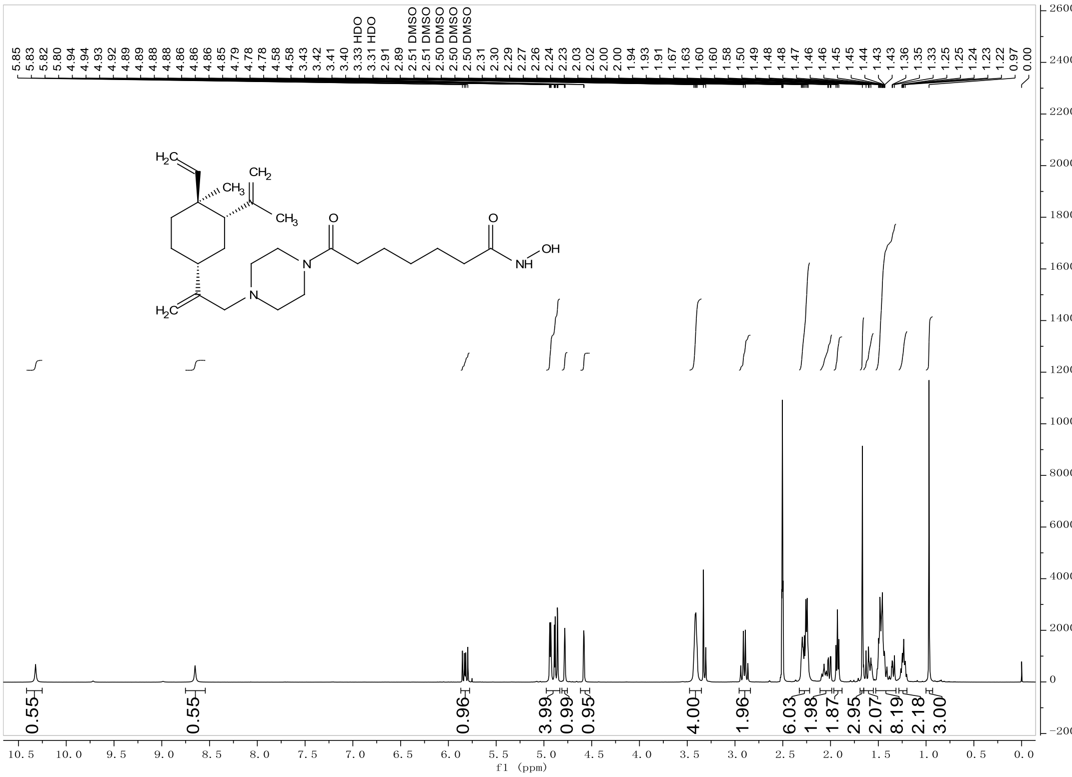

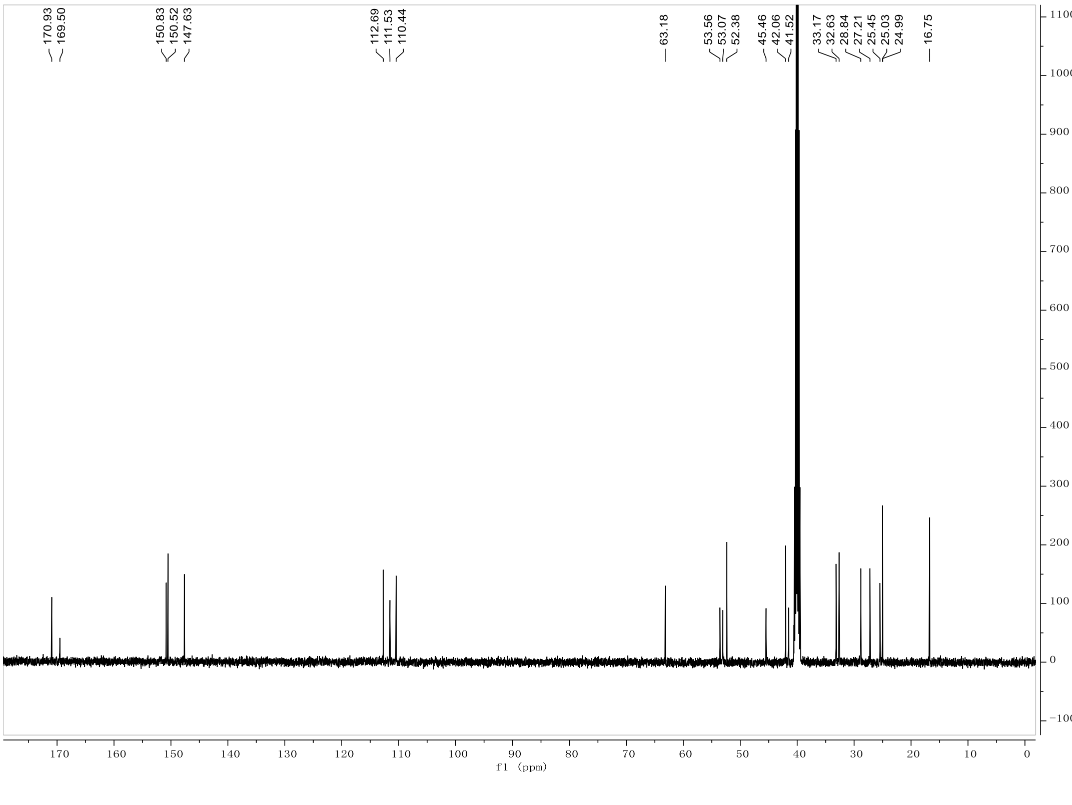

N-hydroxy-8-(4-(2-((1R,3S,4S)-4-methyl-3-(prop-1-en-2-yl)-4-vinylcyclohexyl)allyl)piperazin-1-yl)-8-oxooctanamide (**18f**)

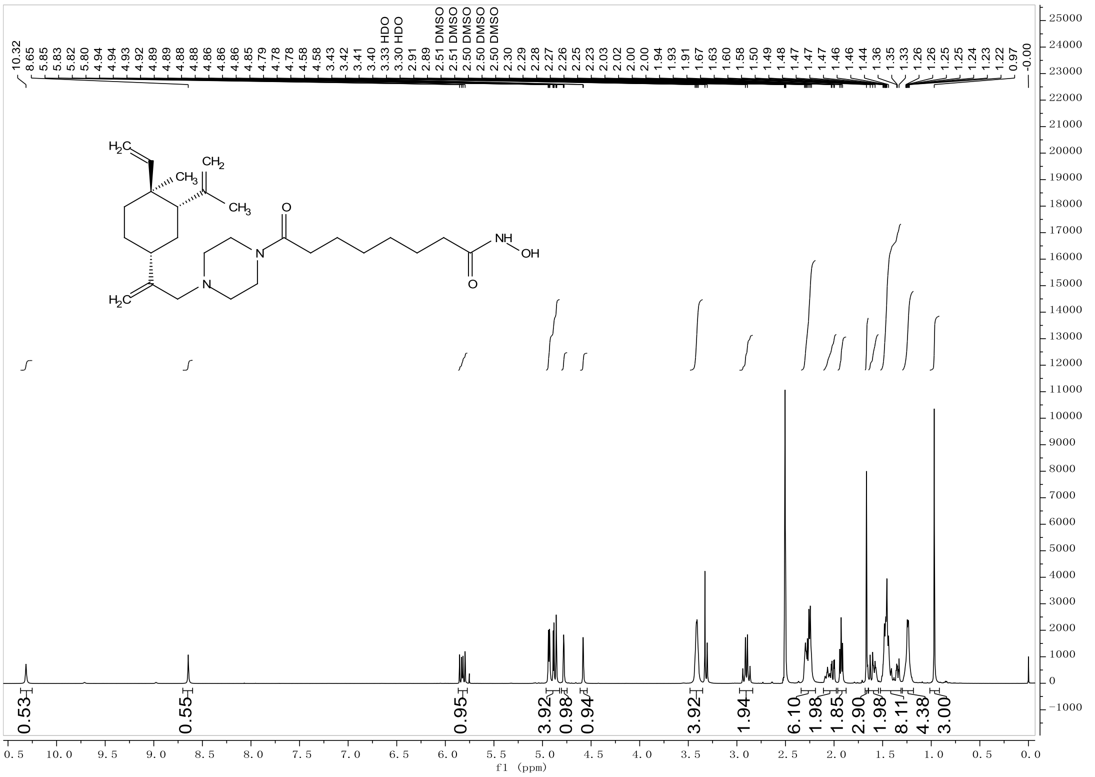

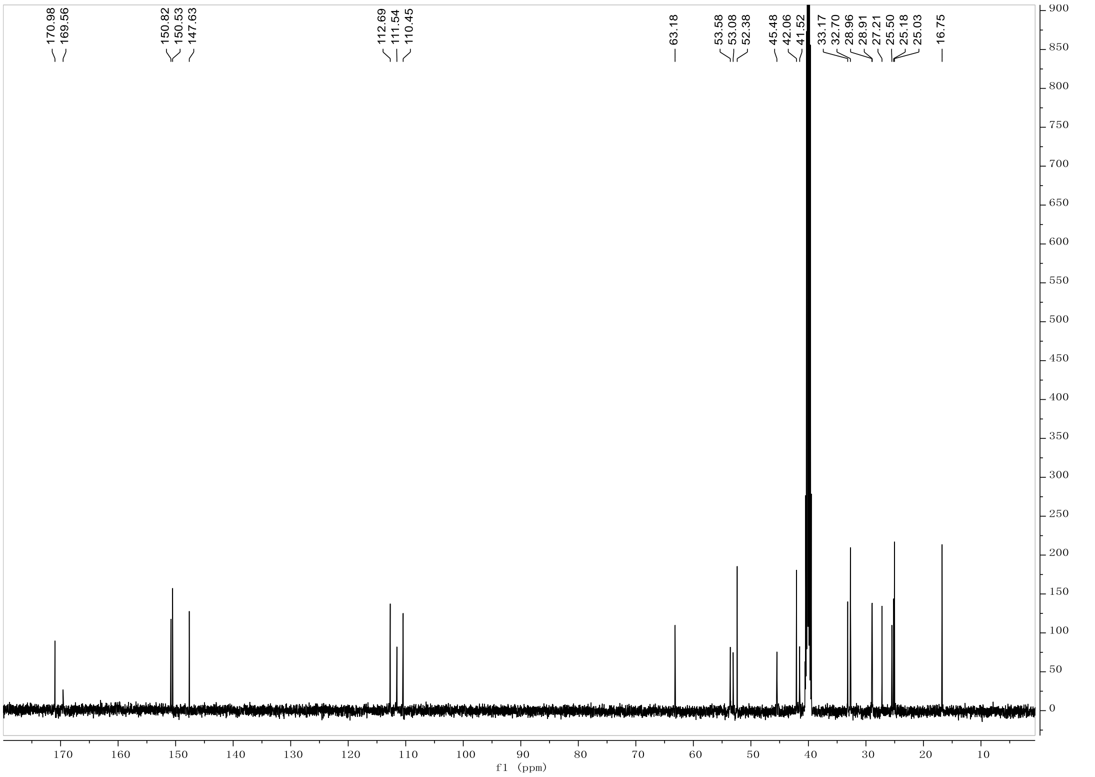

N-hydroxy-4-((4-(2-((1R,3S,4S)-4-methyl-3-(prop-1-en-2-yl)-4-vinylcyclohexyl)allyl)piperazin-1-yl)methyl)benzamide (**20**)

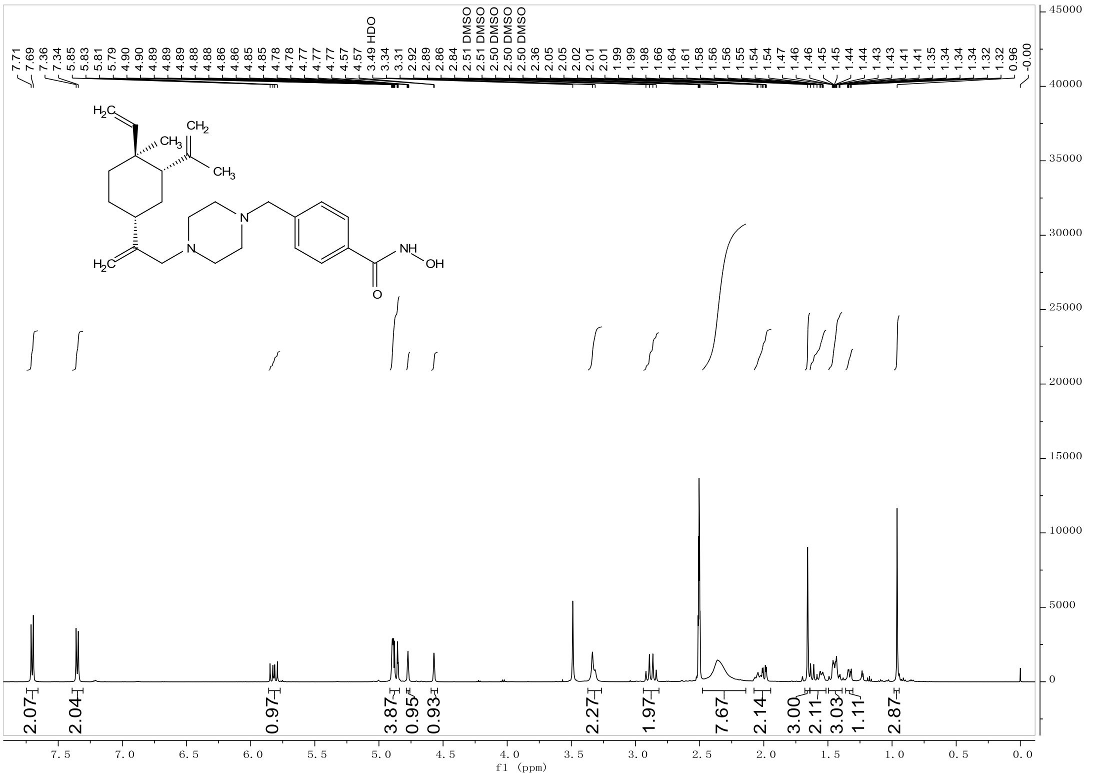

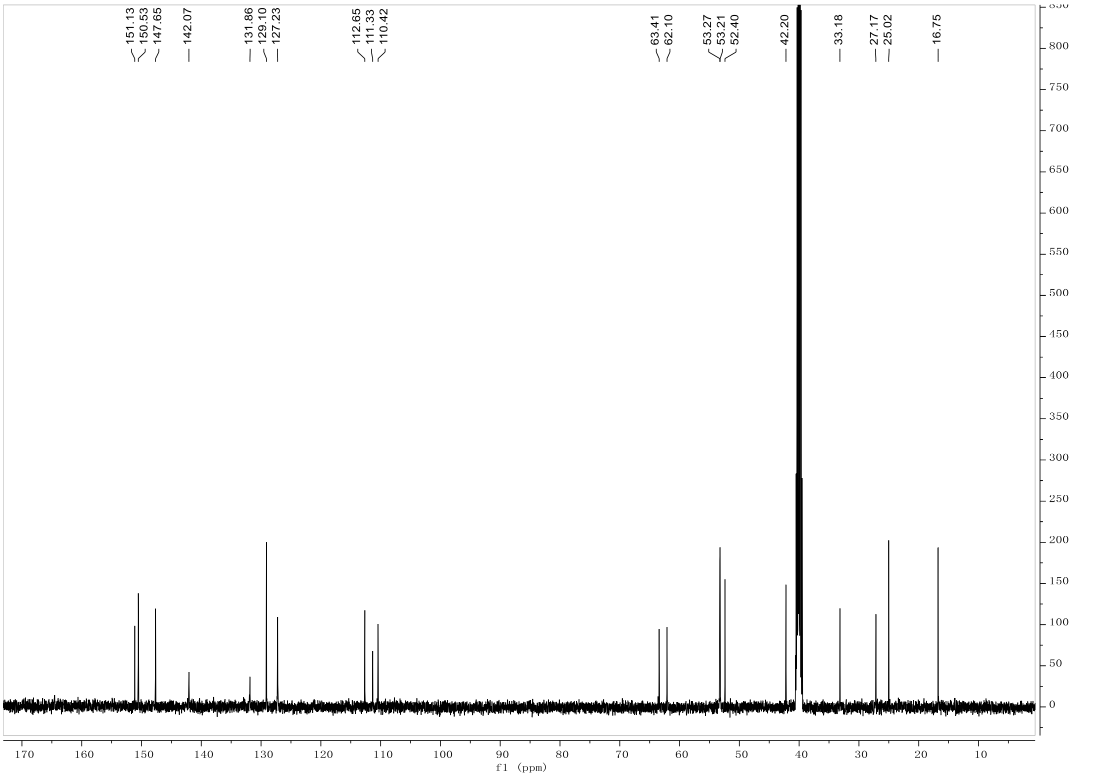

N[1]-(2-((1R,3R,4S)-3-(3-(1H-pyrazol-1-yl)prop-1-en-2-yl)-4-methyl-4-vinylcyclohexyl) allyl)-N[6]-hydroxyadipamide (**27a**)

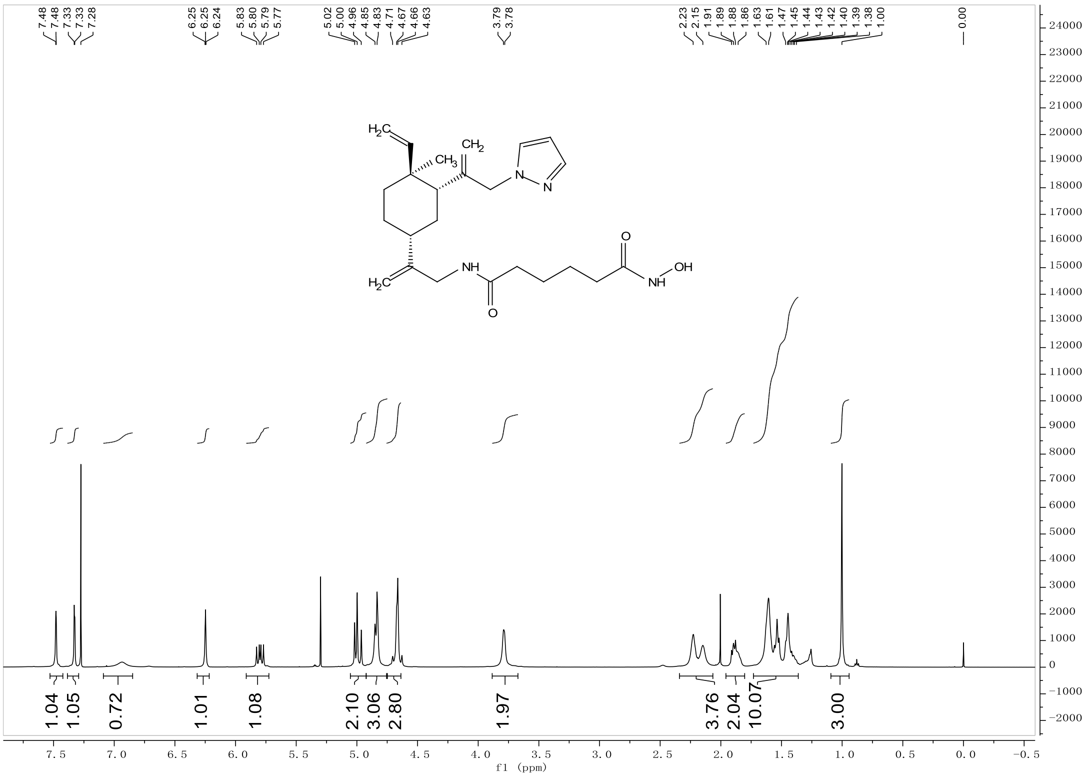

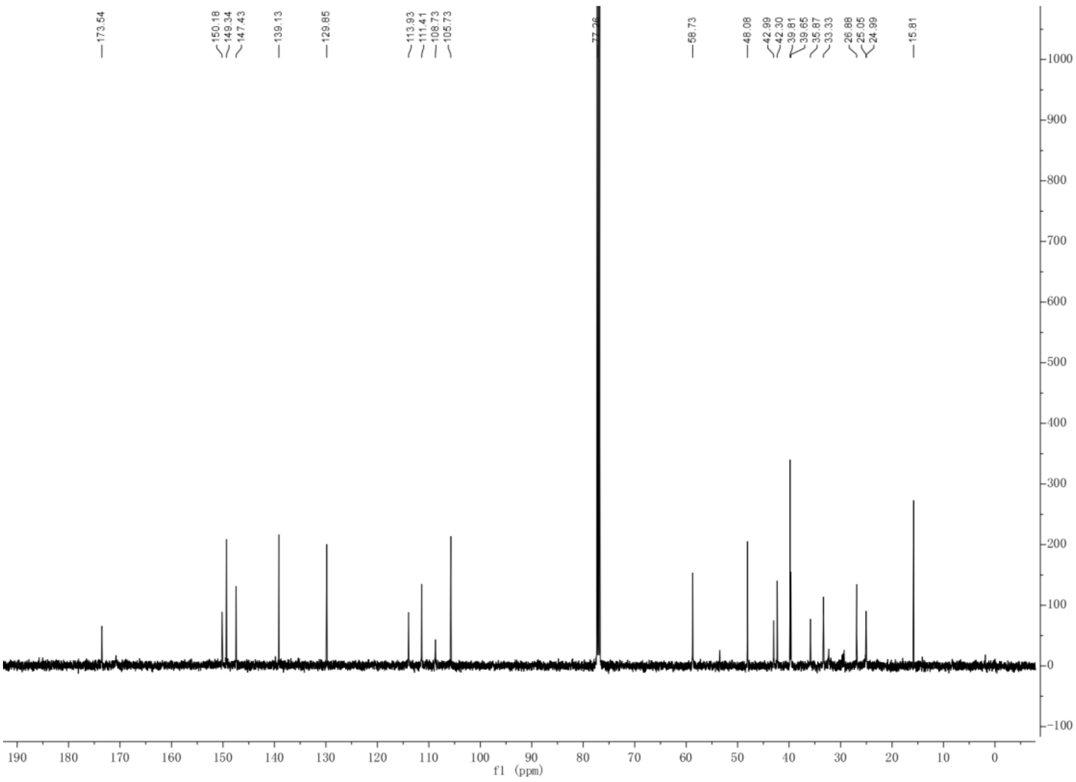

N[1]-(2-((1R,3R,4S)-3-(3-(1H-pyrazol-1-yl)prop-1-en-2-yl)-4-methyl-4-vinylcyclohexyl)allyl)-*N*[7]-hydroxyheptanediamide (**27b**)

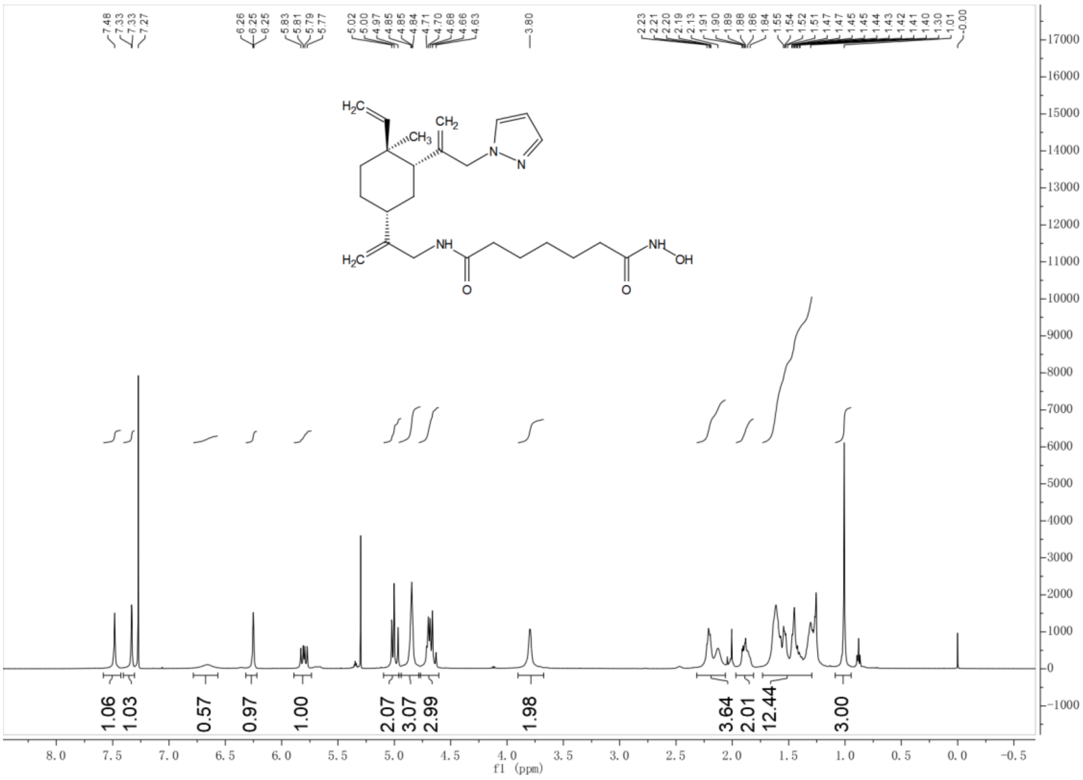

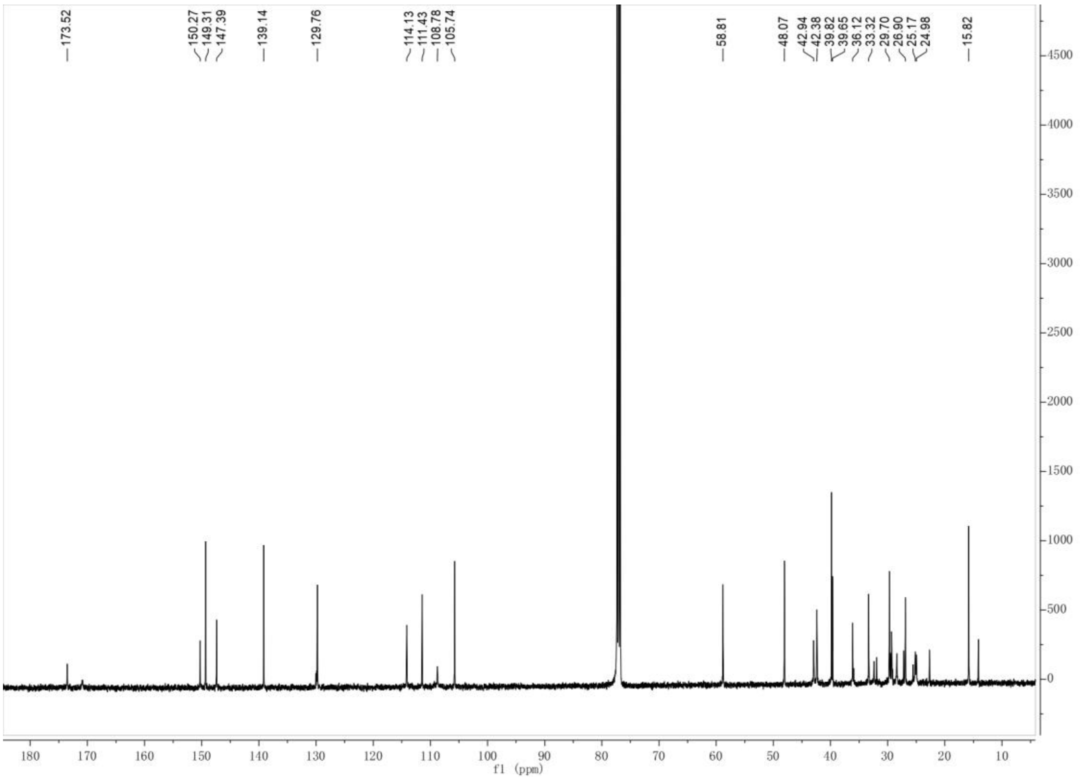

N[1]-(2-((1R,3R,4S)-3-(3-(1H-pyrazol-1-yl)prop-1-en-2-yl)-4-methyl-4-vinylcyclohexyl) allyl)-N[8]-hydroxyoctanediamide (**27c**)

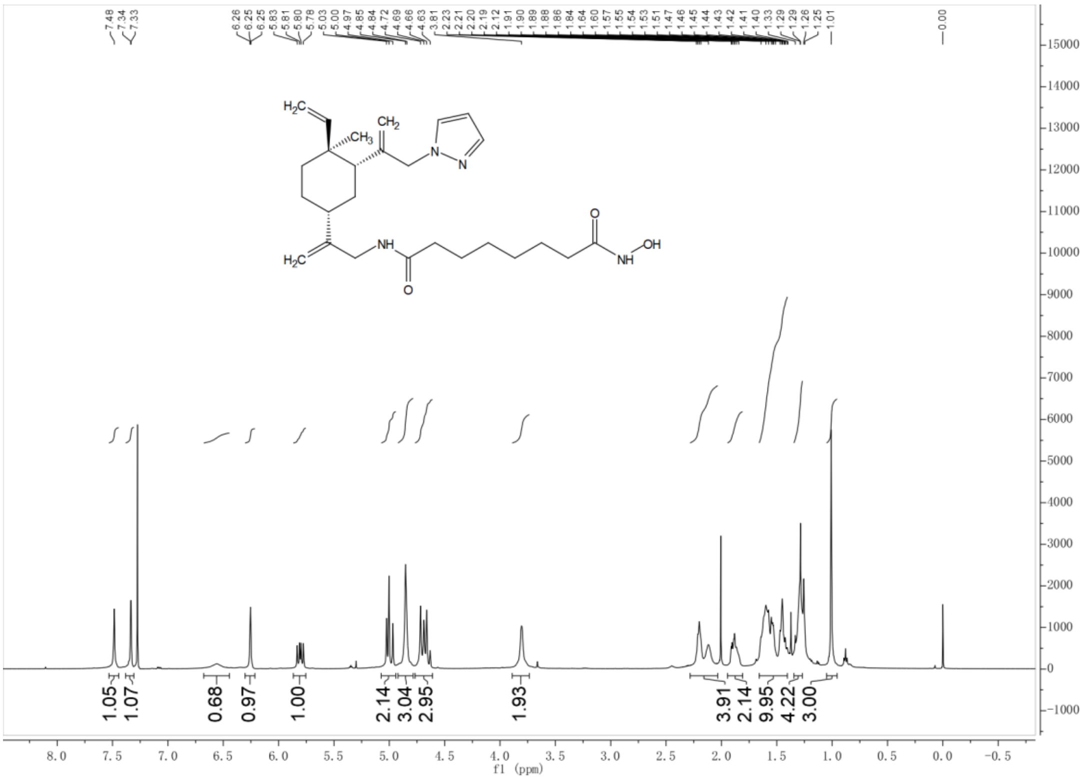

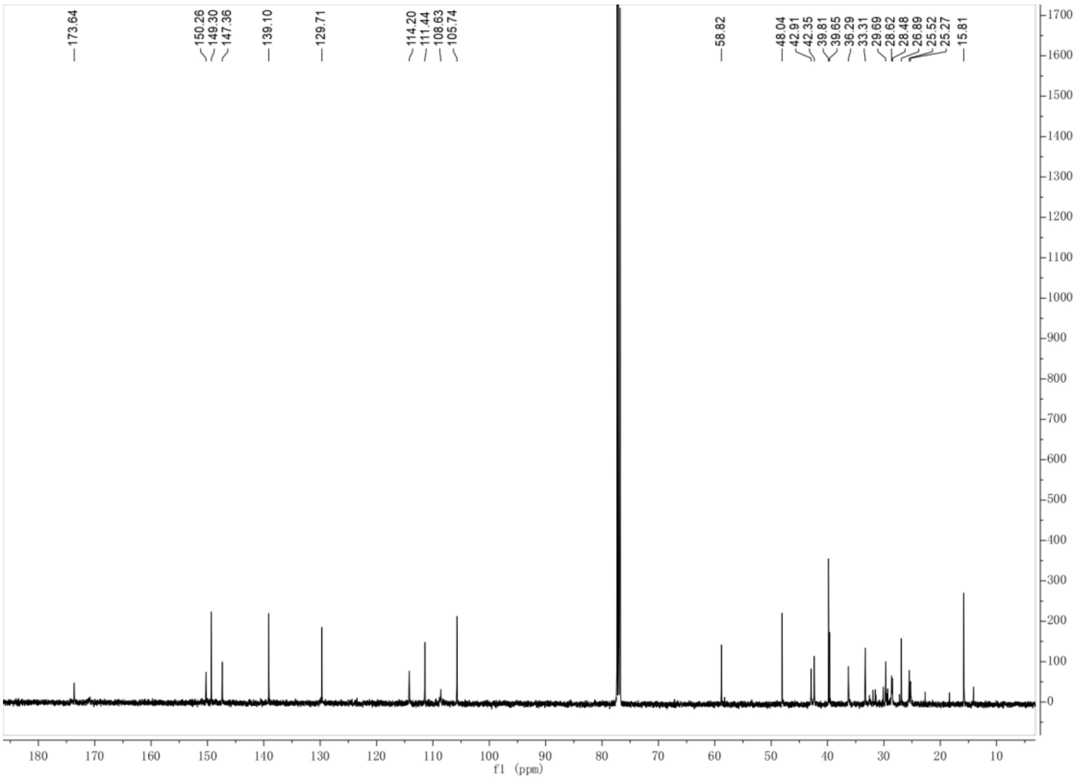

6-(4-(2-((1R,3R,4S)-3-(3-(1H-pyrazol-1-yl)prop-1-en-2-yl)-4-methyl-4-vinylcyclohexy l)allyl)piperazin-1-yl)-N-hydroxy-6-oxohexanamide (**27d**)

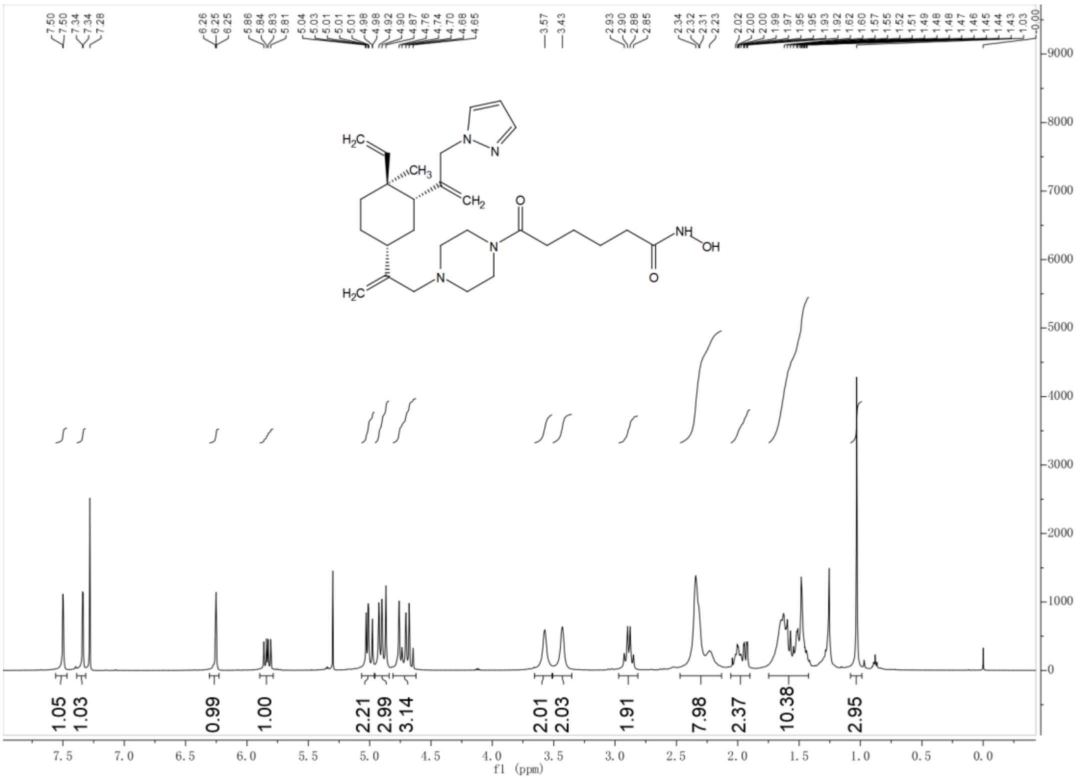

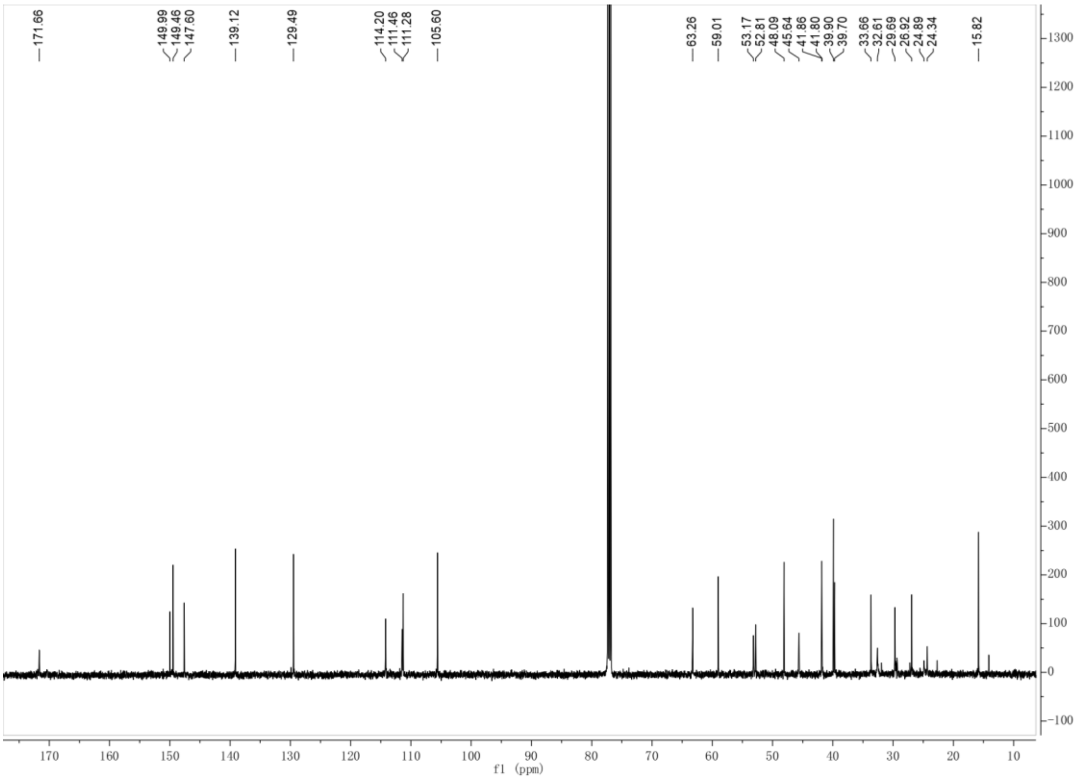

7-(4-(2-((1R,3R,4S)-3-(3-(1H-pyrazol-1-yl)prop-1-en-2-yl)-4-methyl-4-vinylcyclohexyl)allyl)piperazin-1-yl)-N-hydroxy-7-oxoheptanamide (**27e**)

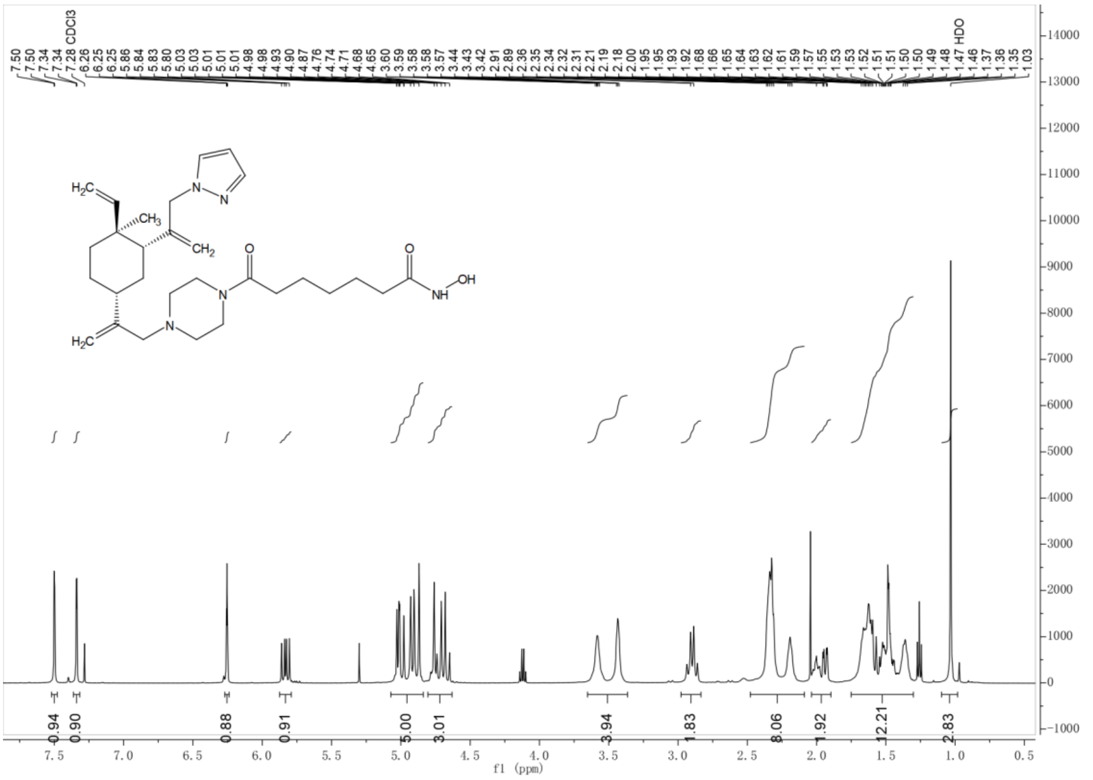

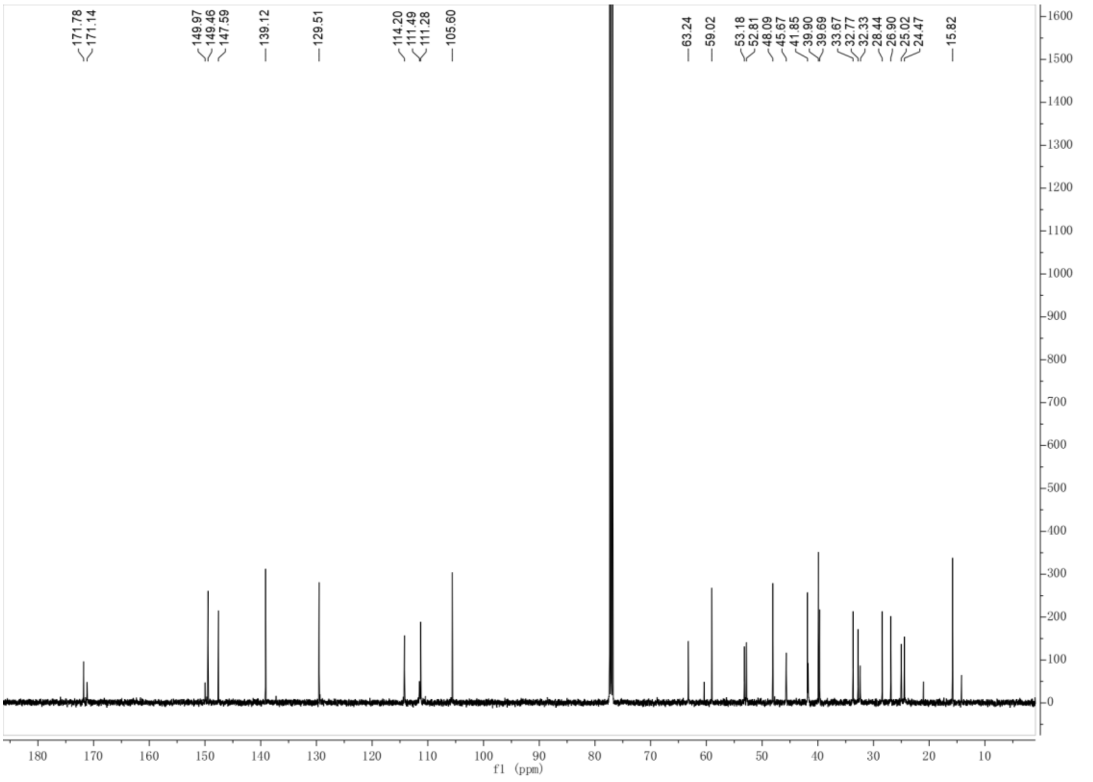

*8-(4-(2-((1R,3R,4S)-3-(3-(1H-pyrazol-1-yl)prop-1-en-2-yl)-4-methyl-4-vinylcyclohexyl)allyl)piperazin-1-yl)-N-hydroxy-8-oxooctanamide (*__27f__*)*

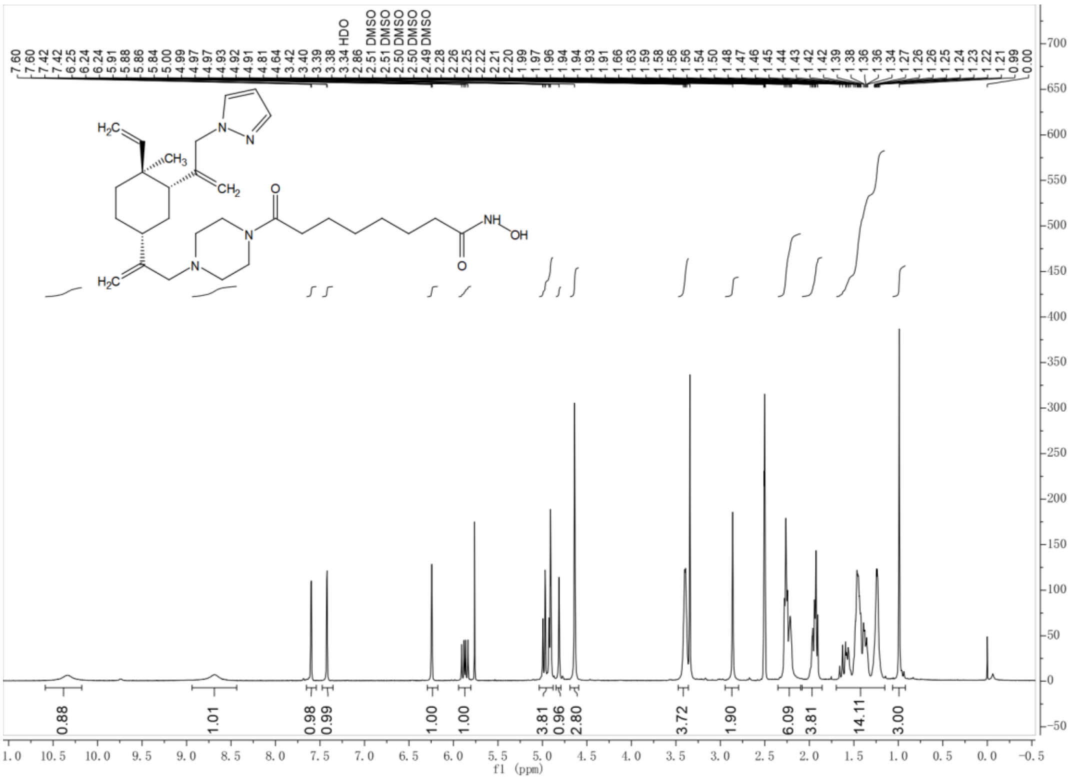

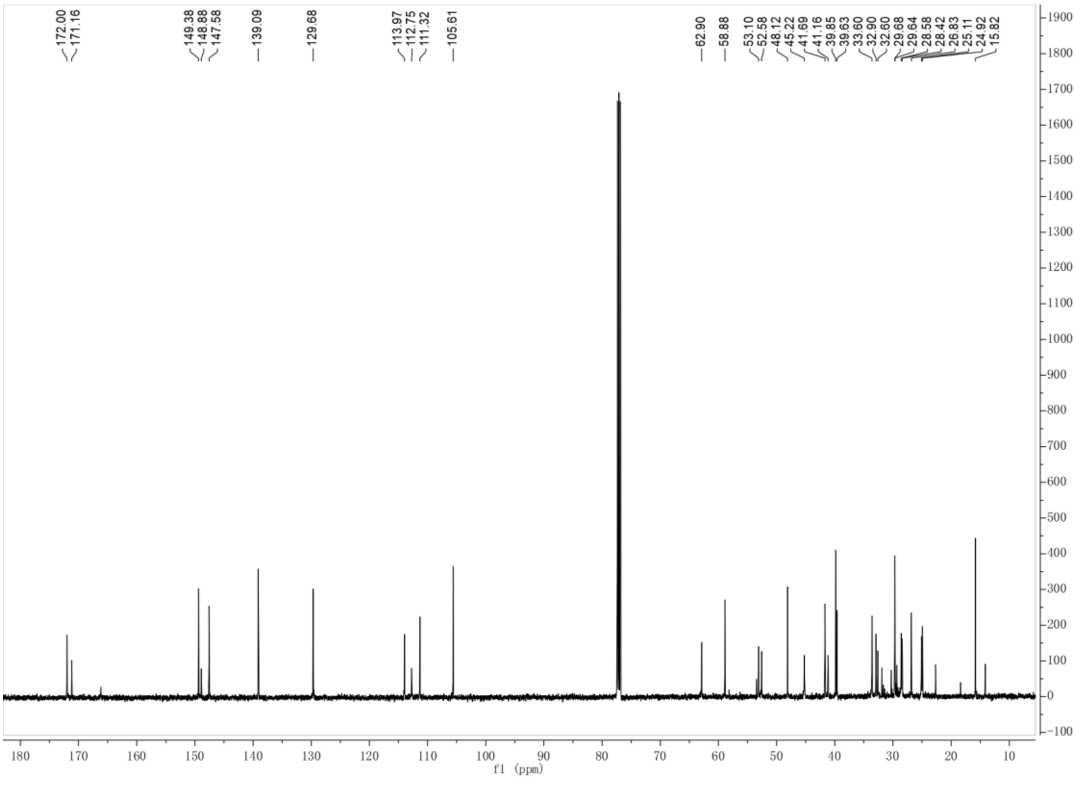

8-(4-(2-((1R,3R,4S)-3-(3-(1H-pyrazol-1-yl)prop-1-en-2-yl)-4-methyl-4-vinylcyclohexyl)allyl)piperazin-1-yl)-8-oxo-N-(pyridin-2-yl)octanamide (**31**)

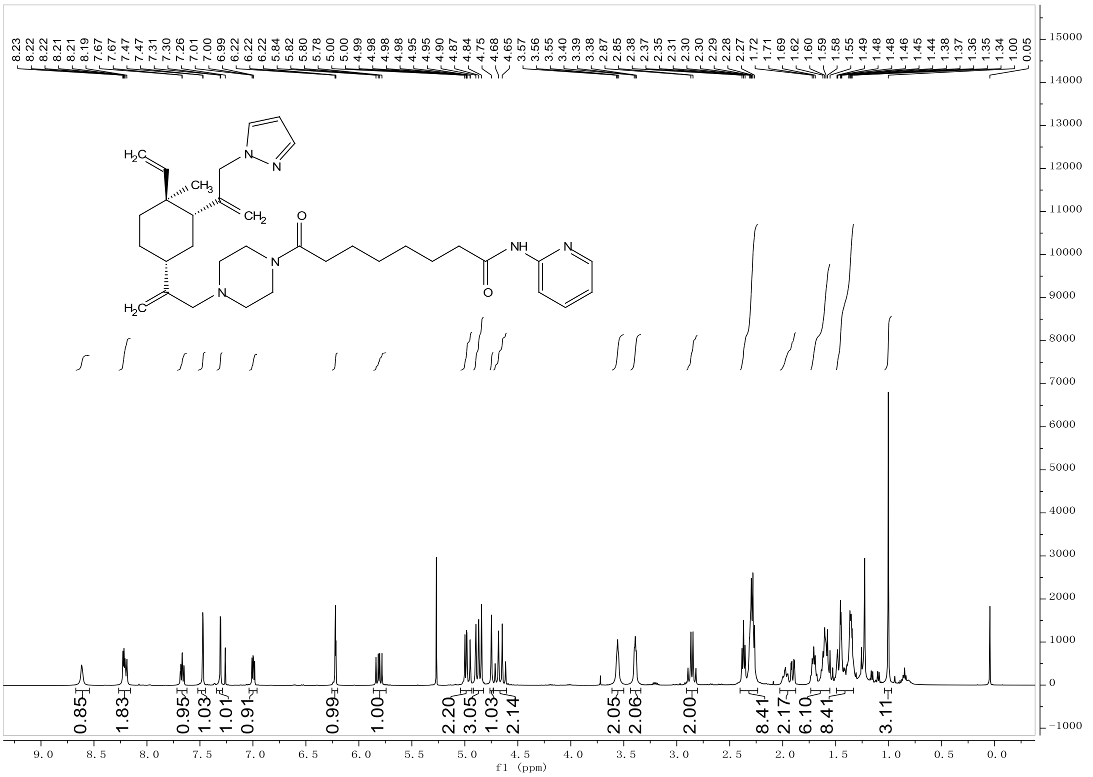

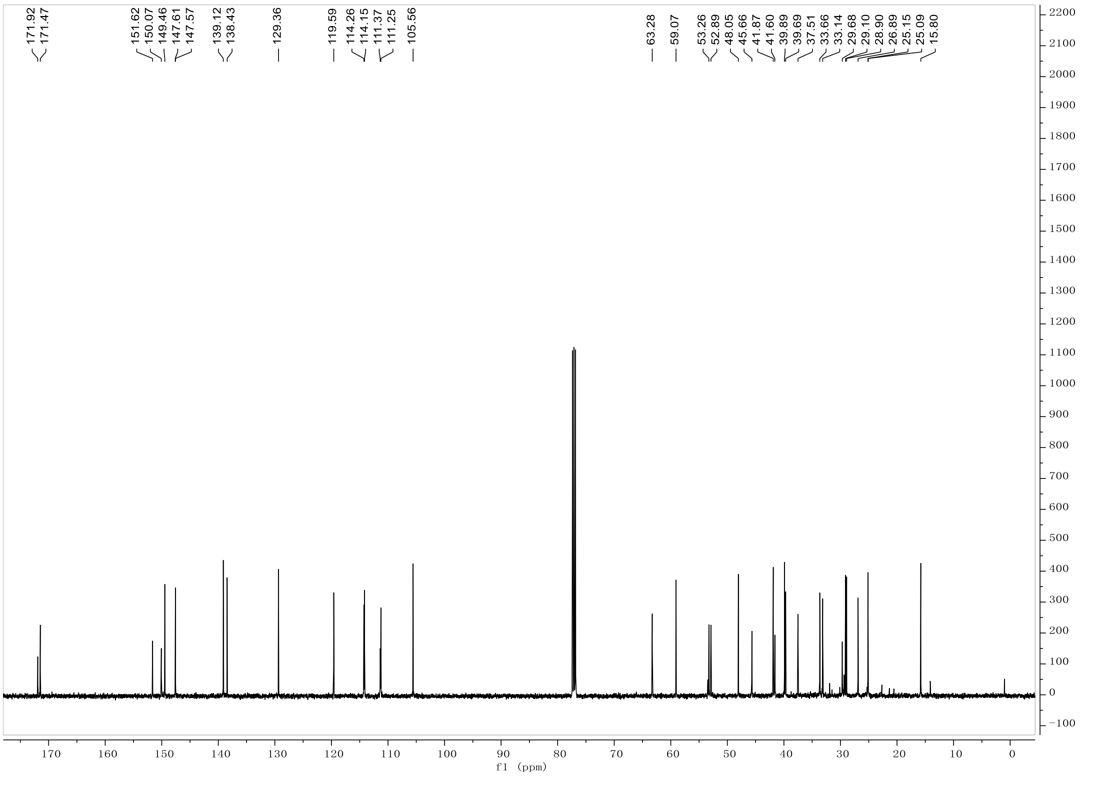

8-(4-(2-((1R,3R,4S)-3-(3-(1H-pyrazol-1-yl)prop-1-en-2-yl)-4-methyl-4-vinylcyclohexyl)allyl)piperazin-1-yl)-N-(2-aminophenyl)-8-oxooctanamide (**32**)

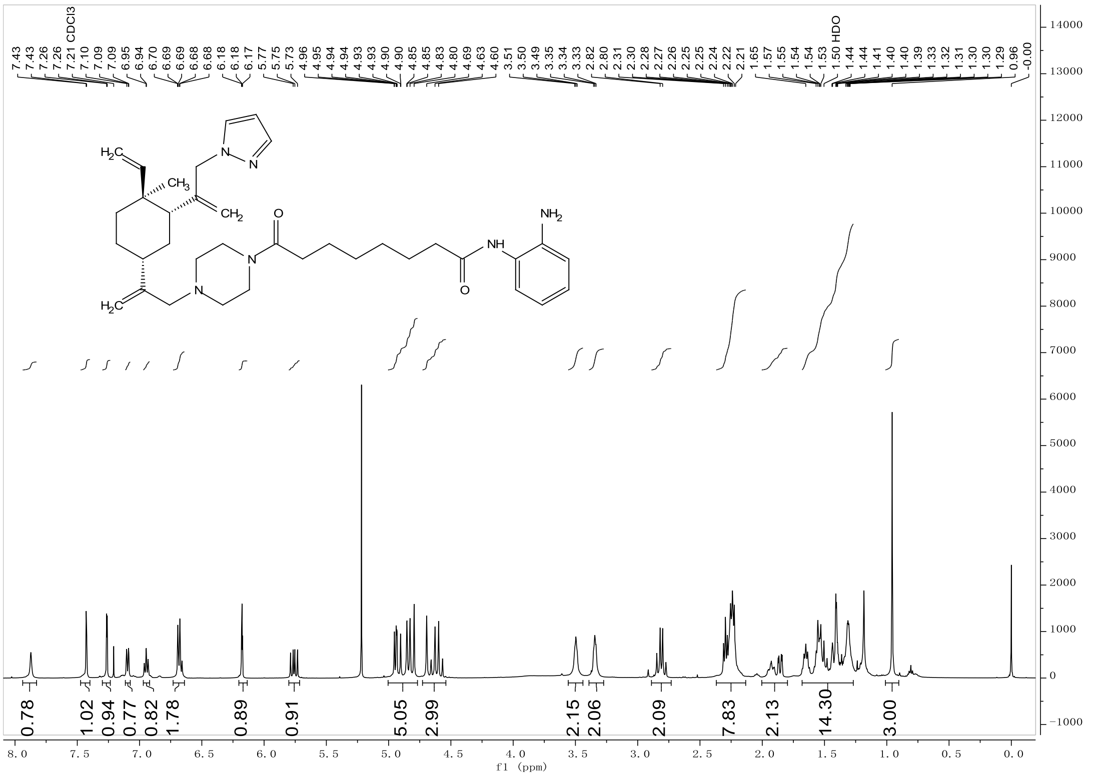

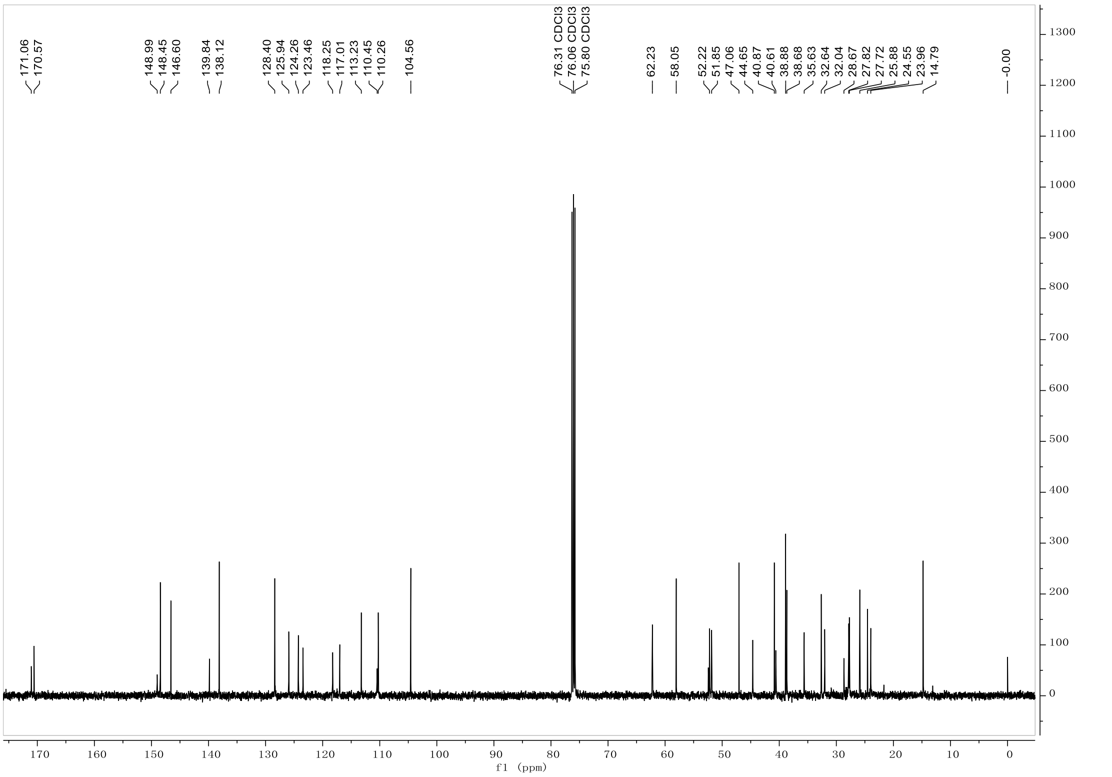

4-((4-(2-((1R,3R,4S)-3-(3-(1H-pyrazol-1-yl)prop-1-en-2-yl)-4-methyl-4-vinylcyclohexyl)allyl)piperazin-1-yl)methyl)-N-hydroxybenzamide (**34**)

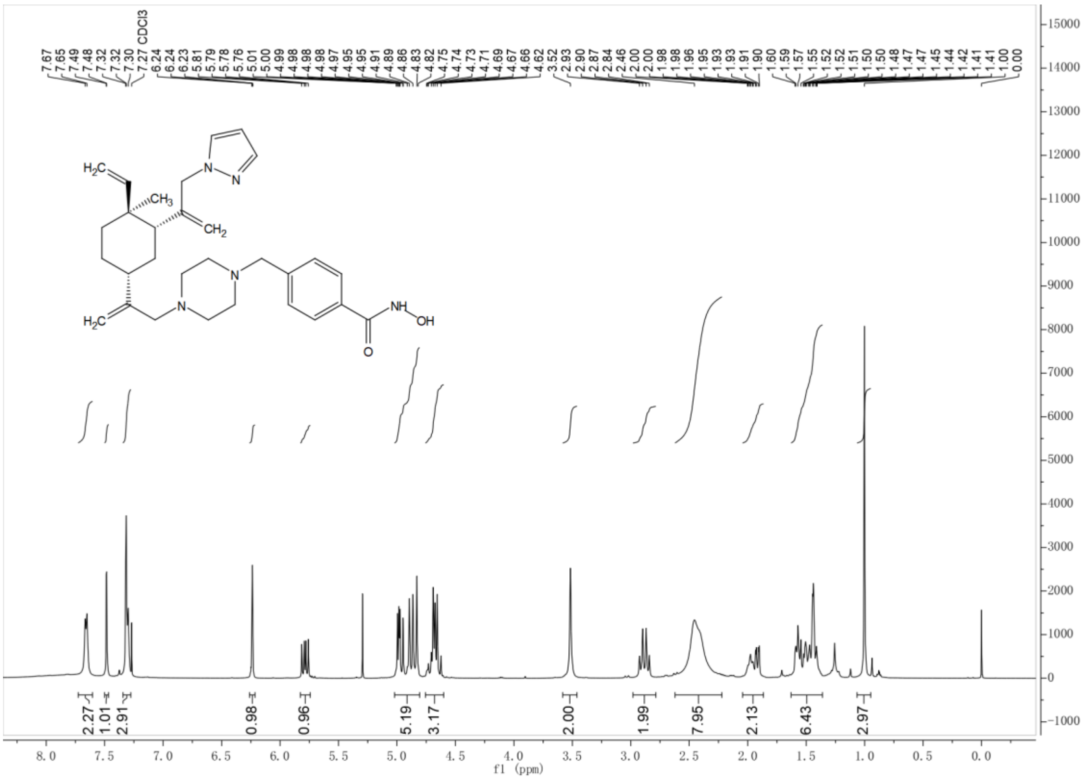

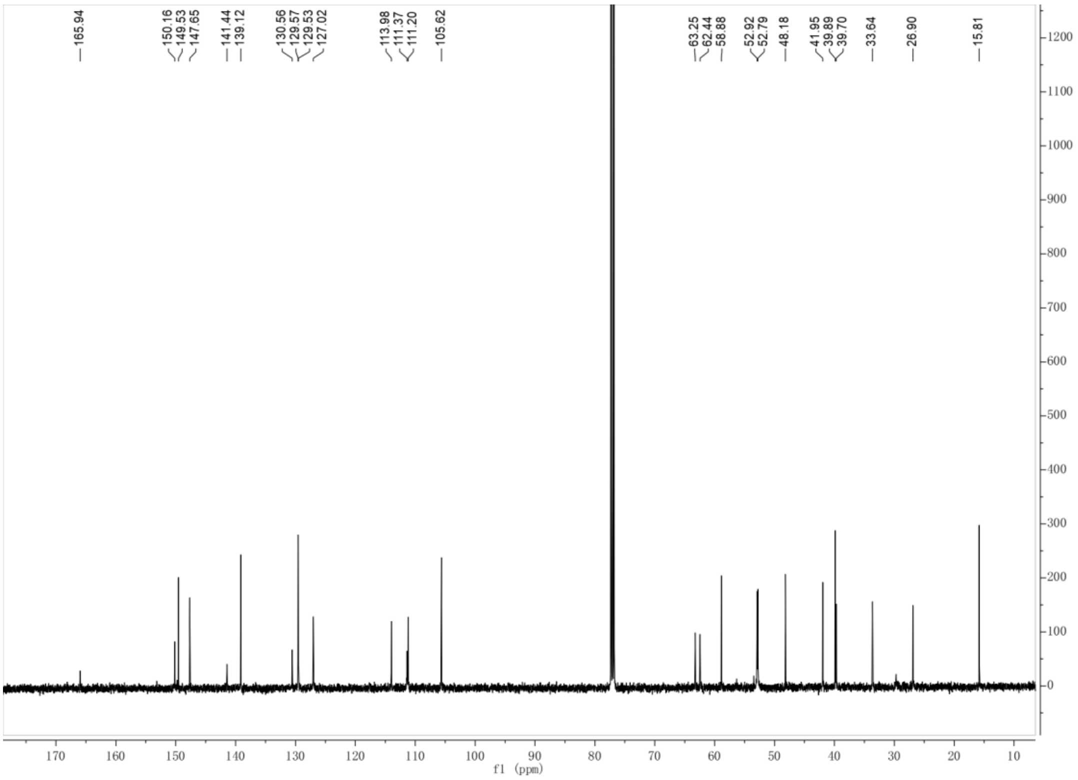

N[1]-(2-((1R,2S,5R)-5-(3-(1H-pyrazol-1-yl)prop-1-en-2-yl)-2-methyl-2-vinylcyclohexyl) allyl)-N[6]-hydroxyadipamide (**39a**)

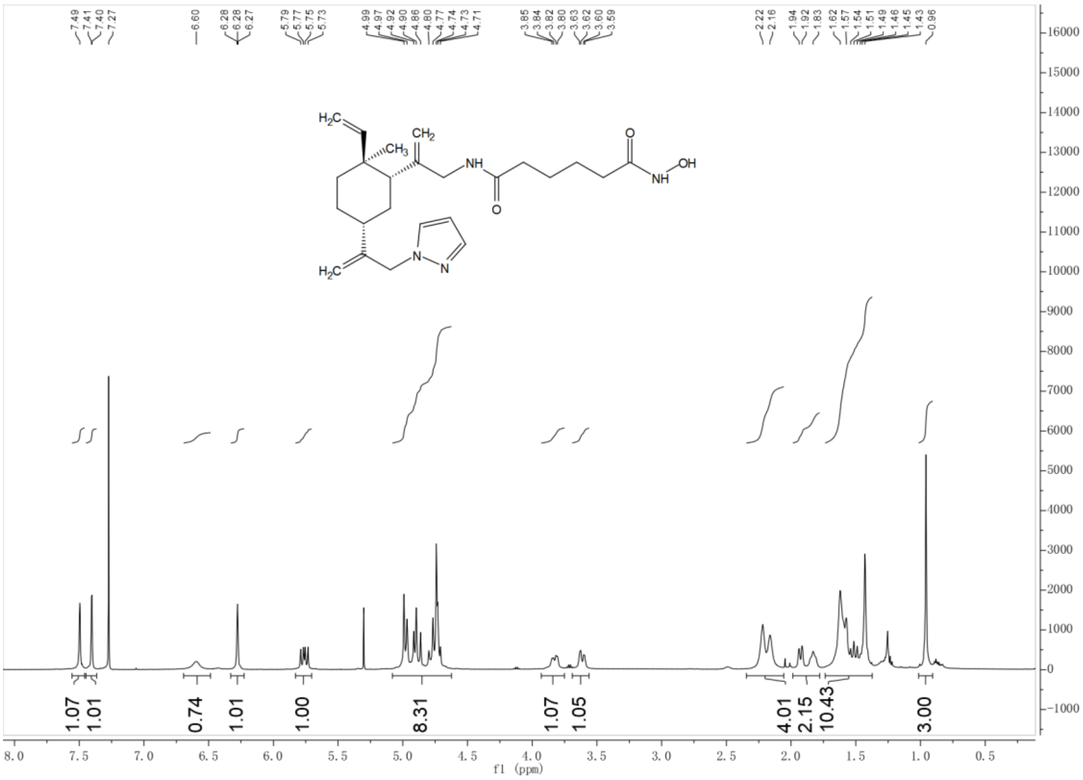

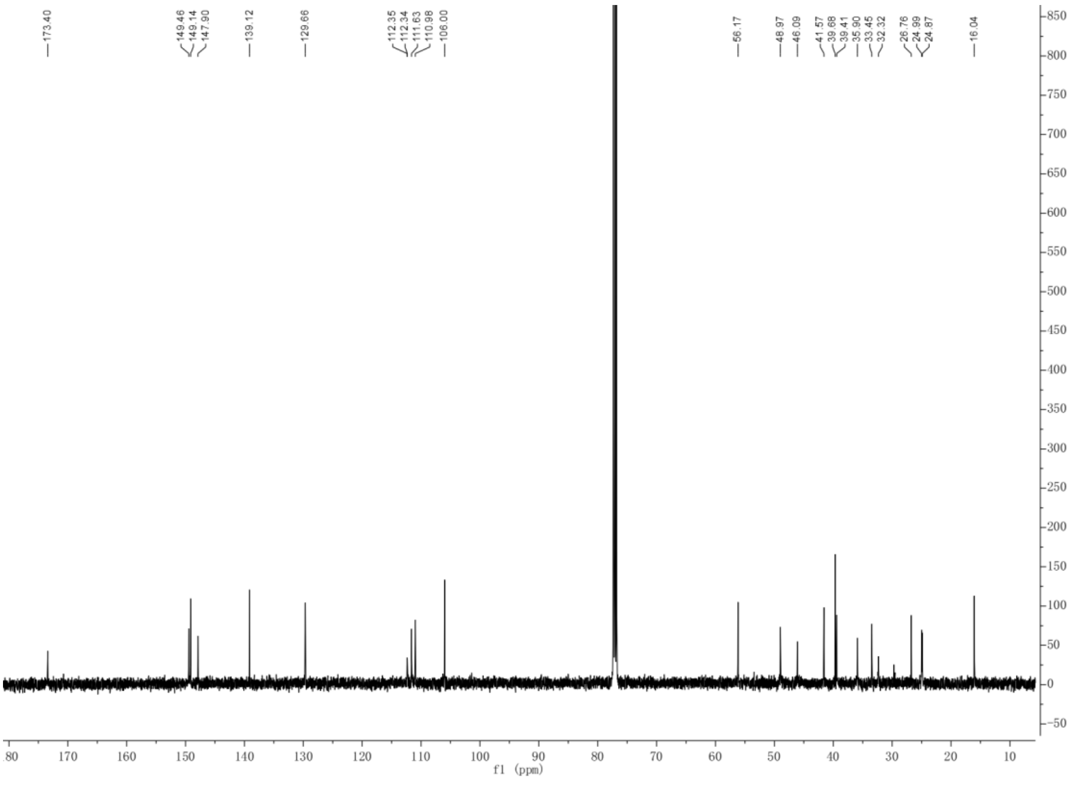

N[1]-(2-((1R,2S,5R)-5-(3-(1H-pyrazol-1-yl)prop-1-en-2-yl)-2-methyl-2-vinylcyclohexyl) allyl)-N[7]-hydroxyheptanediamide (**39b**)

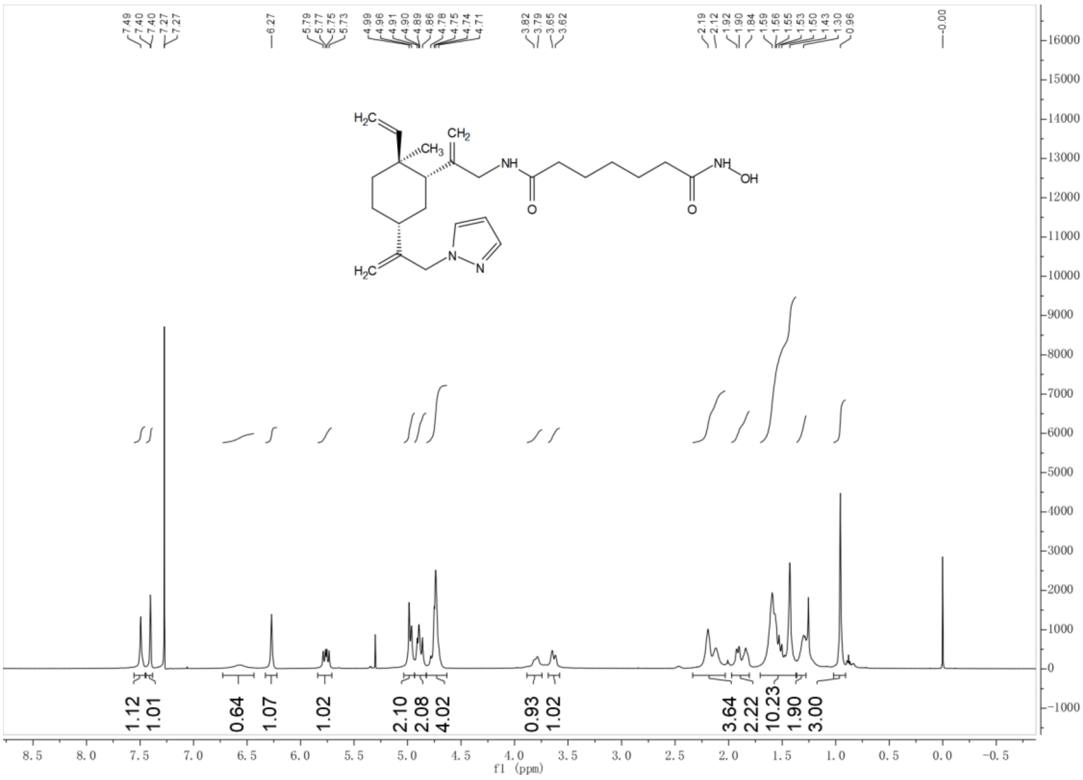

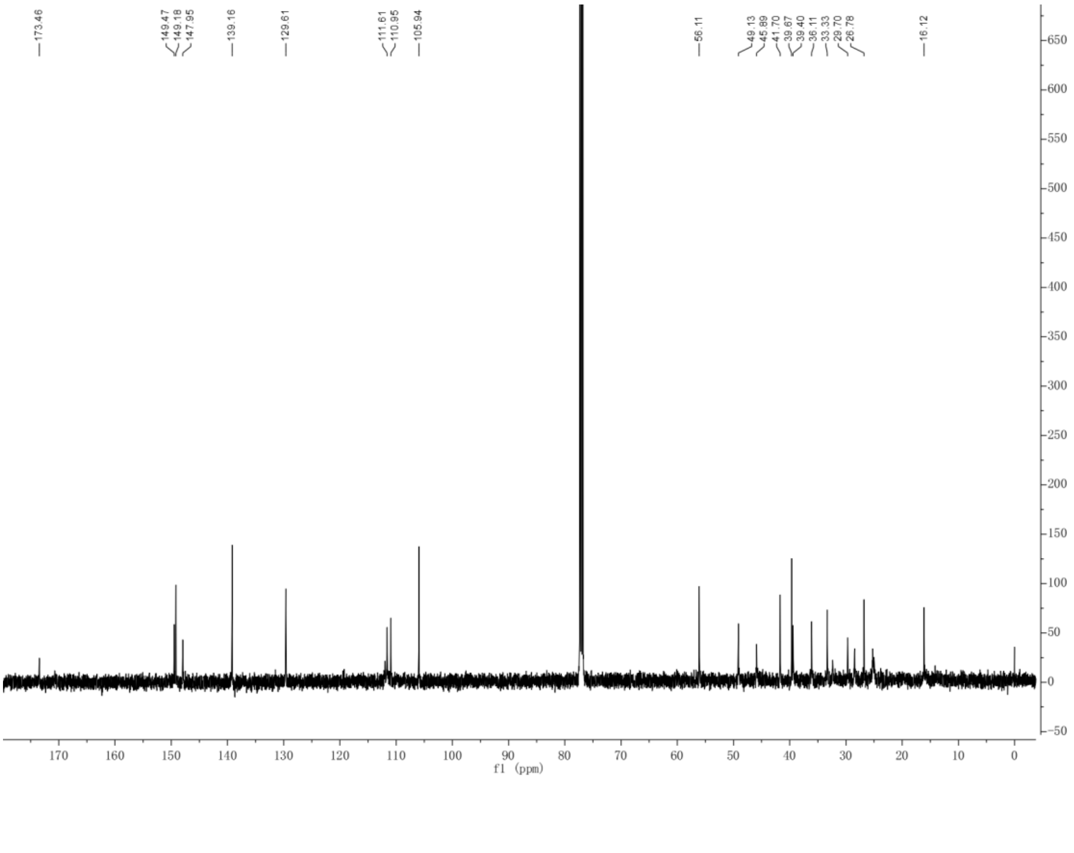

N[1]*-(2-((1R,2S,5R)-5-(3-(1H-pyrazol-1-yl)prop-1-en-2-yl)-2-methyl-2-vinylcyclohexyl) allyl)-N*[8]*-hydroxyoctanediamide* (**39c**)

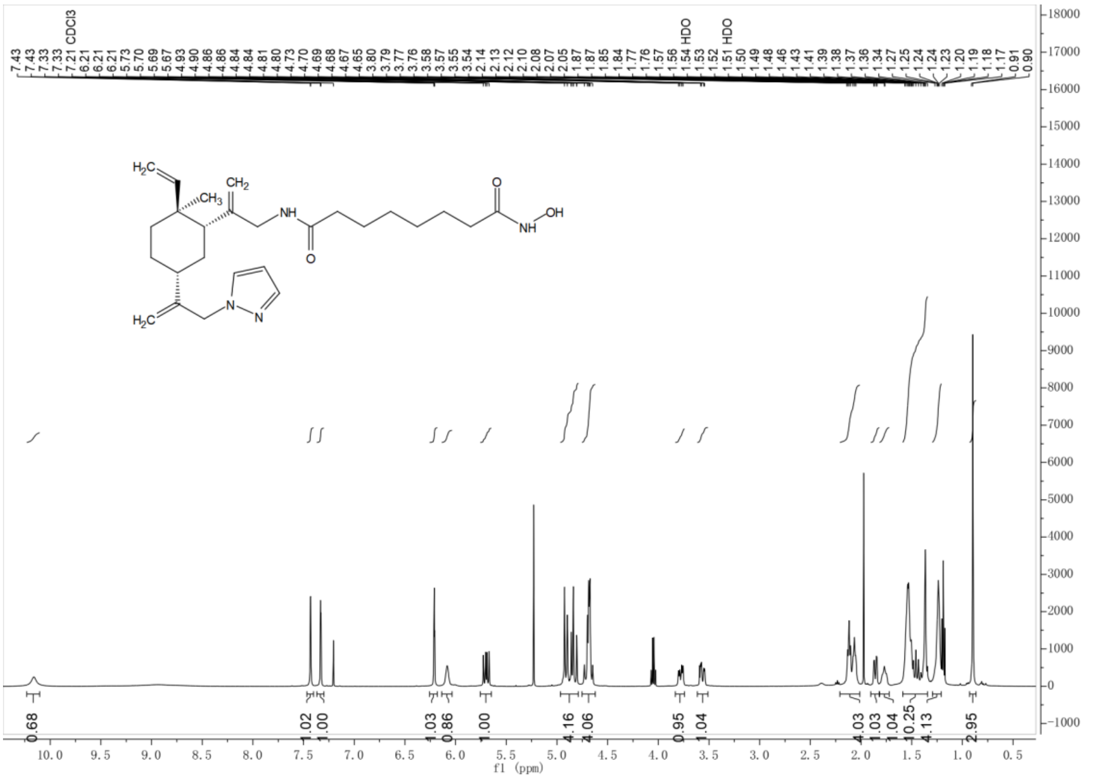

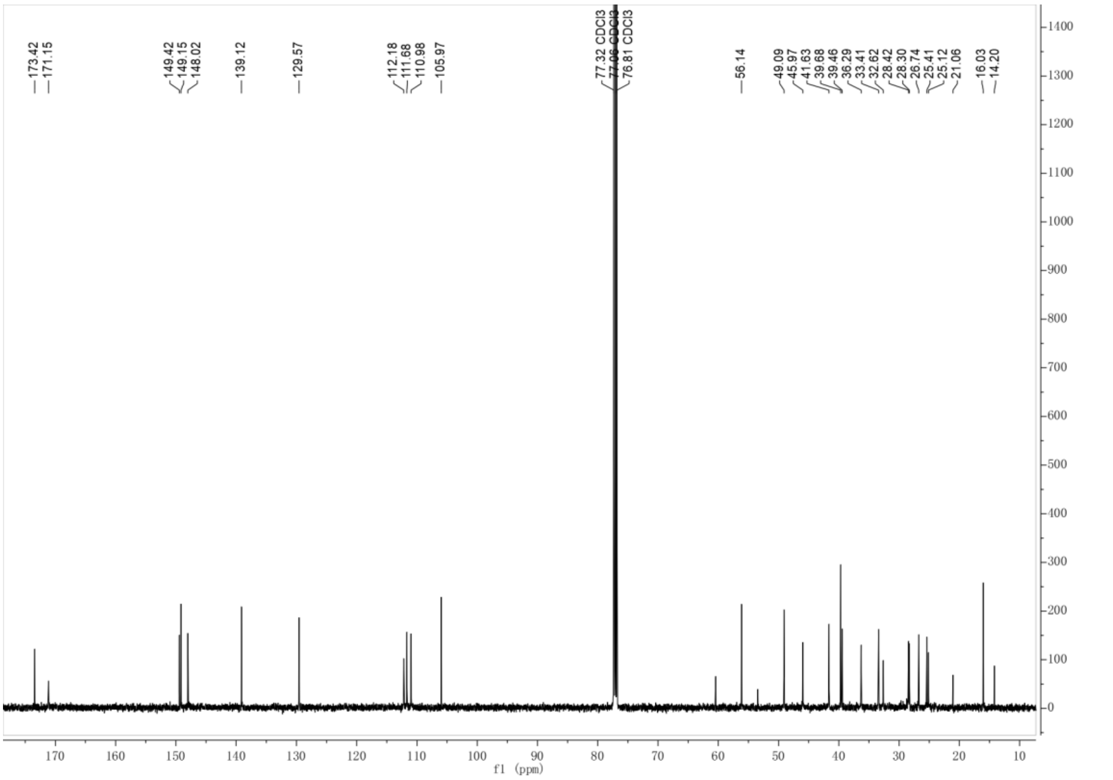

6-(4-(2-((1R,2S,5R)-5-(3-(1H-pyrazol-1-yl)prop-1-en-2-yl)-2-methyl-2-vinylcyclohexyl)allyl)piperazin-1-yl)-N-hydroxy-6-oxohexanamide (**39d**)

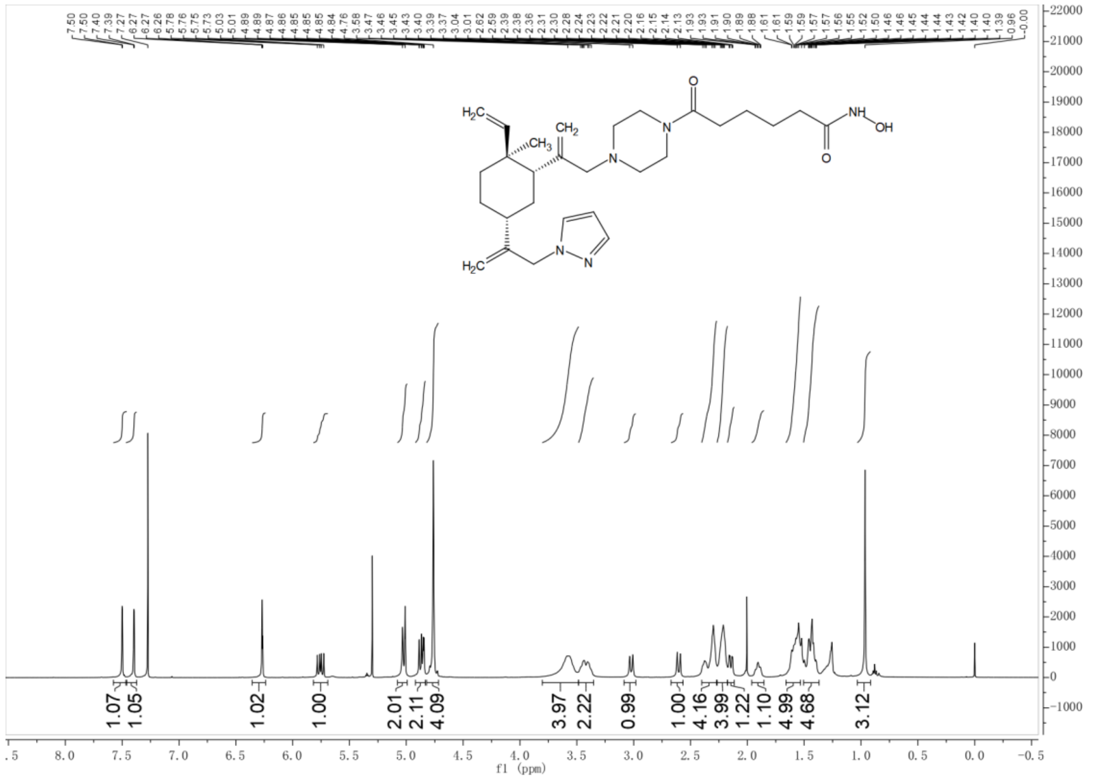

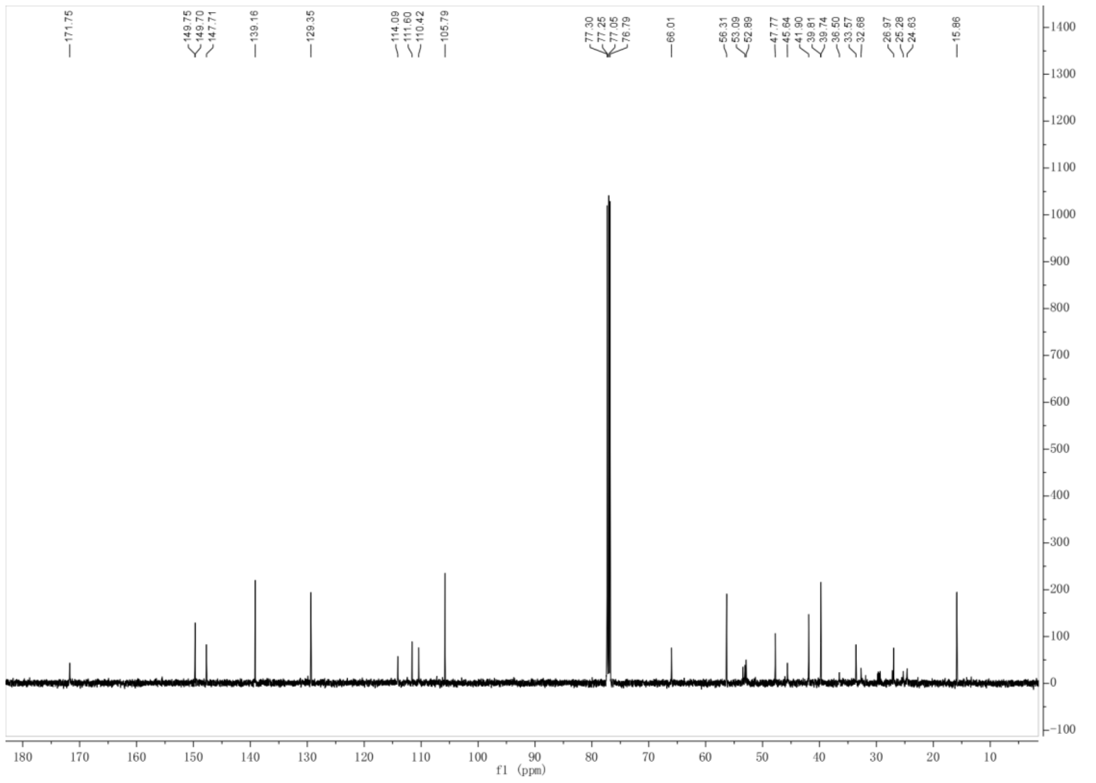

7-(4-(2-((1R,2S,5R)-5-(3-(1H-pyrazol-1-yl)prop-1-en-2-yl)-2-methyl-2-vinylcyclohexyl)allyl)piperazin-1-yl)-N-hydroxy-7-oxoheptanamide (**39e**)

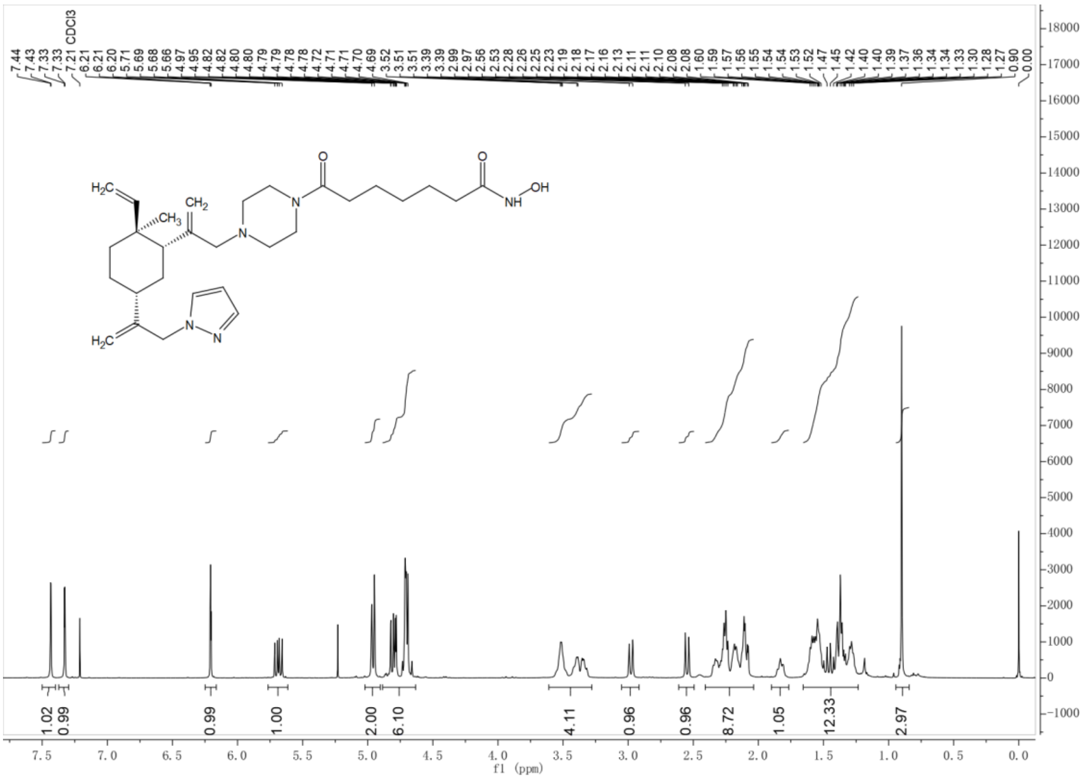

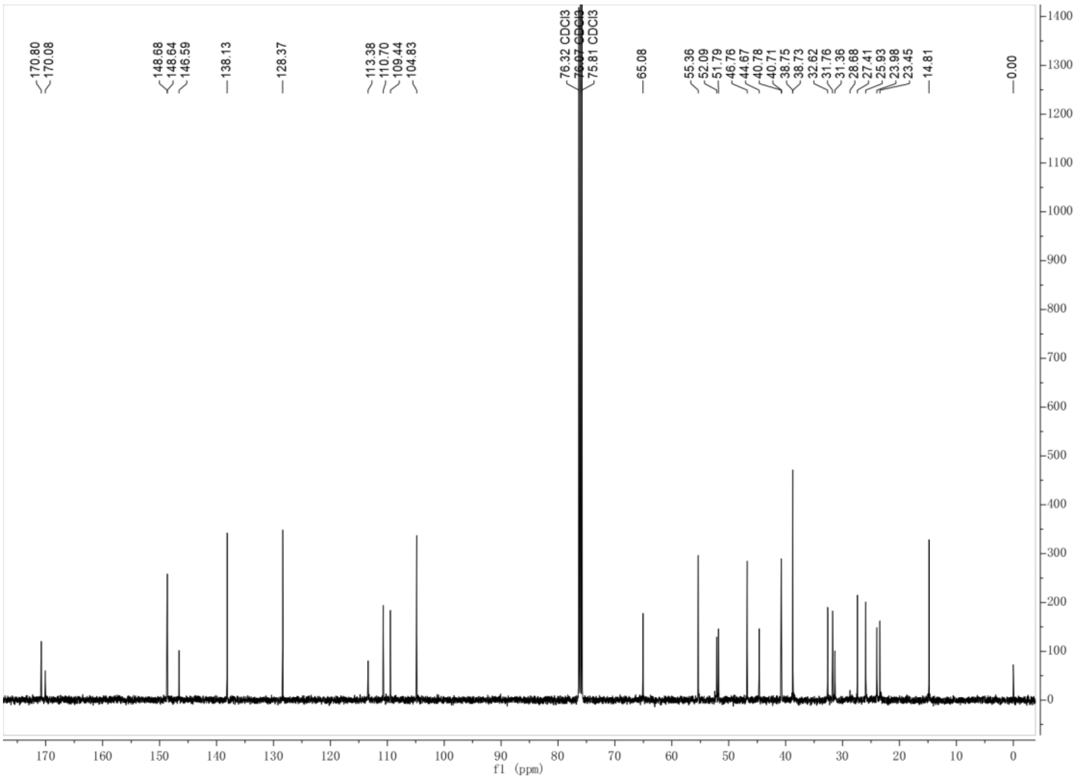

8-(4-(2-((1R,2S,5R)-5-(3-(1H-pyrazol-1-yl)prop-1-en-2-yl)-2-methyl-2-vinylcyclohexyl)allyl)piperazin-1-yl)-N-hydroxy-8-oxooctanamide (**39f**)

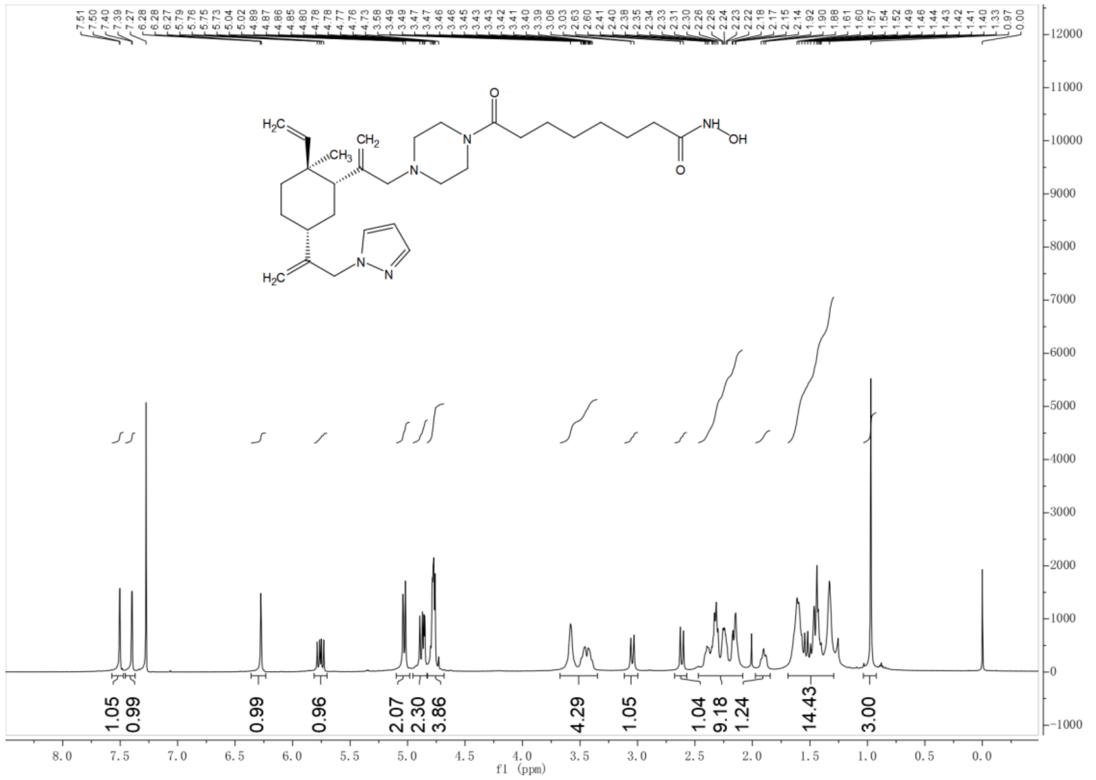

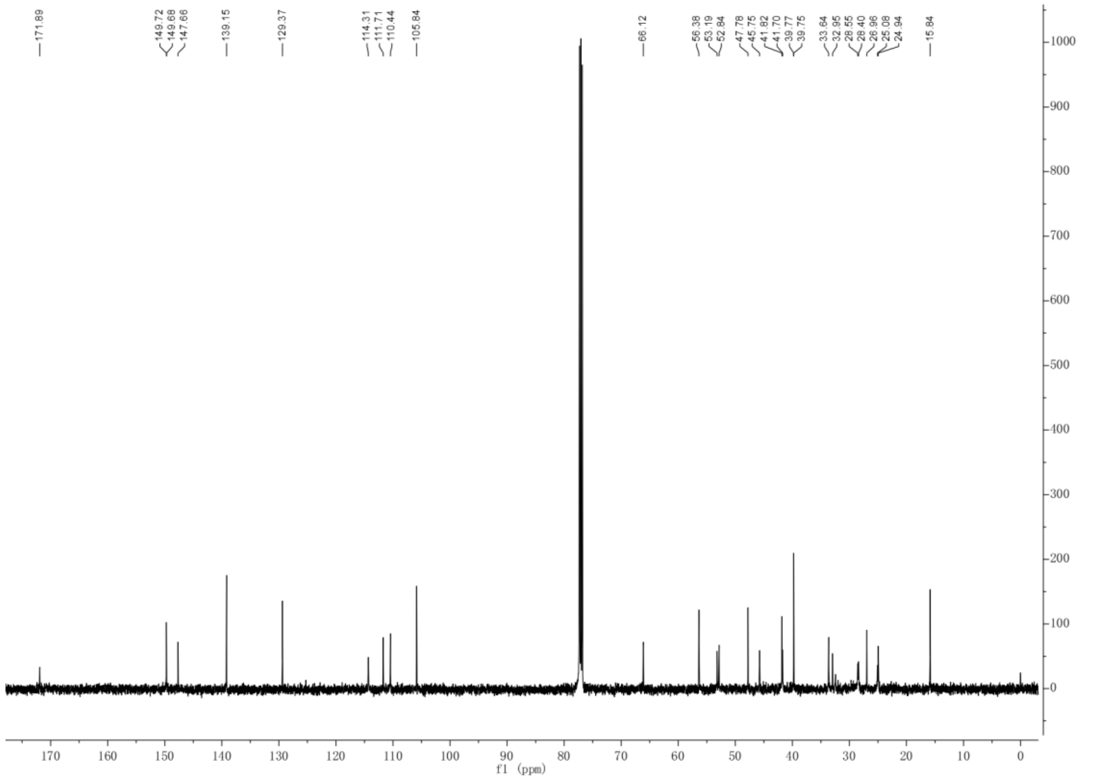

N[1]-(2-((1R,2S,5R)-5-(3-(1H-pyrazol-1-yl)prop-1-en-2-yl)-2-methyl-2-vinylcyclohexyl) allyl)-N[8]-(2-aminophenyl)octanediamide (**41**)

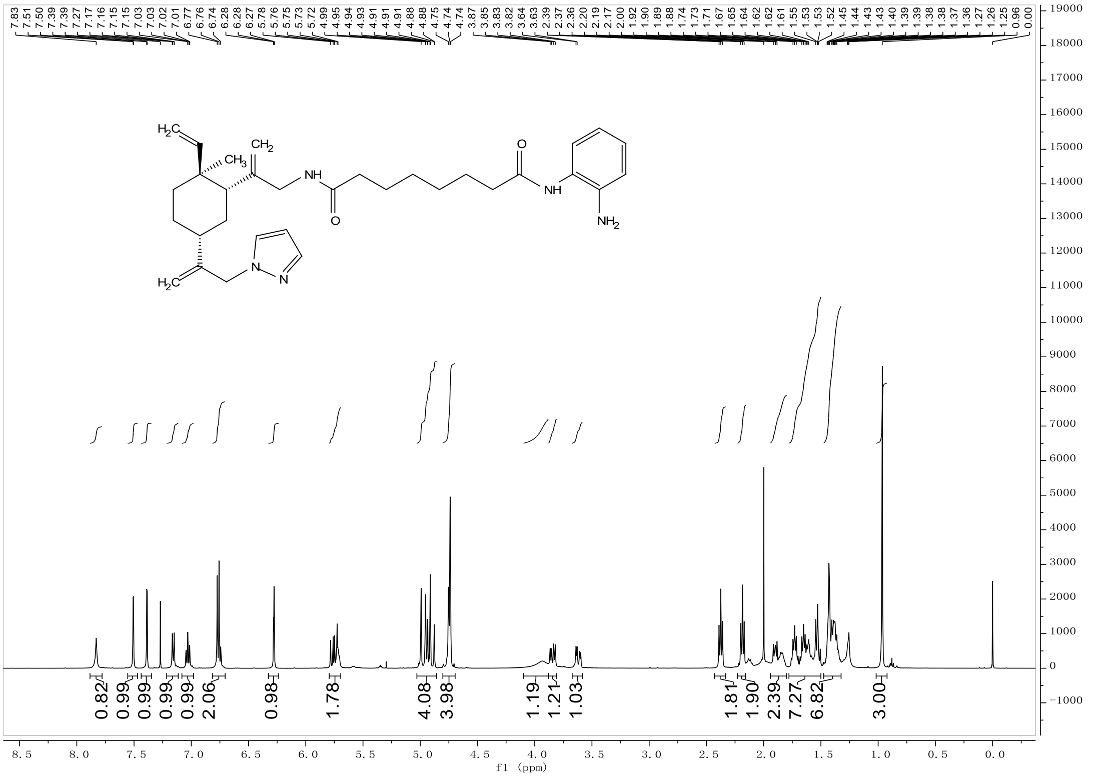

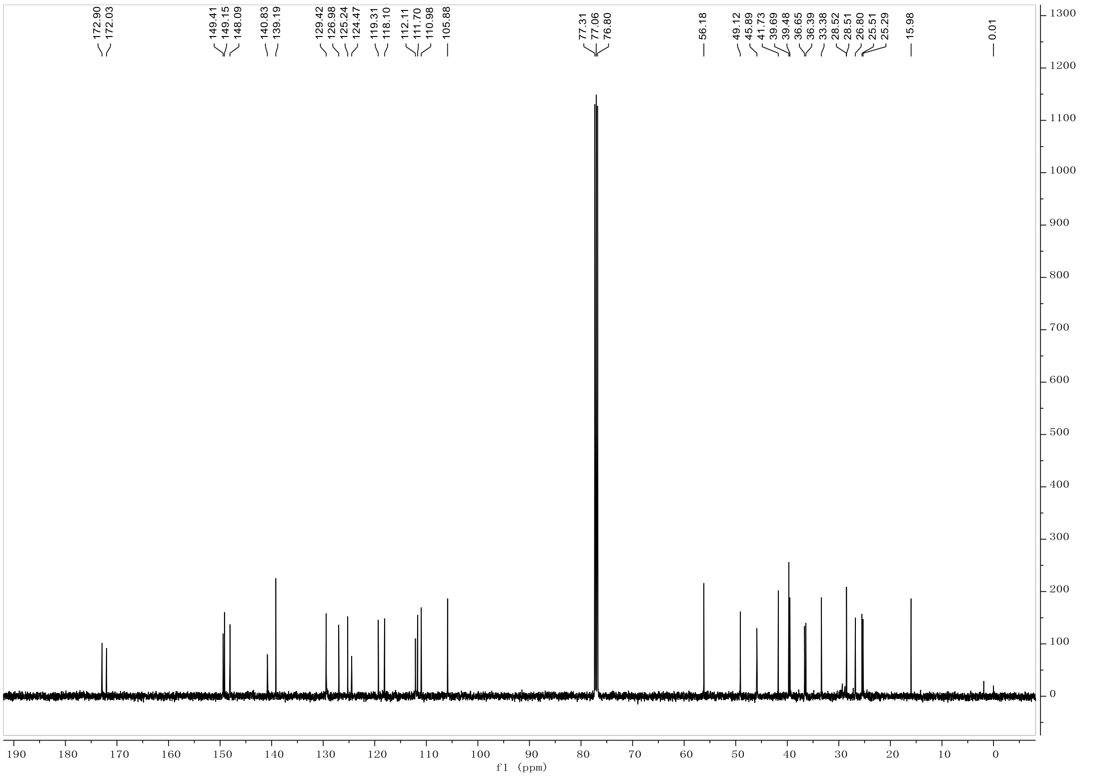

4-((4-(2-((1R,2S,5R)-5-(3-(1H-pyrazol-1-yl)prop-1-en-2-yl)-2-methyl-2-vinylcyclohexyl)allyl)piperazin-1-yl)methyl)-N-hydroxybenzamide (**43**)

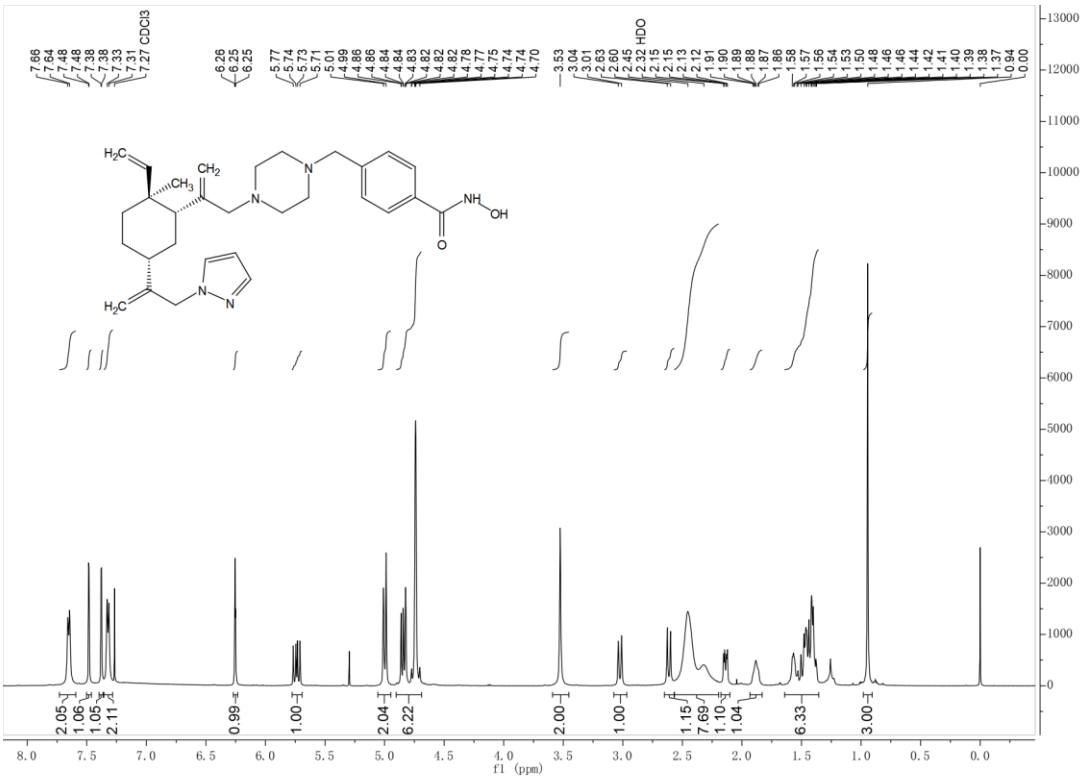

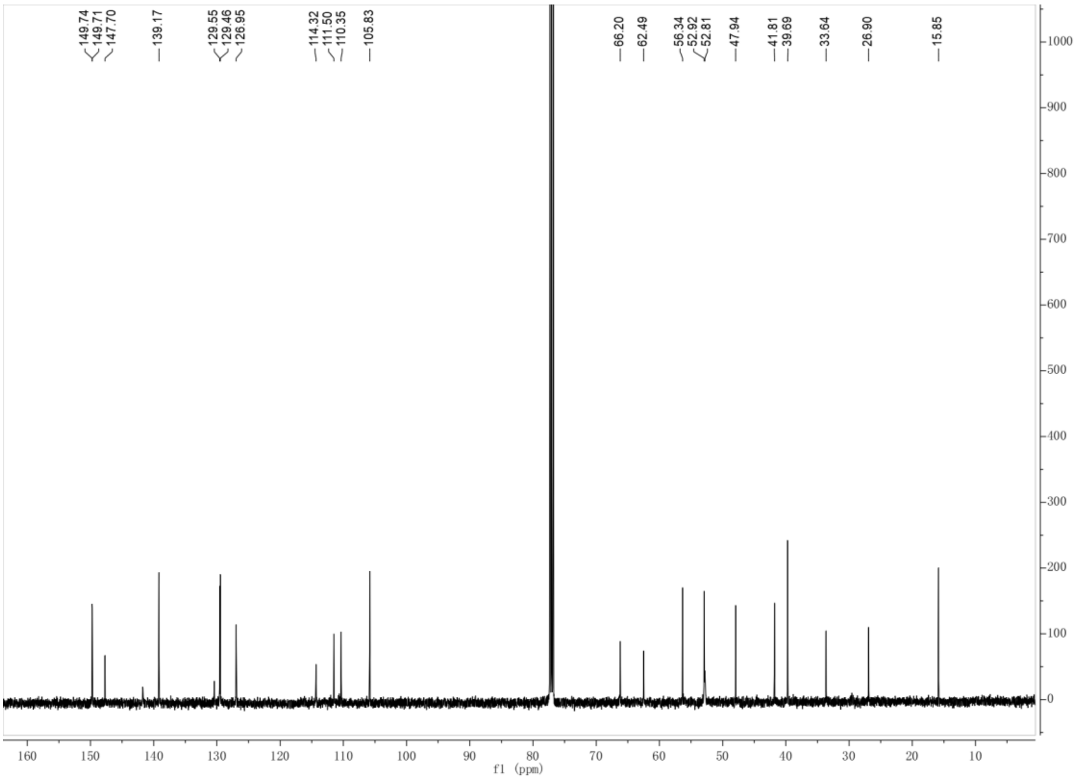

All the gating process figures related to cell apoptosis

ctrl:

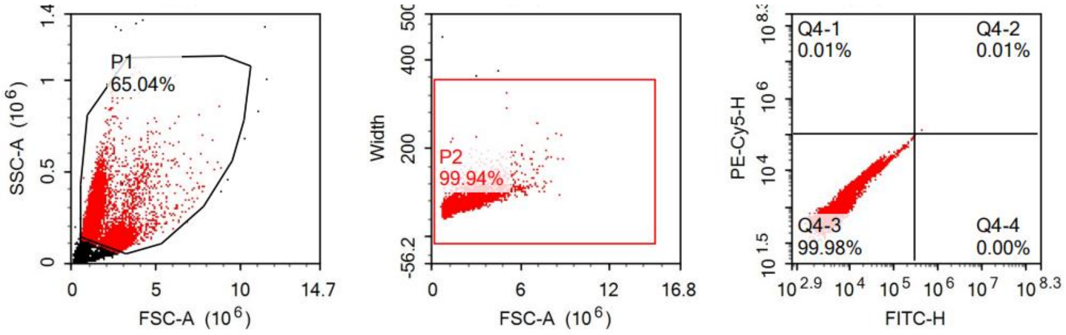

1 (*β*-elemene, 5 μM):

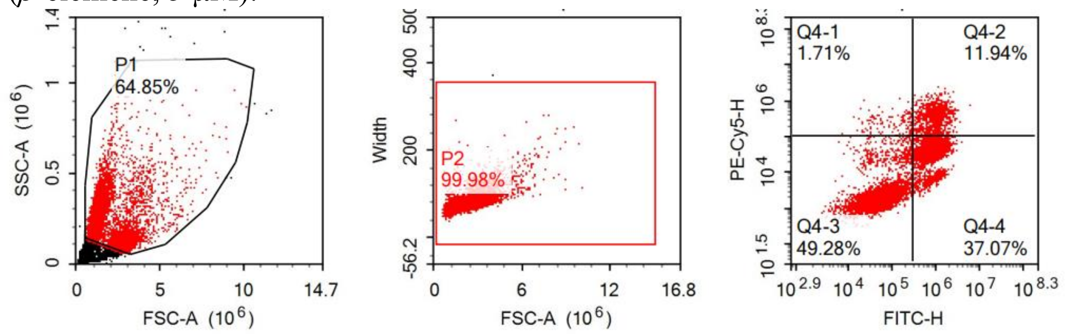

2 (SAHA, 5 μM):

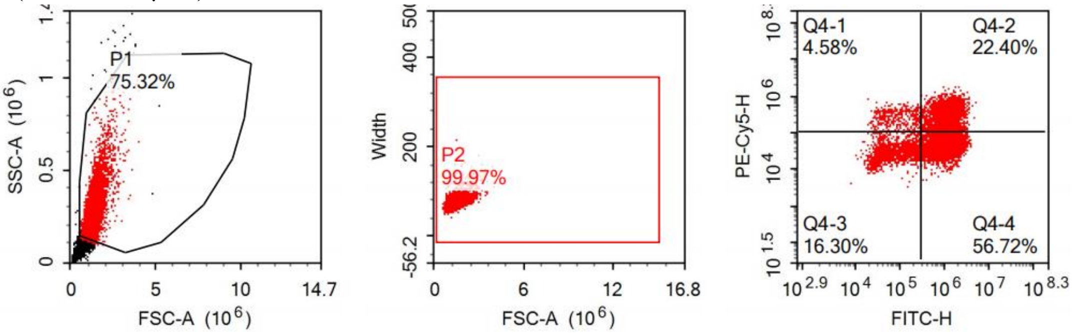

1 + **2** (1 : 1, 5 μM):

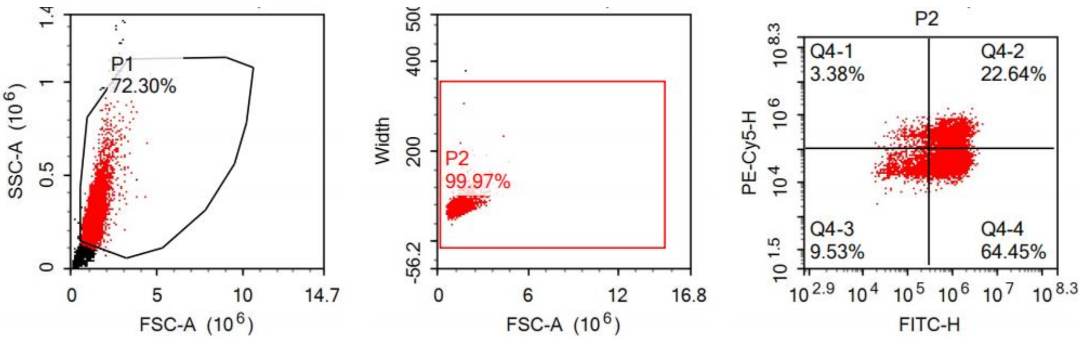

27f (5 μM):

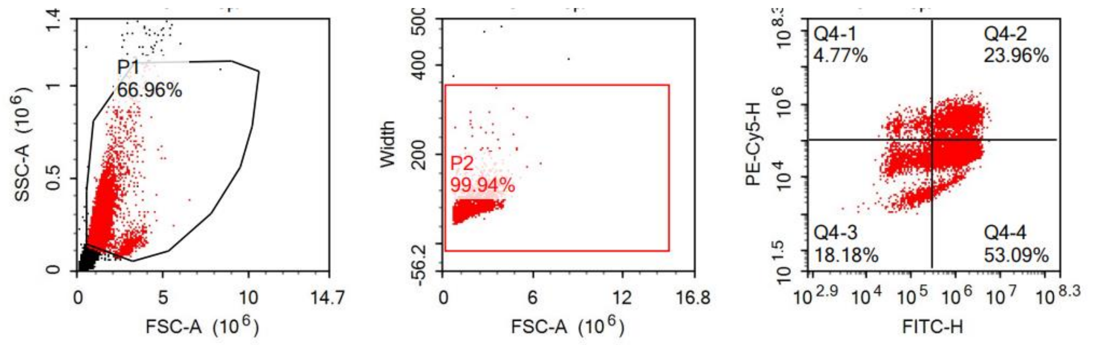

27f (10 μM):

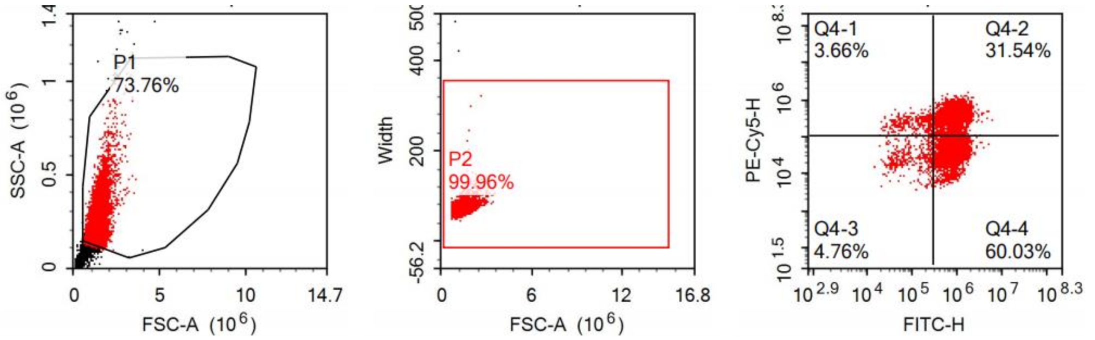

39f (5 μM):

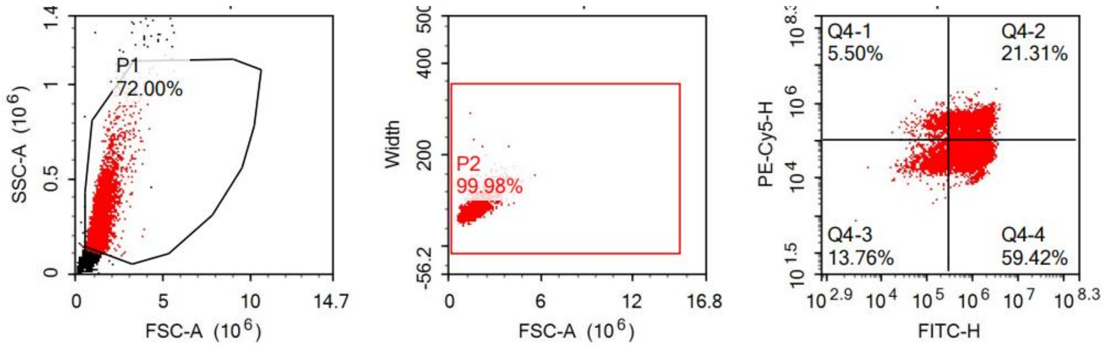

39f (10 μM):

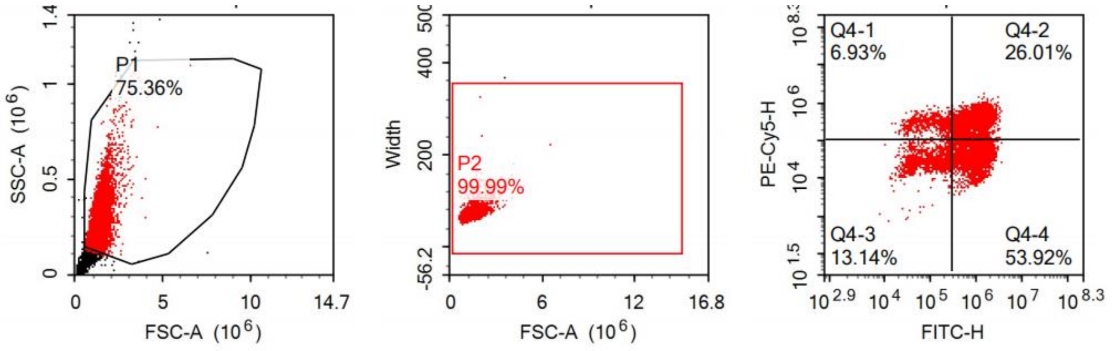

All the gating process figures related to cell cycle

ctrl:

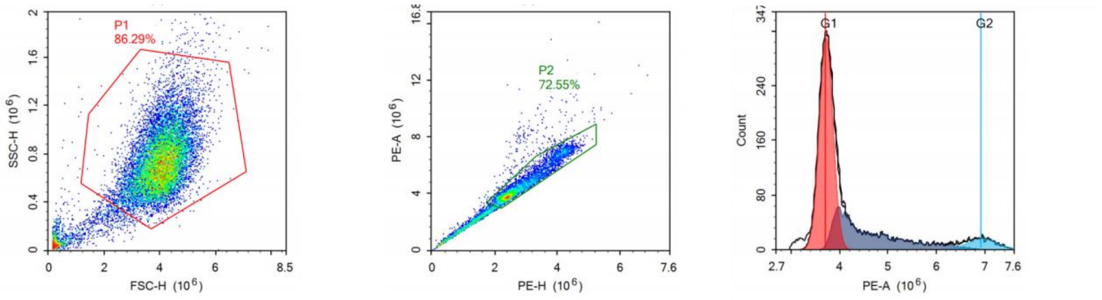

Gate	Count	% All	Median X	Median Y
All	15,972	100.00%	3,965,022	679,571
P1	13,783	86.29%	4,063,542	717,351

Gate	Count	% P1	Median X	Median Y
P1	13,783	100.00%	2,364,159	3,744,392
P2	10,000	72.55%	2,433,170	3,848,527

RMS	Freq G1	Freq S	Freq G2	G2/G1	CV G1
2.03	59.58	32.25	6.62	1.86	3.79%

1 (*β*-elemene, 1 μM):

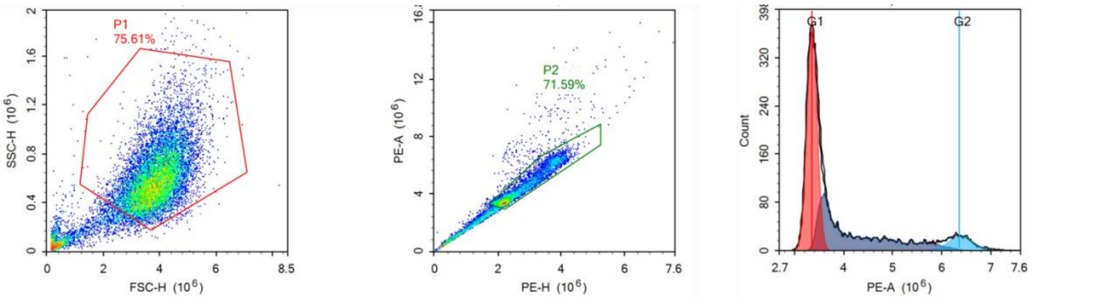

Gate	Count	% All	Median X	Median Y
All	18,474	100.00%	3,598,788	496,540
P1	13,969	75.61%	3,855,825	566,754

Gate	Count	% P1	Median X	Median Y
P1	13,969	100.00%	2,173,525	3,408,818
P2	10,000	71.59%	2,241,284	3,506,757

RMS	Freq G1	Freq S	Freq G2	G2/G1	CV G1
3.58	52.48	39.36	8.05	1.90	3.12%

2 (SAHA, 1 μM):

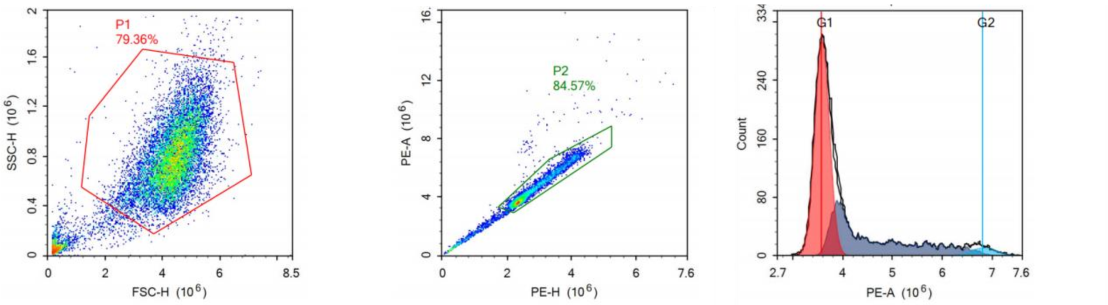

Gate	Count	% All	Median X	Median Y
All	14,900	100.00%	4,228,079	706,051
P1	11,825	79.36%	4,416,864	781,071

Gate	Count	% P1	Median X	Median Y
P1	11,825	100.00%	2,346,039	3,686,424
P2	10,000	84.57%	2,374,725	3,745,622

RMS	Freq G1	Freq S	Freq G2	G2/G1	CV G1
2.62	58.22	38.09	3.60	1.90	4.08%

1 + **2** (1 : 1, 1 μM):

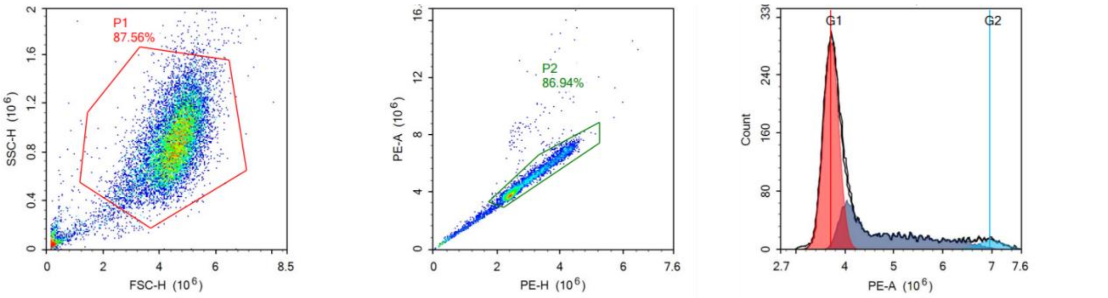

Gate	Count	% All	Median X	Median Y
All	13,136	100.00%	4,479,329	837,792
P1	11,502	87.56%	4,558,632	873,453

Gate	Count	% P1	Median X	Median Y
P1	11,502	100.00%	2,429,096	3,824,236
P2	10,000	86.94%	2,452,653	3,871,366

RMS	Freq G1	Freq S	Freq G2	G2/G1	CV G1
2.17	59.47	36.10	4.16	1.88	4.17%

27f (1 μM):

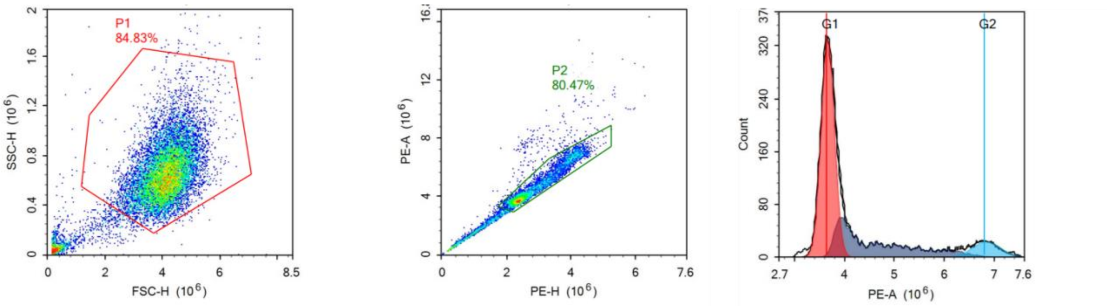

Gate	Count	% All	Median X	Median Y
All	14,649	100.00%	4,009,905	601,860
P1	12,427	84.83%	4,129,757	638,807

Gate	Count	% P1	Median X	Median Y
P1	12,427	100.00%	2,364,333	3,736,481
P2	10,000	80.47%	2,404,933	3,798,149

RMS	Freq G1	Freq S	Freq G2	G2/G1	CV G1
2.50	59.68	30.93	8.70	1.86	3.64%

27f (5 μM):

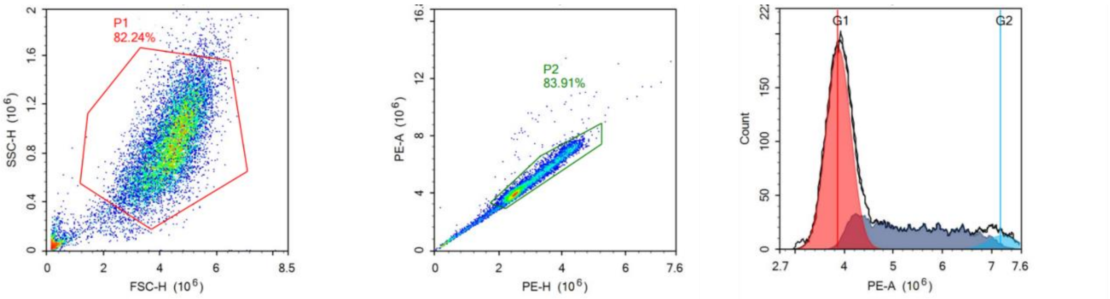

Gate	Count	% All	Median X	Median Y
All	14,491	100.00%	4,321,320	823,503
P1	11,917	82.24%	4,471,319	886,568

Gate	Count	% P1	Median X	Median Y
P1	11,917	100.00%	2,506,802	3,992,795
P2	10,000	83.91%	2,551,759	4,082,171

RMS	Freq G1	Freq S	Freq G2	G2/G1	CV G1
1.49	61.19	34.35	4.45	1.85	6.39%

39f (1 μM):

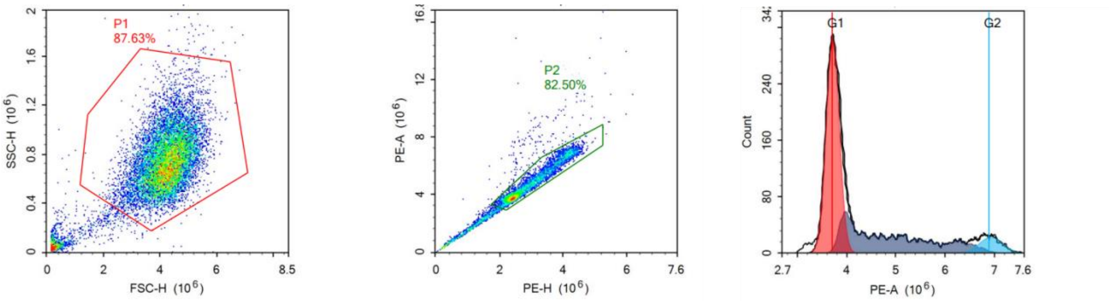

Gate	Count	% All	Median X	Median Y
All	13,832	100.00%	4,159,569	695,698
P1	12,121	87.63%	4,254,543	729,498

Gate	Count	% P1	Median X	Median Y
P1	12,121	100.00%	2,421,074	3,814,553
P2	10,000	82.50%	2,462,668	3,877,943

RMS	Freq G1	Freq S	Freq G2	G2/G1	CV G1
2.89	54.55	37.21	7.07	1.86	3.55%

39f (5 μM):

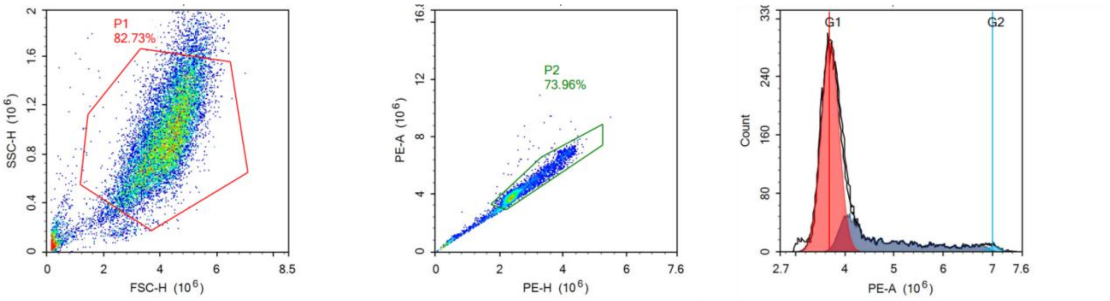

Gate	Count	% All	Median X	Median Y
All	16,344	100.00%	4,156,001	899,194
P1	13,521	82.73%	4,258,915	935,230

Gate	Count	% P1	Median X	Median Y
P1	13,521	100.00%	2,324,511	3,692,799
P2	10,000	73.96%	2,373,787	3,814,540

RMS	Freq G1	Freq S	Freq G2	G2/G1	CV G1
4.09	70.18	27.83	1.31	1.90	4.80%
